# Transcriptional and epigenetic regulation of autophagy: mechanisms, disease relevance and therapeutic opportunities

**DOI:** 10.1038/s41392-026-02688-3

**Published:** 2026-06-04

**Authors:** Jieun Seo, Seo-Young Park, Dong Chul Lee, Wonbeak Yoo, Yong Ryoul Yang, Hwi Won Seo, Jong Lyul Park, Jae-Yeol Joo, Kyung Chan Park, Sangwon Byun

**Affiliations:** 1 Genomic Medicine Research Center, Korea Research Institute of Bioscience and Biotechnology (KRIBB), Daejeon, Republic of Korea; 2 Department of Functional Genomics, University of Science and Technology, Daejeon, Republic of Korea; 3 Aging Convergence Research Center, Korea Research Institute of Bioscience and Biotechnology (KRIBB), Daejeon, Republic of Korea; 4 Infectious Disease Research Center, Korea Research Institute of Bioscience and Biotechnology (KRIBB), Daejeon, Republic of Korea; 5 Department of Bioscience, University of Science and Technology, Daejeon, Republic of Korea; 6 Department of Pharmacy, College of Pharmacy, Hanyang University, Ansan, Gyeonggi-do Republic of Korea

**Keywords:** Epigenetics, Target validation

## Abstract

Autophagy is a tightly regulated catabolic process that is essential for cellular homeostasis, stress adaptation, and metabolic balance. Its dysregulation has been implicated in a wide range of diseases, including cancer, neurodegenerative disorders, metabolic syndromes, muscular diseases, and infections. Recent studies have revealed the central roles of transcription factors, including TFEB, FOXO family members, p53, and NF-κB, in orchestrating autophagy through their direct regulation of lysosome-related genes. These factors often interact with epigenetic regulators such as histone acetyltransferases, deacetylases, and methyltransferases, which fine-tune chromatin accessibility and transcriptional output. Dysregulation of these pathways leads to aberrant autophagy and contributes to pathogenesis. Emerging therapeutic strategies targeting these transcriptional and epigenetic regulators have shown promise in preclinical and clinical settings, although challenges remain owing to the context-specific roles of autophagy in promoting either cell survival or cell death or contributing to protein aggregation and metabolic imbalance, depending on the disease. Clinical trials with autophagy modulators, including mTOR inhibitors, HDAC inhibitors, SIRT1 activators, and TFEB agonists, have yielded variable outcomes, emphasizing the need for precision medicine approaches. Advances in nanomedicine and biomaterials provide innovative delivery platforms that increase the specificity, bioavailability, and tissue targeting ability of autophagy-targeting agents. This review provides a comprehensive and detailed synthesis of how transcriptional and epigenetic regulators control autophagy across physiological and pathological contexts. In addition, we discuss therapeutic efforts, challenges in clinical translation, and future directions, including biomarker discovery, combinatorial treatment strategies, and targeted delivery systems, to enable more effective modulation of autophagy in disease.

## Introduction

Autophagy is a highly conserved catabolic process that maintains cellular homeostasis by degrading damaged organelles, misfolded proteins, and excess cytoplasmic components through lysosome-mediated pathways. This dynamic process plays an essential role in cellular adaptation to stress, including nutrient deprivation, hypoxia, and metabolic imbalance. Once regarded as a nonselective degradation system, autophagy is currently understood to include selective processes tailored to specific cellular needs. Autophagic activity is intricately regulated at multiple levels, from cytoplasmic posttranslational modifications to nuclear transcriptional and epigenetic mechanisms, revealing its complexity and versatility in both physiological and pathological contexts.

Dysregulated autophagy is implicated in a broad range of diseases, including cancer, neurodegenerative disorders, cardiovascular and metabolic diseases, muscular dystrophies, infections, and aging. In cancer, autophagy plays dual roles by suppressing tumors during early carcinogenesis and supporting survival and therapy resistance in advanced stages. Similarly, in neurodegenerative diseases such as Alzheimer’s disease (AD) and Parkinson’s disease (PD), impaired clearance of toxic protein aggregates and defective mitophagy contribute to pathogenesis. In cardiovascular and metabolic disorders, autophagy regulates mitochondrial quality control, lipid metabolism, and inflammation. These context-specific roles emphasize the need for precise regulatory understanding and targeted therapeutic strategies.

The nuclear regulation of autophagy, especially the role of transcription factors and chromatin remodelers, has gained attention because of recent research advances. Key transcription factors, including TFEB, FOXO proteins, p53, and nuclear factor kappa-light-chain-enhancer of activated B (NF-κB), regulate autophagy-related and lysosomal genes. These proteins often cooperate with epigenetic regulators such as histone acetyltransferases, deacetylases, and methyltransferases to modulate chromatin accessibility and gene expression. Aberrant regulation at this level disrupts autophagy and contributes to disease progression.

As autophagy has emerged as a central player in health and disease, interest in its transcriptional and epigenetic control is increasing. This review provides a comprehensive overview of the molecular mechanisms underlying autophagy regulation, with a focus on both transcriptional and epigenetic factors. In addition, we highlight emerging therapeutic strategies, including small-molecule modulators and nanomedicine-based delivery systems, and discuss the challenges and opportunities in clinical translation.

## Core processes and disease relevance of autophagy with insights into transcriptional and epigenetic control

### Overview of cellular autophagy

Autophagy is an evolutionarily conserved catabolic process by which cells degrade and recycle cytoplasmic components through lysosomal pathways. It serves as a key mechanism for maintaining cellular homeostasis, especially under stress conditions such as nutrient deprivation, hypoxia, and protein aggregation.^1^ Depending on the cargo, autophagy can be nonselective, engulfing bulk cytoplasm, or selective, targeting specific substrates such as damaged organelles (such as mitophagy, pexophagy, lysophagy, and ER-phagy).^2^

In addition to macroautophagy, which is the primary focus of this review, cells employ two other forms of autophagy: microautophagy and chaperone-mediated autophagy (CMA). Microautophagy involves direct invagination of the lysosomal membrane to engulf cytosolic components,^3^ whereas CMA selectively degrades proteins harboring KFERQ-like motifs through recognition by the chaperone Hsc70 and subsequent translocation through LAMP2A.^4^ Although less extensively studied than macroautophagy, both microautophagy and CMA are tightly regulated and contribute to proteostasis, metabolism, and stress adaptation.^4,5^ Notably, emerging evidence suggests that transcriptional and epigenetic mechanisms also influence these processes, thus supporting their consideration in the broader regulatory landscape of autophagy.^5,6^

**Fig. 1:**
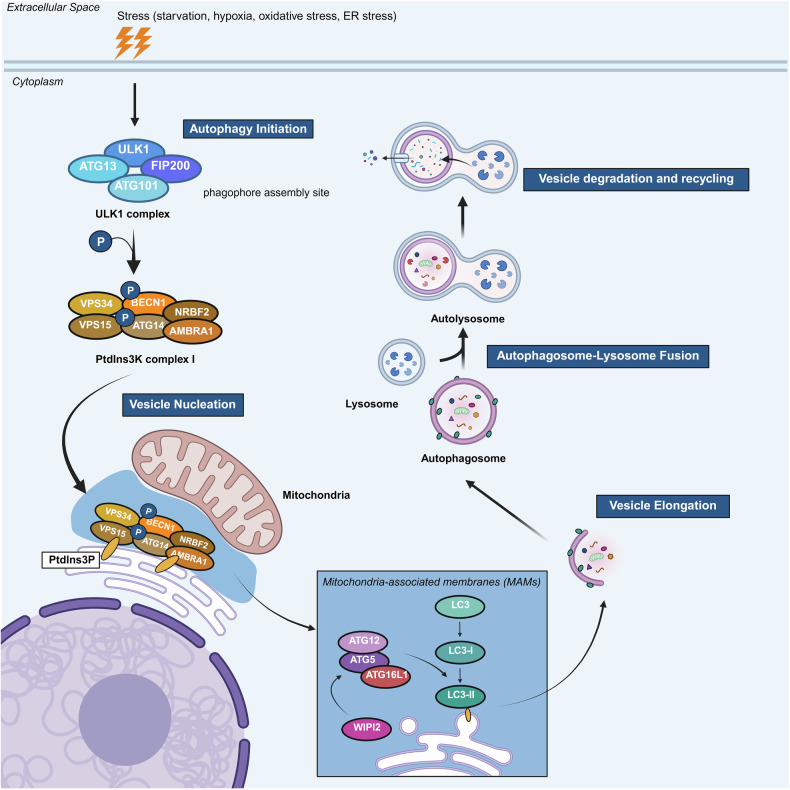
Overview of the autophagy pathway: From initiation to vesicle degradation. The diagram illustrates the autophagy process, which is initiated by cellular stress and involves activation of the ULK1 complex, followed by vesicle nucleation, which is mediated by phosphatidylinositol 3-kinase complex I, and subsequent autophagosome formation. The autophagosome then fuses with lysosomes to form autolysosomes, facilitating the degradation of intracellular components. Key proteins and complexes involved at each stage are depicted. The figure was created with BioRender.com

Autophagy is broadly divided into five stages: initiation, nucleation, elongation, autophagosome–lysosome fusion, and cargo degradation/recycling (Fig. 1). These events are tightly regulated by autophagy-related proteins, with upstream regulation mediated by nutrient-sensing kinases such as mTOR and AMPK. Key molecular complexes include the ULK1 complex for initiation, class III PI3K complex I (VPS34–Beclin 1) for nucleation, and two ubiquitin-like conjugation systems for membrane elongation involving ATG12–ATG5–ATG16L1 and LC3-II. Upon maturation, autophagosomes fuse with lysosomes to form autolysosomes, where hydrolytic enzymes degrade sequestered material, releasing metabolites into the cytosol for reuse. As these mechanisms are well established, readers are referred to comprehensive reviews for detailed molecular descriptions of autophagy pathways.^7,8^

### Diseases associated with autophagy dysregulation

**Fig. 2:**
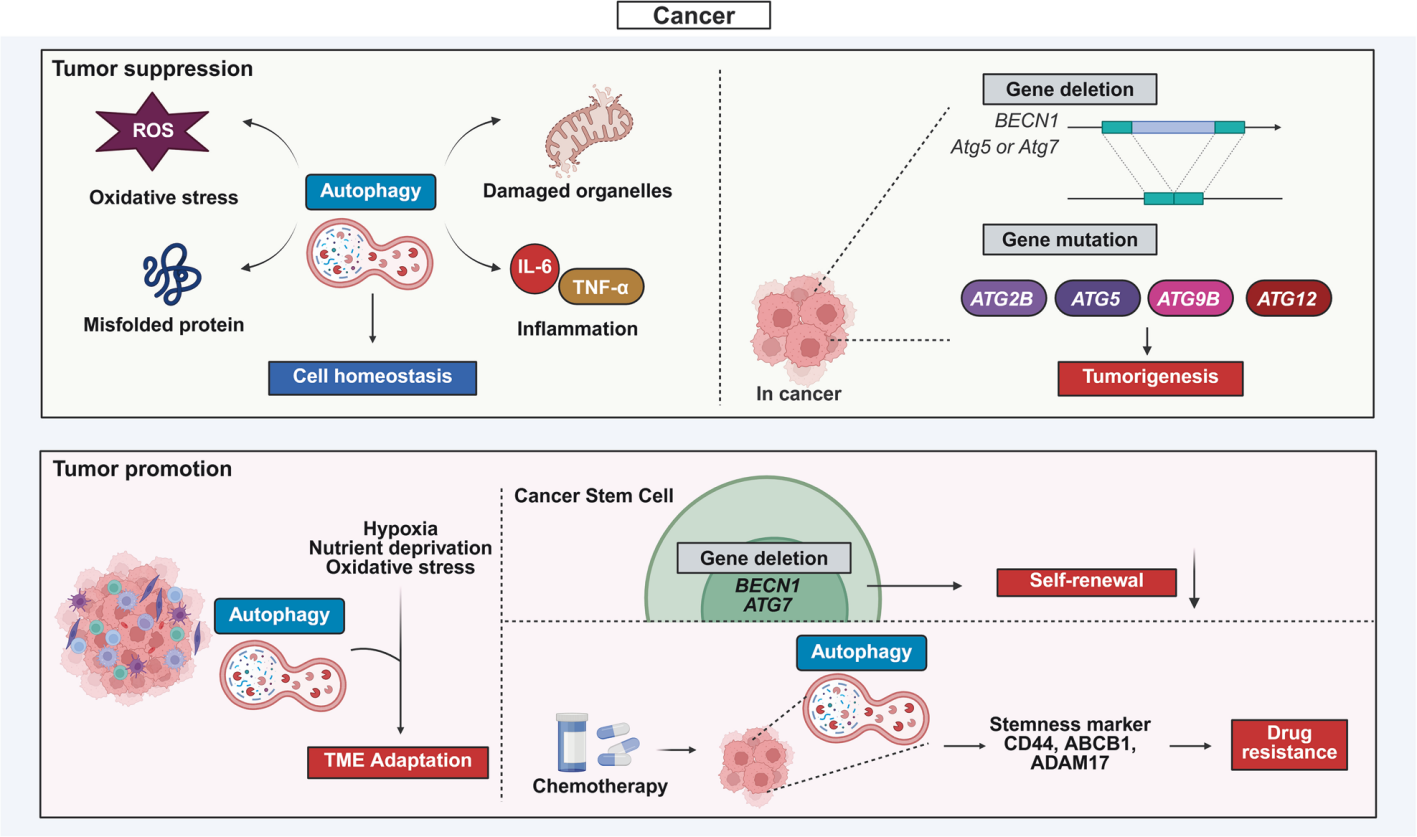
Dual roles of autophagy in cancer: tumor suppression and tumor promotion. The illustration depicts the context-dependent functions of autophagy during cancer initiation and progression. In early carcinogenesis, autophagy acts as a tumor-suppressive mechanism by removing damaged organelles and misfolded proteins, reducing oxidative stress, and limiting inflammation and cellular homeostasis. Loss or mutation of core autophagy genes such as *BECN1, ATG2B, ATG5, ATG9B*, and *ATG12* promotes malignant transformation. In contrast, in advanced or established tumors, autophagy is increased to adapt to microenvironmental stressors such as hypoxia, nutrient deprivation, and ER stress, thereby maintaining mitochondrial function, redox balance, and cancer stem cell (CSC) survival. Moreover, autophagy contributes to chemoresistance through the induction of stemness-related molecules such as CD44, ABCB1, and ADAM17. Together, these dual and stage-specific roles highlight the paradoxical impact of autophagy on tumor biology. The figure was created with BioRender.com

**Fig. 3:**
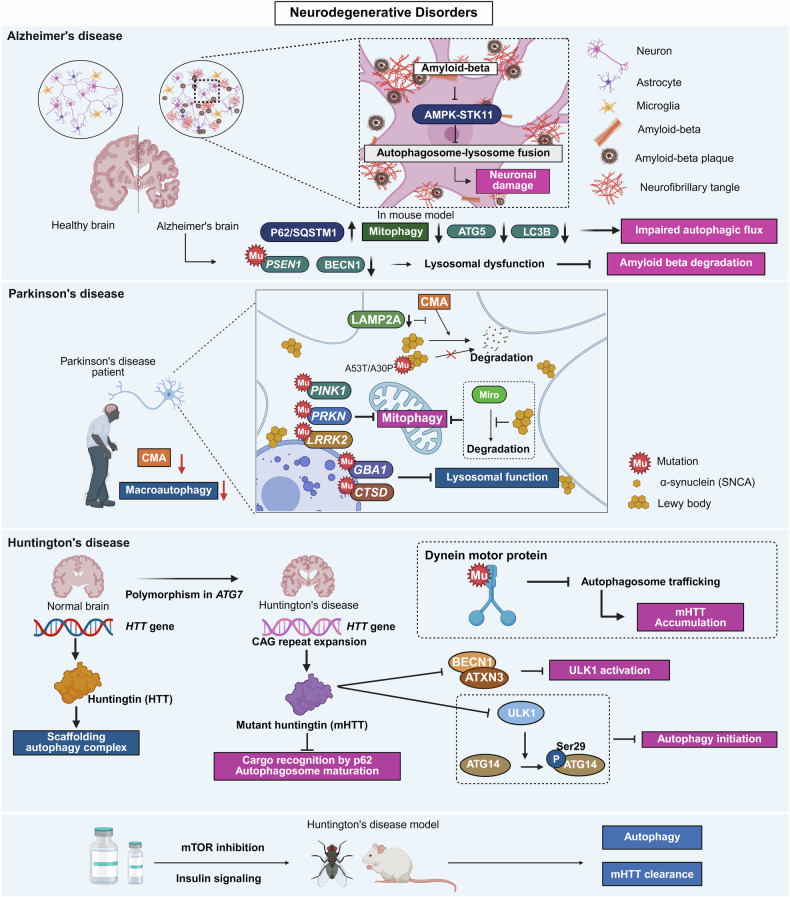
Autophagy dysfunction in neurodegenerative disorders: Alzheimer’s disease (AD), Parkinson’s disease (PD), and Huntington’s disease (HD). This figure illustrates how impaired autophagy contributes to the pathogenesis of major neurodegenerative diseases. (Top) AD: In AD, autophagic flux is disrupted because of impaired autophagosome–lysosome fusion, which is partly mediated by dysfunctional AMPK–STK11 signaling. The accumulation of autophagy substrates such as p62/SQSTM1, decreased expression of key autophagy components (*ATG5, LC3B*, and *BECN1*), and lysosomal dysfunction resulting from *PSEN1* mutations collectively impair amyloid-β (Aβ) degradation and calcium homeostasis, promoting neuronal injury and cognitive decline. (Middle) PD is characterized by α-synuclein (SNCA) accumulation and Lewy body formation, and PD involves defects in both chaperone-mediated autophagy (CMA) and mitophagy. Dysfunction of LAMP2A, as well as mutations in *PRKN, PINK1*, and *LRRK2*, disrupt SNCA degradation and mitochondrial quality control. Mutations in lysosomal enzymes (*GBA1* and *CTSD*) further compromise degradation capacity, whereas SNCA aggregates block Miro degradation, delaying mitophagic clearance. (Bottom) HD: Expanded CAG repeats in the HTT gene generate mutant huntingtin (mHTT), which impairs BECN1/ATXN3 interactions and ATG14 phosphorylation, thereby suppressing ULK1-mediated autophagy initiation and autophagosome maturation. Polymorphisms in ATG7 and dynein motor mutations exacerbate defects in autophagosome trafficking, leading to mHTT accumulation. Pharmacological inhibition of mTOR or modulation of insulin signaling restores autophagic flux and enhances mHTT clearance in preclinical models. The figure was created with BioRender.com

**Fig. 4:**
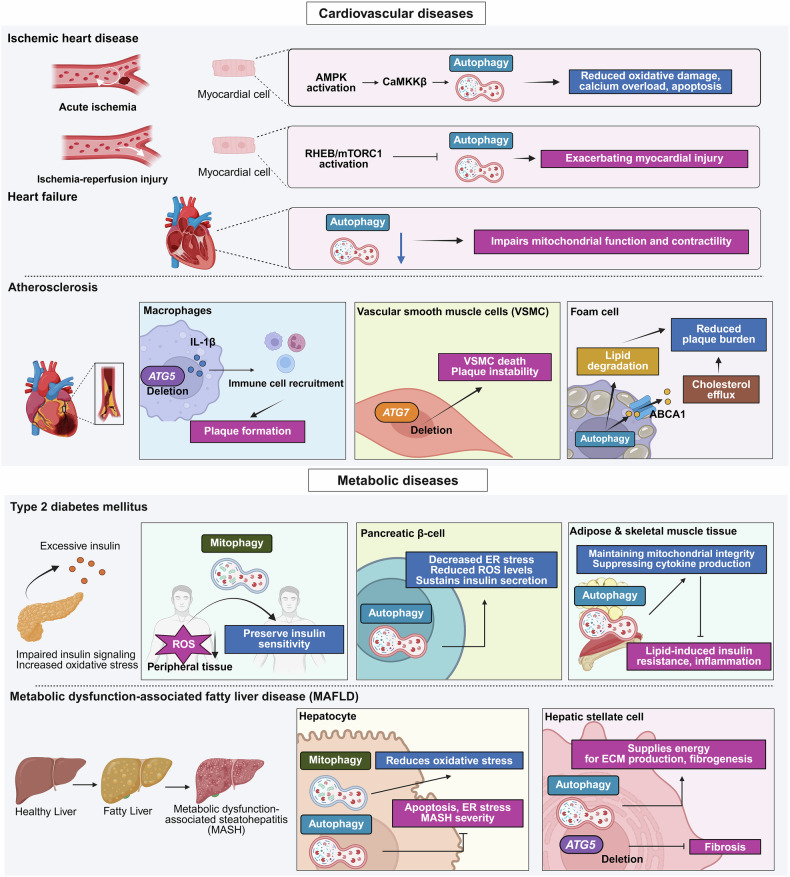
Context-dependent roles of autophagy in cardiovascular and metabolic diseases. This figure summarizes the multifaceted and stage-dependent functions of autophagy across cardiovascular and metabolic disorders, highlighting its dual protective or pathogenic effects depending on the cell type, stress signals, and disease context. (Top) Cardiovascular diseases (CVDs): In ischemic heart disease, autophagy is transiently activated through AMPK–CaMKKβ signaling during acute ischemia to reduce oxidative stress, calcium overload, and apoptosis. However, excessive or persistent activation during reperfusion, coupled with RHEB/mTORC1-mediated inhibition, exacerbates myocardial injury. In heart failure, reduced autophagy impairs mitochondrial function and compromises cardiac contractility. In atherosclerosis, autophagy plays divergent roles: macrophage-specific *ATG5* deficiency increases IL-1β secretion and immune recruitment, accelerating plaque formation, and *ATG7* loss in vascular smooth muscle cells (VSMCs) induces apoptosis and plaque instability. Conversely, autophagy in foam cells enhances ABCA1-mediated cholesterol efflux, reducing lipid accumulation and plaque growth. (Middle) Type 2 diabetes mellitus (T2DM): Autophagy contributes to glucose homeostasis through tissue-specific functions. In peripheral tissues, mitophagy eliminates damaged mitochondria, reducing ROS and preserving insulin sensitivity. In pancreatic β-cells, autophagy mitigates ER stress and sustains insulin secretion, while its deficiency promotes oxidative damage and β-cell loss. In adipose and skeletal muscle, autophagy maintains mitochondrial health and suppresses inflammation, thereby protecting against lipid-induced insulin resistance. (Bottom) Metabolic dysfunction-associated fatty liver disease (MAFLD): In hepatocytes, mitophagy defends against oxidative stress, and autophagy alleviates apoptosis and ER stress, thereby restricting the progression from steatosis to metabolic dysfunction–associated steatohepatitis (MASH). Conversely, in hepatic stellate cells (HSCs), autophagy provides metabolic substrates for extracellular matrix (ECM) synthesis and fibrogenesis; *ATG5* deletion in HSCs attenuates fibrosis, highlighting the cell type–specific dual roles of autophagy in liver disease. The figure was created with BioRender.com

**Fig. 5:**
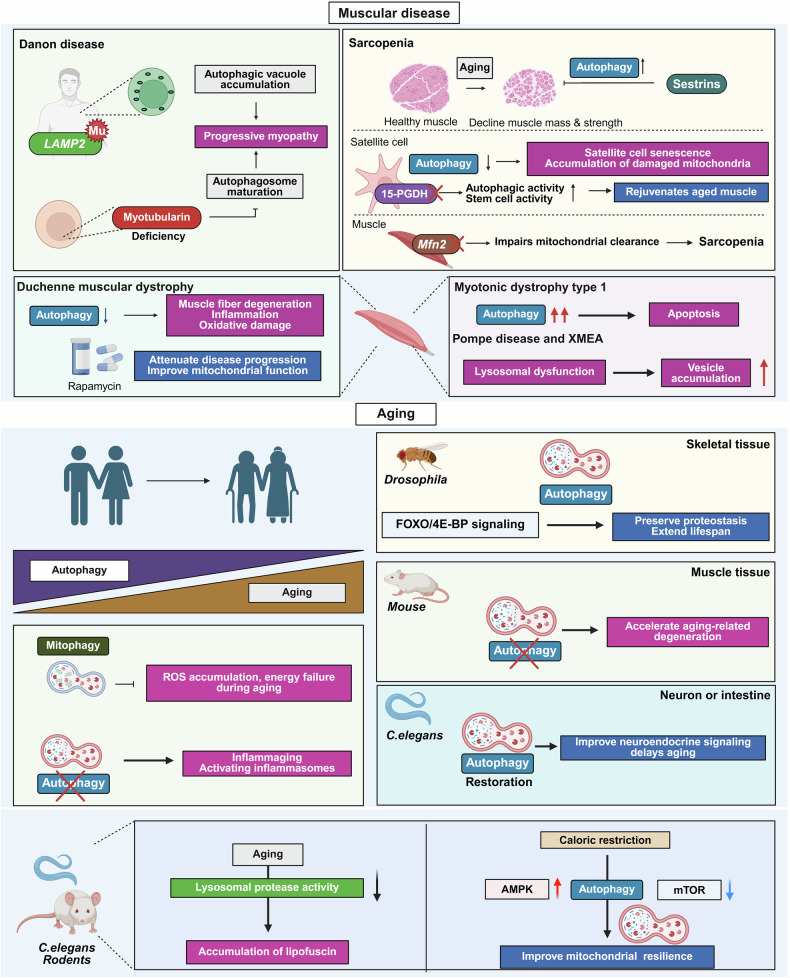
Autophagy regulation in muscular diseases and aging. This figure illustrates the multifaceted roles of autophagy in muscle homeostasis, muscular diseases, and aging-related functional decline. (Top) Muscular diseases: In Danon disease, mutations in *LAMP2* impair autophagosome–lysosome fusion, leading to autophagic vacuole accumulation and progressive myopathy. Similarly, myotubularin deficiency disrupts autophagosome maturation. In sarcopenia, the aging-associated decline in autophagy contributes to satellite cell senescence, mitochondrial dysfunction, and muscle atrophy. Sestrins prevent atrophy by sustaining autophagy, and pharmacologic inhibition of 15-PGDH enhances autophagy and stem cell renewal, thereby rejuvenating aged muscle. Loss of *Mfn2* impairs mitochondrial clearance and exacerbates sarcopenia. In Duchenne muscular dystrophy (DMD), suppressed autophagy accelerates degeneration, inflammation, and oxidative stress, whereas rapamycin-induced autophagy improves mitochondrial function and slows disease progression. In contrast, in myotonic dystrophy type 1, excessive autophagy promotes apoptosis and signaling disruption. In Pompe disease and X-linked myopathy with excessive autophagy (XMEA), lysosomal dysfunction blocks degradation, causing autophagic vesicle accumulation and myofiber degeneration. (Bottom) Aging: Autophagy progressively decreases with age across multiple tissues. In *Drosophila*, FOXO/4E-BP-mediated autophagy maintains proteostasis and extends lifespan, whereas in mice, loss of autophagy accelerates muscle degeneration and neuromuscular junction deterioration. In *C. elegans*, restoring autophagy in neurons or intestines ameliorates aging-related decline. Age-associated lysosomal dysfunction—marked by reduced protease activity and macromolecule accumulation—further disrupts proteostasis. Caloric restriction enhances autophagy through AMPK activation and mTOR inhibition, improving mitochondrial function and extending healthspan. The figure was created with BioRender.com

Autophagy dysregulation contributes to a wide range of diseases, ranging from cancer to aging. This section outlines the disease-specific roles of autophagy and is organized into six subchapters for clarity (Figs. 2–5).

#### Autophagy in cancer

Autophagy plays dual roles in cancer depending on the tumor type, genetic alterations, and disease stage.^9^ It suppresses malignant transformation in the early phases through quality control mechanisms but supports tumor survival and adaptation in later stages (Fig. 2). This dynamic is evident in therapy resistance and metastasis.

##### Tumor suppression in early carcinogenesis

In early tumorigenesis, autophagy preserves cellular homeostasis by limiting oxidative stress, clearing damaged organelles, and regulating inflammation.^10^ *Beclin 1* (*BECN1*), the first identified tumor-suppressive autophagy gene, is frequently lost in ovarian (approximately 75%),^11^ breast (approximately 50%),^12^ and prostate (approximately 40%) cancers.^13^ Although BECN1 promotes autophagy in yeast and human cancer cells, loss or haploinsufficiency of BECN1 accelerates tumor formation in mouse models.^14,15^ Similarly, liver-specific *Atg5* or *Atg7* deletion in mice leads to spontaneous liver tumors, highlighting the role of autophagy in suppressing hepatocellular carcinoma.^16^ Additional genetic alterations, including frameshift mutations in *ATG2B, ATG5, ATG9B*, and *ATG12*^17^ as well as cancer-specific splicing variants of *ATG5*, further impair autophagy and contribute to tumor initiation.^18^

##### Autophagy as a tumor-promoting mechanism

In established tumors, autophagy is upregulated to cope with hypoxia, nutrient scarcity, and oxidative stress.^9,10^ In pancreatic cancer, autophagy maintains mitochondrial function and redox balance, and genetic or pharmacological inhibition of autophagy markedly suppresses tumor growth and reduces tumor burden.^19,20^ In addition, autophagy supports cancer stem cell maintenance, as shown by the requirement of BECN1 and ATG7 in breast cancer.^21,22^ Autophagy also confers protection against chemotherapy. In ovarian cancer, the inhibition of autophagy via the use of chloroquine or *ATG7* silencing effectively reversed chemoresistance.^23^ In oral squamous cell carcinoma (OSCC), autophagy promotes resistance through the upregulation of CD44, ABCB1, and ADAM17.^24^ Conversely, the inhibition of autophagy enhances oxaliplatin sensitivity in colorectal cancer (CRC).^25^

##### Autophagy in cancer metastasis

Autophagy plays stage-specific roles in metastasis by promoting epithelial‒mesenchymal transition (EMT), migration, and survival under conditions of anoikis and oxidative stress and by increasing the secretion of proinvasive factors in KRAS-transformed cells.^26^ In solid tumors such as breast and prostate cancers, as well as melanoma, disseminated tumor cells often enter dormancy in distant organs. Notably, autophagy sustains dormancy by preserving metabolic stability.^27^ Conversely, autophagy can suppress metastatic colonization by preventing dormant cell reactivation.^28^ These dual roles highlight the need for context-specific strategies when targeting autophagy in metastasis.

##### Therapeutic implications

The context-dependent nature of autophagy provides both therapeutic opportunities and challenges. In early or genetically predisposed cancers, its activation can suppress transformation through detoxification and genome stabilization, whereas autophagy-addicted tumors, particularly those driven by KRAS, may benefit from inhibition.^9,29^ Preclinical studies have shown that dual inhibition of ULK1/2 and KRAS^G12C^ reduces tumor growth in KRAS-driven lung cancer.^30^ A phase II trial in which binimetinib was combined with hydroxychloroquine demonstrated benefits in patients with KRAS-mutant NSCLC.^31^ Autophagy modulation also influences therapy resistance and relapse; hydroxychloroquine is under clinical evaluation with chemotherapy, whereas transient activation may sensitize dormant tumor cells to immune or cytotoxic clearance. Optimal strategies require patient stratification by genetic context, tumor stage, metabolic status, and autophagy activity.

#### Autophagy in neurodegenerative diseases

Autophagy is essential for neuronal homeostasis, especially in postmitotic neurons, which cannot dilute toxic protein aggregates through division.^32^ By failing to clear misfolded proteins and damaged mitochondria, impaired autophagy contributes to neurodegenerative diseases. Here, we summarize autophagy dysfunction in AD, PD, and Huntington’s disease (HD), focusing on mechanisms and therapeutic relevance (Fig. 3).

##### AD

AD is characterized by memory loss, extracellular Aβ plaques, and intracellular tau tangles.^33^ Autophagy promotes tau clearance, whereas its inhibition accelerates toxicity.^34^ Elevated p62/SQSTM1 and other substrates indicate impaired flux,^35^ and Aβ disrupts autophagosome–lysosome fusion through AMPK–STK11 signaling.^36^ In mouse models, ATG5, LC3B, and mitophagy are reduced in hippocampal neurons,^37^ and early BECN1 loss aggravates pathology, whereas restoration improves Aβ clearance and cognition.^38^ In addition, *PSEN1* mutations impair lysosomal Aβ degradation and calcium balance.^39^ These findings reveal that autophagic failure is central to AD pathogenesis.

##### PD

PD is characterized by dopaminergic neuron loss and α-synuclein (SNCA) accumulation in Lewy bodies.^40^ CMA normally clears SNCA, but mutations (A53T/A30P) and reduced LAMP2A impair this process.^41–43^ Lysosomal dysfunction resulting from *GBA1* or *CTSD* mutations^44^ and mitophagy defects resulting from *PRKN, PINK1*, or *LRRK2* mutations further contribute to pathology.^45,46^ SNCA aggregates also block Miro degradation, delaying mitophagy,^47^ whereas Rab1A overexpression^48^ or Sirtuin-2 inhibition^49^ can restore flux. Overall, both CMA and macroautophagy are severely compromised in PD.

##### HD

HD results from CAG repeat expansion in the *HTT* gene, leading to the production of mutant huntingtin (mHTT).^50^ Wild-type HTT supports selective autophagy, whereas mHTT disrupts cargo recognition, autophagosome maturation, and interactions with BECN1/ATXN3, thereby reducing ULK1 activation.^51–53^ Expanded PolyQ tracts further impair ATG14 phosphorylation and autophagy initiation,^54^ and *ATG7* polymorphisms accelerate disease onset.^55^ Dynein motor mutations hinder autophagosome trafficking, thus causing mHTT accumulation.^56^ Conversely, mTOR inhibition or insulin signaling enhances autophagy and mHTT clearance in animal models.^57,58^

In summary, autophagy dysfunction is a shared mechanism across AD, PD, and HD. Deficits in protein clearance, mitophagy, and lysosomal function contribute to neurodegeneration. Therapeutic restoration of autophagy could provide disease-modifying potential across these disorders.

#### Autophagy in cardiovascular diseases

Cardiovascular diseases (CVDs) are the leading cause of death worldwide. Cardiomyocytes depend on autophagy and mitophagy to maintain homeostasis and resist stress-induced injury owing to their limited regenerative capacity.^59^ Dysregulated autophagy contributes to heart failure, ischemic injury, and vascular pathologies. This section outlines its multifaceted roles in cardiac protection, damage, and vascular remodeling (Fig. 4).

##### Basal and stress-induced autophagy in the heart

Basal autophagy maintains cardiomyocyte proteostasis and energy balance by removing damaged organelles and misfolded proteins.^60^ Under hemodynamic stress, autophagy is increased for cardioprotection, whereas loss of *Atg5* causes spontaneous cardiomyopathy and stress vulnerability.^61^ Mutations in *VMA21* disrupt lysosomal acidification, leading to vacuole accumulation in X-linked vacuolar cardiomyopathy.^62^ Diseased hearts frequently exhibit increased numbers of autophagosomes, reflecting either enhanced induction or impaired clearance.^63^

##### Autophagy in ischemic heart disease and heart failure

Autophagy is cytoprotective during acute ischemia by reducing oxidative stress and apoptosis, with CaMKKβ–AMPK signaling playing a key role.^64^ However, persistent activation during reperfusion exacerbates cell death, as AMPK promotes autophagy, whereas RHEB/mTORC1 suppresses it, aggravating myocardial injury.^65,66^ In heart failure, reduced autophagy impairs mitochondria and contractility, whereas early activation may be protective. However, excessive autophagy in advanced stages contributes to cardiomyocyte death.^59,67^

##### Mitophagy and mitochondrial quality control

Mitophagy is essential for mitochondrial integrity in cardiomyocytes. Loss of *PGAM5, DRP1*, or *PRKN* disrupts mitochondrial clearance and increases infarct size and apoptosis after ischemia.^68^ Similarly, deletion of *Atg7* or *LAMP2* leads to cardiomyopathy with mitochondrial defects,^67^ thus emphasizing the vital link between autophagy and mitochondrial health.

##### Autophagy in atherosclerosis and vascular remodeling

In vascular tissues, autophagy regulates immune activation and lipid clearance. *Atg5* deletion in macrophages accelerates plaque formation through interleukin (IL)-1β–mediated immune recruitment,^69^ whereas *Atg7* loss in vascular smooth muscle cells (VSMCs) increases apoptosis and plaque instability.^70^ In addition, autophagy degrades lipid droplets and promotes ABCA1-mediated cholesterol efflux from foam cells, thereby limiting the plaque burden.^71^ Overall, autophagy plays context-specific roles in cardiovascular disease, supporting vascular and cardiac homeostasis but contributing to pathology when dysregulated.

#### Autophagy in metabolic diseases

Autophagy is regulated by nutrient-sensing kinases such as mTOR and AMPK and maintains metabolic balance by recycling intracellular components. Because of the breadth of this topic, we focus on two representative conditions: type 2 diabetes mellitus (T2DM) and metabolic-associated fatty liver disease (MAFLD) (Fig. 4).

##### Autophagy and glucose homeostasis

In T2DM, impaired insulin signaling and oxidative stress promote disease progression. Autophagy, particularly mitophagy, preserves insulin sensitivity by removing damaged mitochondria and reducing reactive oxygen species (ROS).^72^ In pancreatic β-cells, it alleviates ER stress and promotes insulin secretion, whereas its deficiency accelerates oxidative damage and β-cell failure.^73,74^ In adipose tissue and muscle, autophagy maintains mitochondrial health and suppresses inflammation, protecting against lipid-induced insulin resistance.^75^ Overall, autophagy is essential for glucose homeostasis across metabolic tissues.

##### Hepatic autophagy and MAFLD progression

Hepatic autophagy regulates lipid, protein, and glycogen turnover in response to nutrient status. In obesity, impaired autophagy promotes lipotoxicity and drives the progression from steatosis to metabolic dysfunction-associated steatohepatitis (MASH).^76,77^ BNIP3-mediated mitophagy protects hepatocytes from oxidative stress, whereas autophagy reduces apoptosis and ER stress, thereby limiting MASH severity.^76,77^ Conversely, in hepatic stellate cells, autophagy fuels fibrogenesis, and *ATG5* deletion reduces fibrosis, revealing its context-dependent role.^78^

##### CMA and metabolic regulation

CMA regulates lipid metabolism by degrading perilipins in hepatocytes, thereby promoting lipophagy and triglyceride breakdown.^79^ Its impairment disrupts glycogenolysis and lipid turnover, causing hepatic steatosis and metabolic imbalance.^80^ Overall, autophagy influences metabolic health in a tissue- and stage-specific manner, and therapeutic targeting requires precision on the basis of the disease context.

#### Autophagy in muscular diseases

Autophagy is crucial for skeletal muscle homeostasis because it removes damaged organelles and protein aggregates, especially in postmitotic muscle fibers. Its dysregulation contributes to myopathies, sarcopenia, and muscular dystrophies. This section outlines the diverse roles of autophagy in muscle physiology and pathology (Fig. 5).

##### Autophagy-related myopathies

Defective autophagy contributes to multiple myopathies. In Danon disease, *LAMP2* mutations impair autophagosome–lysosome fusion, causing vacuole accumulation and muscle degeneration.^81,82^ Myotubularin deficiency disrupts phosphatidylinositol metabolism and blocks autophagosome maturation, leading to progressive weakness.^83^ In collagen VI-related dystrophies, reduced BECN1 hampers autophagy initiation and myofiber integrity, whereas autophagy restoration rescues the phenotype.^84^ Similarly, chloroquine-induced myopathy impairs lysosomal degradation, resulting in the formation of rimmed vacuoles.^85^

##### Sarcopenia and muscle aging

Autophagy preserves muscle function during aging, and its impairment in sarcopenia leads to mitochondrial accumulation and satellite cell senescence.^86^ The inhibition of 15-PGDH rejuvenates aged muscle by increasing autophagy and stem cell activity,^87^ whereas Sestrins integrate stress and nutrient signals to prevent atrophy.^88^ Mfn2-mediated mitochondrial fusion supports mitophagy, and its loss impairs clearance, contributing to sarcopenia.^89^ Enhancing autophagic flux may assist in counteracting age-related muscle decline.

##### Autophagy in muscular dystrophies

In Duchenne muscular dystrophy (DMD), defective autophagy aggravates degeneration, inflammation, and oxidative stress, whereas rapamycin-induced autophagy improves mitochondrial function and slows progression.^90^ In myotonic dystrophy type 1, excessive autophagy promotes apoptosis and signaling disruption.^91^ In Pompe disease and XMEA, lysosomal dysfunction impairs lysosomal degradation and causes vesicle buildup, revealing the importance of lysosomal integrity.^92^ Overall, autophagy acts as a double-edged sword in muscle and is beneficial when balanced but harmful when deficient or excessive.

#### Autophagy in aging

Aging is characterized by gradual functional decline at the cellular and systemic levels. Autophagy supports healthy aging by regulating proteostasis, organelle turnover, and immune balance. However, autophagy activity decreases with age, promoting the buildup of damaged components and increasing vulnerability to age-related diseases such as neurodegeneration, cancer, and metabolic disorders (Fig. 5).^93^

##### Decline in autophagy with age

Studies in yeast, worms, flies, and mice have shown that autophagy-related genes (ATGs) are essential for lifespan extension under caloric restriction (CR) or reduced insulin signaling.^93^ Aging is associated with a marked decline in autophagy, characterized by the downregulation of ATGs such as *Atg2, Atg8a*, and *bchs* in *Drosophila* models, as well as impaired autophagy in the neuronal cells of aged mice.^94,95^ Mitophagy decreases in aged cells; however, its pharmacological activation can counteract senescence- and aging-related cellular dysfunction.^96^

##### Tissue-specific roles of autophagy in aging

The role of autophagy varies by tissue. In skeletal muscle, FOXO/4E-BP-driven autophagy preserves proteostasis and extends lifespan in flies.^97^ In mice, autophagy deficiency in muscle accelerates age-related decline and neuromuscular junction deterioration.^98^ In *C. elegans*, restoring neuronal or intestinal autophagy improves neuroendocrine signaling and delays aging.^99,100^

##### Mitophagy and inflammaging

Mitophagy is critical during aging to prevent ROS accumulation and energy failure, and its decline exacerbates oxidative stress and metabolic dysfunction.^101^ In addition, impaired autophagy drives inflammaging by sustaining proinflammatory immune cells and activating inflammasomes.^102^ Through metabolic signaling, autophagy regulates macrophage function, with mTOR suppressing and AMPK promoting autophagy and anti-inflammatory phenotypes.^103^

##### Lysosomal dysfunction and longevity interventions

Aging is associated with lysosomal decline, including reduced protease activity and lipofuscin accumulation in *C. elegans* and rodents, impairing proteostasis.^104^ CR extends lifespan by inducing autophagy through AMPK activation and mTOR inhibition, enhancing mitochondrial resilience.^105^ Genetic or pharmacologic enhancement of autophagy mimics the benefits of CR, thereby revealing its therapeutic potential for healthy aging. In summary, a decrease in autophagy contributes to age-related dysfunction, whereas its restoration may delay aging and reduce the chronic disease burden.

### Regulatory mechanisms of autophagy

#### Autophagy regulation by nutrient-sensing kinases

**Fig. 6:**
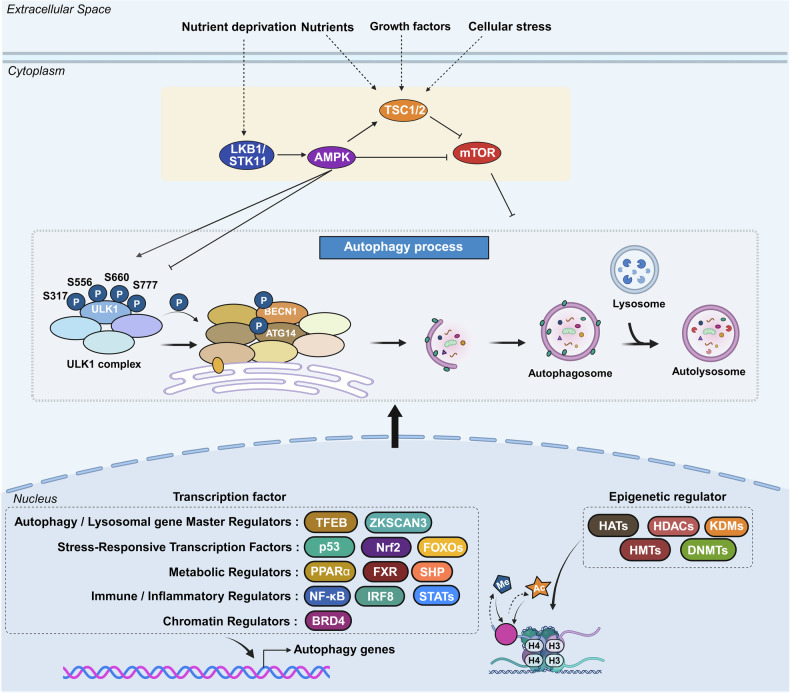
Regulation of autophagy by nutrient-sensing kinases, transcriptional, and epigenetic mechanisms in response to stress and nutrients. Autophagy is regulated by a coordinated network of nutrient-sensing kinases, transcription factors, and epigenetic mechanisms in response to metabolic and environmental cues. In the cytoplasm, nutrient-sensing kinases dynamically modulate autophagy in accordance with the cellular energy status. AMPK, which is activated by LKB1/STK11 under nutrient deprivation conditions, promotes autophagy through multiple mechanisms: phosphorylating and activating ULK1 and inhibiting mTORC1 through the TSC1/2 complex. Conversely, mTOR suppresses autophagy in response to nutrients, growth factors, and stress signals. Notably, AMPK can also negatively regulate autophagy under specific metabolic stresses—such as glucose starvation or mitochondrial dysfunction—by inhibiting ULK1-dependent autophagosome formation or disrupting lysosomal acidification. In the nucleus, transcription factors orchestrate ATG expression, whereas epigenetic modifiers, including histone acetyltransferases, deacetylases, and lysine demethylases, alter chromatin accessibility to fine-tune autophagy transcription. Collectively, these kinase, transcriptional, and epigenetic circuits ensure that autophagy is precisely adjusted to meet metabolic and stress demands. The figure was created with BioRender.com

Cells regulate autophagy through various signaling pathways in response to organelle damage, hypoxia, inflammation, growth factors, hormones, nutrients, and energy stress.^106^ Changes in the cellular energy status, particularly fluctuations in glucose and amino acid availability, serve as powerful regulators of the autophagic process.^107^ These nutrient fluctuations are detected by upstream nutrient-sensing kinases, which play crucial roles in controlling autophagy from initiation to termination (Fig. 6).

Among these regulators, mTOR is the central signaling molecule that governs autophagy. mTOR, a highly conserved serine/threonine kinase, integrates energy levels, growth factors, and stress signals to modulate autophagy.^108^ It forms two distinct complexes, mTOR complex 1 (mTORC1) and mTOR complex 2 (mTORC2), with mTORC1 being the primary regulator of autophagy. Under nutrient-rich conditions, mTORC1 is activated, leading to the inhibition of the ULK1 complex and the suppression of autophagy, thereby promoting cell growth and proliferation. Conversely, under nutrient deprivation or stress, mTORC1 is inactivated, leading to autophagy induction and a reduction in cellular energy demands, thus enabling cells to adapt and survive under adverse conditions.^109^ The TSC1/2 protein complex acts as a negative regulator of mTORC1, further emphasizing its role in controlling autophagy.^110^

In addition, AMPK is a key nutrient-sensing regulator that induces autophagy in response to nutrient deficiency.^111^ AMPK senses intracellular AMP levels and, when activated, regulates cell metabolism, including autophagy, cell growth, protein synthesis, glycolysis, and fatty acid oxidation. AMPK promotes autophagy through multiple mechanisms: it directly phosphorylates the TSC1/2 complex, inhibiting mTORC1; it enhances ULK1 phosphorylation, initiating autophagy; and it phosphorylates Raptor, an mTORC1 component, thereby reducing mTORC1 kinase activity.^109,112,113^ Recent studies have shown that AMPK exerts dual, context-dependent effects on ULK1 and autophagy. AMPK generally activates ULK1 to promote autophagy under energy stress; however, emerging evidence has revealed that it can also suppress autophagy initiation under specific metabolic conditions. In particular, during glucose starvation or severe energy crises such as mitochondrial dysfunction, AMPK has been shown to negatively regulate ULK1 activity, either by inhibiting ULK1-dependent autophagosome formation or by disrupting lysosomal acidification, thereby attenuating autophagy initiation.^114,115^

In addition, the tumor suppressor kinase LKB1/STK11 activates AMPK through phosphorylation under nutrient deprivation conditions.^116^ Under these conditions, AMPK activates c-Jun N-terminal protein kinase, which phosphorylates Bcl-2, stabilizing the Beclin-1-VPS34 complex, a crucial step in autophagy induction.^117^ These findings highlight the interplay between intracellular kinases, particularly mTOR and AMPK, which contrastingly regulate autophagy depending on the cellular nutrient status. Collectively, these kinases orchestrate a complex regulatory network, ensuring that autophagy responds dynamically to metabolic changes and cellular energy demands.

#### Transcriptional and epigenetic regulation of autophagy

Earlier, autophagy was presumed to be exclusively regulated by cytoplasmic mechanisms, as evidenced by observations that autophagy persisted even in enucleated cells.^118^ Early studies focused primarily on autophagy regulation by nutrient-sensing kinases and posttranslational modifications of autophagy proteins, whereas the potential role of nuclear regulation has remained largely underexplored.^8^

However, in 1999, autophagy induction during nitrogen starvation in yeast was reported to be driven by transcriptional upregulation of the *Apg8p* gene, a homolog of mammalian LC3.^119^ In 2011, a groundbreaking study revealed that transcription factor EB (TFEB) broadly regulates genes involved in autophagy and lysosome biogenesis at the transcriptional level, marking a pivotal shift in the understanding of the control of autophagy in the nucleus.^120^

Research on the transcriptional and epigenetic regulation of autophagy has since advanced rapidly across various fields. Increasing evidence highlights the crucial role of nuclear events in controlling autophagy, revealing their importance in health and disease. This review aims to summarize the latest research findings on autophagy regulation by transcriptional and epigenetic factors (Tables 1 and 2), highlighting their relevance to disease pathogenesis and therapeutic potential.

**Table 1:** Functional classification of transcription factors regulating autophagy

Functional classification	Transcription factor	Autophagy regulation	Mechanism	Dysregulation
Autophagy/lysosomal gene master regulators	TFEB	Activation	Direct binding to CLEAR motifs in the promoters of autophagy and lysosome-related genes	Serum TFEB levels are reduced in individuals with Alzheimer’s disease compared to cognitively unimpaired controls
	ZKSCAN3	Inhibition	Transcriptional repression of autophagy and lysosomal genes	Upregulated in multiple cancers, including colon, prostate, and liver cancers
	E2F1	Activation	Binds directly to ATG promoters through E2F-binding motifs	-
Stress-responsive transcription factors	p53	Activation, Inhibition	Activates ATG transcription in the nucleus; inhibits autophagy in the cytoplasm through nontranscriptional mechanisms	-
	Nrf2	Activation	Binds to ARE motif and induces autophagic genes including p62 and ULK1	Nrf2 expression decreases with progression of intervertebral disc degeneration
	FOXOs	Activation, Inhibition	Stress-induced nuclear translocation of FOXOs activates ATG transcription; interaction between acetylated FOXO1 and ATG7 enhances autophagic flux	-
	ATFs	Activation (ATF4,6), Inhibition (ATF5)	ATF4 binds to *LC3B* and *ULK1* promoters (CREs); ATF5 induces mTOR expression to suppress autophagy; ATF6–C/EBPβ complex promotes *DAPK1* under IFN-γ	-
	HIF-1α	Activation	Stabilized under hypoxia, translocates to the nucleus, and activates autophagy	-
Metabolic regulators	PPARα	Activation	Under fasting, binds to DR1 elements in ATG promoters and induces transcription	PPARα expression declines during acute liver failure
	NR1H4/FXR	Inhibition	Activated by bile acids under feeding conditions; translocates to the nucleus and represses ATG transcription	-
	NR0B2/SHP	Activation (intestine), Inhibition (liver)	In the liver, recruits LSD1 to repress ATG promoters; in the intestine, promotes lipophagy with TFEB following nutrient intake	-
	GATAs	Activation (GATA1) Inhibition (GATA4)	GATA1 activates ATG transcription; GATA4 represses it	-
	C/EBPs	Activation	C/EBPβ activates ATG transcription; C/EBPα forms complexes with Beclin 1 to promote autophagosome nucleation in hepatic stellate cells	C/EBPα expression is reduced in fibrotic liver tissue
	MYC	Activation, Inhibition	Suppresses autophagy by competing with MiT/TFE transcription factors in cancer; Under ER stress, it enhances autophagy through activation of the PERK-eIF2α-ATF4 signaling pathway	-
Immune/inflammatory regulators	NF-κB	Activation, Inhibition	Represses *Bnip3* under basal conditions through competition with E2F1; TCR activation induces *BECN1* through NF-κB	-
	IRF8	Activation	Binds to ISREs in ATG promoters, inducing ATG transcription in response to innate immune signals	-
	STAT	Activation (STAT3), Inhibition (STAT1, STAT3)	Nuclear STAT3 activates ATG transcription; cytoplasmic STAT3 suppresses autophagy by inhibiting PKR and sequestering FOXO; mitochondrial STAT3 suppresses mitophagy through ROS inhibition; STAT1 represses *ULK1* transcription	-
Chromatin regulators	BRD4	Inhibition	Represses autophagy by recruiting G9a methyltransferase to ATG loci	-
	HMGA1	Inhibition Activation(In neurodegenerative disease)	Represses *ULK1* transcription and activates mTOR signaling; suppresses TP53INP1 in bladder cancer; in Parkinson’s disease models, activates CDK5 signaling through miR-103 regulation	-

**Table 2:** Key epigenetic regulators of autophagy

Functional classification	Epigenetic regulator	Autophagy regulation	Mechanism	Dysregulation
Histone acetyltransferase	KAT5/Tip60	Activation	Recruited to ATG promoters and promotes ATG transcription. Acetylates ULK1 and p53, promoting autophagy initiation	-
	hMOF/KAT8	Activation	Regulates H4K16ac at ATG promoters, facilitating transcription	-
	p300	Inhibition	Acetylates autophagy-related proteins, reducing their activity	-
Histone deacetylase	HDAC1	Activation	Removes histone acetylation at ATG promoters, thereby repressing transcription and deacetylates the ATG16L1 protein	-
	HDAC2	Activation, Inhibition	Modulates lysosomal biogenesis and ATG transcription through interaction with the MYC–MiT/TFE axis; deacetylates STX17 to promote autophagosome maturation	-
	HDAC4	Activation, Inhibition (In diabetic nephropathy)	Promotes autophagy by degrading MEKK3 or activating NF-κB-mediated ATG transcription; inhibits autophagy in diabetic nephropathy through STAT1 deacetylation and chromatin compaction at ATG loci	-
	HDAC5	Inhibition	Induces heterochromatin formation and represses ATG transcription; deacetylates TFEB and inhibits its nuclear translocation	-
	HDAC6	Activation, Inhibition	Promotes autophagosome maturation through cortactin deacetylation; enhances degradation of ubiquitinated aggregates by deacetylating p62 (K420/K435) during starvation; activates CMA through Hsp90 (K489) deacetylation	-
	HDAC7	Inhibition	Deacetylates histones to repress ATG transcription; deacetylates TFEB and inhibits its nuclear translocation; modulates the crotonylation-dependent 14-3-3ε–PPM1B pathway	-
	HDAC8	Inhibition	Interacts with RELA to suppress *PRKN* transcription	-
	HDAC9	Inhibition	Deacetylates histones near ATG promoters to repress ATG transcription; deacetylates TFEB and inhibits its nuclear translocation	-
	HDAC10	Activation	Regulates ATG gene expression under stress; direct histone targets remain unclear	-
	SIRT1	Activation	Deacetylates H4K16ac to activate ATG transcription; deacetylates ATG proteins	-
	SIRT2	Activation, Inhibition	Deacetylates FOXO1 to inhibit FOXO1-ATG7 interaction; deacetylates mitochondrial proteins to enhance mitophagy	-
	SIRT6	Activation	Upregulates KLF4 to promote ATG transcription; activates Beclin 1 and AMPK in esophageal cancer; suppresses IGF–Akt signaling and activates ULK1 in bronchial epithelial cells	-
Acetyl-CoA synthetase	ACSS2	Activation (breast cancer, subarachnoid hemorrhage) Inhibition (ovarian cancer, diabetic nephropathy)	Interacts with TFEB to facilitate H3K9ac at ATG promoters; in ovarian cancer, inhibits the SIRT1–ATG5–ATG2B axis; in diabetic nephropathy, promotes H3K9ac at the *raptor* promoter and activates mTORC1	-
Histone, DNA, and RNA methyltransferases	CARM1	Activation	Induces H3R17me to promote ATG transcription and TFEB recruitment at ATG promoters under starvation; methylates Pontin and FOXO3 to enhance ATG expression	-
Histone, DNA, and RNA methyltransferases	G9a	Inhibition, Activation	Under basal or lipotoxic conditions, G9a represses autophagy by enriching H3K9me2 at ATG promoters; promotes H3K9me1 at the *mTOR* promoter, activating *mTOR* expression; Under ischemic stress, G9a deposits H3K9me2 at the *mTOR* promoter and inhibits its expression	-
	EZH2	Inhibition	Deposits H3K27me3 at the promoters of *mTOR*-inhibitory and ATG genes, thereby repressing their expression	-
	DNMT1	Inhibition	Promotes CpG methylation at ATG gene promoters, leading to transcriptional silencing	-
	DNMT3A	Inhibition	Represses transcription of *MAP1LC3* isoforms through promoter DNA methylation	-
	METTL3	Inhibition Activation (some cancers)	Methylates mRNAs of *ATG7, Rubicon, TFEB*, and *ULK1*, regulating their stability to suppress autophagy; methylates *DCP2* and *ATG5* mRNAs to promote autophagy	-
Histone demethylase	LSD1	Inhibition	Demethylates H3K4me2/3 at ATG promoters to suppress gene expression; demethylates LC3B protein, inhibiting autophagy	-
	KDM6B (JMJD3)	Activation	Removes repressive H3K27me3, facilitating ATG transcription; mediates fasting-induced autophagy through FGF21 signaling in the liver	Decreased expression of JMJD3, TFEB, and ATGs observed in MAFLD
	KDM6A/UTX	Activation	Demethylates H3K27me3 to activate ATG transcription. In renal cell carcinoma, UTX interacts with TFE3 and TRIM28 to enhance H3K4me3 levels at ATG promoters	-
	KDM3B	Activation	Removes repressive H3K9me2 marks to promote ATG transcription	-
E3 ubiquitin ligase	RNF20	Inhibition	Catalyzes monoubiquitination of histone H2B (H2Bub1), creating repressive chromatin that inhibits ATG transcription	-
Deubiquitinase	USP44	Activation	Removes H2Bub1 to promote chromatin relaxation and ATG transcription	-
Chromatin remodeling complexes	SWI/SNF & INO80	Activation	Facilitate nucleosome remodeling and chromatin accessibility, promoting ATG gene transcription	-

## Autophagy regulation by transcription factors

### Autophagy/lysosomal gene master regulators

**Fig. 7:**
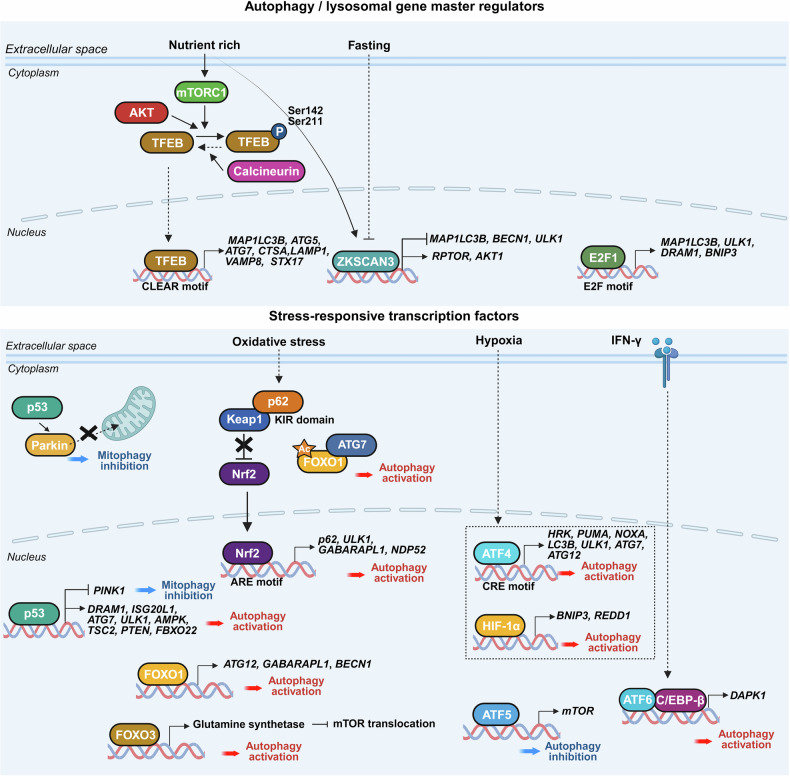
Transcriptional regulation of autophagy by master and stress-responsive transcription factors. This figure illustrates how autophagy is transcriptionally regulated by upstream master regulators and stress-responsive transcription factors in response to environmental stimuli. (Top) Autophagy/lysosomal gene master regulators: TFEB functions as a central activator of autophagy and lysosomal biogenesis by binding to CLEAR motifs in gene promoters. Its activity is regulated by mTORC1 and Akt, which phosphorylate and sequester TFEB in the cytoplasm under nutrient-rich conditions; upon stress or fasting, TFEB translocates into the nucleus and induces the expression of autophagy genes. ZKSCAN3 functions as a transcriptional repressor that antagonizes TFEB to inhibit autophagy and lysosomal gene expression under nutrient-rich conditions. It suppresses autophagy by directly repressing the transcription of core ATGs such as *MAP1LC3B, BECN1*, and *ULK1* and by upregulating negative autophagy regulators, including *RPTOR* and *AKT1*. In contrast, E2F1 promotes the transcription of key ATGs, such as *MAP1LC3B, ULK1, DRAM1*, and *BNIP3*, thereby linking cell cycle regulation with autophagic control. (Bottom) Stress-responsive transcription factors: In response to cellular stress, p53 and FOXO family members positively and negatively regulate autophagy. Nuclear p53 enhances the expression of *ULK1, ATG7*, and *DRAM1*, whereas cytoplasmic p53 inhibits mitophagy by blocking Parkin translocation. FOXO1/3 induce ATG and lysosomal genes, and in the cytoplasm, acetylated FOXO1 interacts with ATG7 to activate autophagy. The Keap1–p62–Nrf2 axis induces the expression of antioxidant and autophagy genes under redox imbalance. Under hypoxia, HIF-1α and ATF4 upregulate *HRK, PUMA, NOXA, LC3B, ULK1, ATG7*, and *ATG12* to promote autophagy and survival. ATF6 and C/EBPβ cooperate under ER stress and immune activation to drive autophagy through *DAPK1* induction. Conversely, ATF5 inhibits autophagy by activating mTOR signaling. The figure was created with BioRender.com

This group comprises central transcriptional regulators that directly coordinate the transcriptional program of autophagy and lysosomal biogenesis. TFEB functions as a master activator of the autophagy-lysosomal pathway by binding to coordinated lysosomal expression and regulation (CLEAR) motifs and promoting the expression of genes involved in autophagosome formation, lysosomal function, and fusion events. In contrast, zinc finger with KRAB and SCAN domains 3 (ZKSCAN3) functions as a global transcriptional repressor, antagonizing TFEB by suppressing ATG, lysosomal, and fusion-related genes under nutrient-rich conditions. E2F1, although traditionally known for its role in cell cycle regulation, also activates a subset of ATGs, such as *LC3, ULK1, DRAM1*, and *BECN1*, and contributes to the regulation of mitochondrial dynamics through *MFN2* (Fig. 7). Collectively, these factors establish a transcriptional framework that governs the core machinery of autophagy initiation, progression, and organelle turnover, integrating stress signals and nutrient availability into precise gene expression responses essential for cellular homeostasis and adaptation.

#### TFEB

TFEB, a member of the bHLH-Zip transcription factor family, is a master regulator of autophagy and lysosomal biogenesis. It exerts its function by directly binding to CLEAR motifs, palindromic E-box-like sequences (GTCACGTGAC), in the promoters of numerous autophagy- and lysosome-related genes.^120,121^ Chromatin immunoprecipitation and transcriptomic analyses have identified TFEB as a transcriptional activator of autophagosome formation genes (*MAP1LC3B, ATG5*, and *ATG7*), lysosomal membrane and hydrolase genes (*LAMP1, CTSA*, and *CTSD*), and fusion-related SNARE components (*STX17* and *VAMP8*).^120,122^ These targets enable TFEB to regulate the early and late stages of autophagy.

TFEB localization and activity are regulated by nutrient and stress conditions. In nutrient-rich environments, mTORC1 phosphorylates TFEB at Ser142 and Ser211, leading to 14-3-3 protein binding, cytoplasmic sequestration, and transcriptional inhibition.^122,123^ Upon nutrient deprivation, oxidative stress, or lysosomal damage, mTORC1 becomes inactive, causing TFEB dephosphorylation and nuclear translocation.^122^ In parallel, lysosomal calcium release through MCOLN1 (TRPML1) activates the phosphatase calcineurin, which also dephosphorylates TFEB, further enhancing its nuclear import and transcriptional activation of CLEAR motif-containing genes.^123,124^ In addition to mTORC1 and calcineurin, Akt kinase modulates TFEB by phosphorylating it to prevent nuclear translocation, whereas Akt inhibition leads to TFEB nuclear accumulation and increased ATG expression.^125^ These pathways reveal that TFEB integrates multiple signaling cues to coordinate autophagy and lysosome biogenesis.

Recent studies have also highlighted the transcriptional-level regulation of TFEB targets. USF2 competes with TFEB for CLEAR motif binding in lysosomal and ATG promoters, reducing the transcriptional output of TFEB.^126^ This interplay provides a mechanism through which metabolic and stress conditions dynamically modulate the autophagy–lysosomal network.

Functionally, TFEB plays diverse roles across tissues. In hepatocytes, TFEB induces lipophagy by upregulating genes involved in lipid droplet recognition and lysosomal lipid degradation, thereby alleviating hepatic steatosis and metabolic syndrome.^120^ In the central nervous system, TFEB promotes the clearance of pathogenic protein aggregates, such as α-synuclein and tau, by inducing lysosomal hydrolases, including *CTSB, GBA*, and *LAMP2*, contributing to neuroprotection in models of PD and frontotemporal dementia.^127^ In skeletal muscle, TFEB supports mitochondrial homeostasis by activating mitophagy-related genes such as *PINK1, PRKN*, and *BNIP3*, which are essential under metabolic or exercise-induced stress.^128^ In immune cells, TFEB enhances microbial clearance and inflammation control through the induction of *IRGM* and *GABARAPL1*.^129^ In kidney epithelial cells, TFEB promotes autophagy‒lysosomal flix and antioxidant gene expression, thereby protecting against oxidative damage and fibrosis.^130^

In summary, TFEB functions as a central transcriptional hub that orchestrates autophagy, lysosomal expansion, and stress adaptation through direct gene regulation and signal-dependent localization. Its control by mTORC1, calcineurin, and Akt integrates nutrient sensing with cellular quality control mechanisms.

#### ZKSCAN3

ZKSCAN3, also known as ZNF306, is a transcriptional repressor that critically regulates autophagy by directly inhibiting the expression of autophagy and lysosomal genes. As a functional antagonist of TFEB, ZKSCAN3 acts as a master repressor that suppresses autophagic activity under nutrient-rich conditions. It binds to the promoters of core ATGs, including *MAP1LC3B, BECN1, ULK1*, and *WIPI1*, repressing their transcription. In addition, it downregulates genes involved in autophagosome‒lysosome fusion and lysosomal function (such as *STX5, SEC22B, ATP6V1A*, and *CTSA*) and enhances the expression of negative autophagy regulators such as *RPTOR* and *AKT1*.^131^

A distinguishing feature of ZKSCAN3 is its nutrient-sensitive localization. Under nutrient-rich conditions, it remains in the nucleus to maintain transcriptional repression. In response to starvation or rapamycin, ZKSCAN3 is exported to the cytoplasm, relieving repression and enabling TFEB to activate CLEAR motif-driven gene expression. This nucleocytoplasmic shuttling mechanism enables rapid transcriptional adaptation to metabolic stress. Loss of ZKSCAN3 leads to increased autophagosome formation and lysosomal biogenesis, whereas ZKSCAN3 overexpression suppresses these effects and inhibits rapamycin-induced autophagy.^131^ Owing to its capacity to repress autophagy at multiple levels, ZKSCAN3 is emerging as a key regulator of cellular catabolism. Its dysregulation is implicated in several cancers, such as colorectal and prostate cancer, where diminished autophagy may promote tumor survival.

#### E2F1

E2F1 is a member of the E2F transcription factor family that is well known for controlling cell cycle progression, DNA replication, and apoptosis. More recently, it has been recognized as a direct regulator of autophagy. E2F1 binds to canonical E2F motifs in the promoters of *MAP1LC3B, ULK1*, and *DRAM1*, activating their transcription and promoting autophagosome formation.^132^ Although it does not bind *ATG5* directly, E2F1 enhances its expression through intermediate regulatory pathways.^132^ Full-length and truncated isoforms of E2F1, including the transactivation domain-deficient E2Ftr, promote autophagy; E2Ftr upregulates *LC3* and *ATG5* expression, indicating that E2F1 also engages noncanonical regulatory mechanisms.^133^

E2F1 plays a significant role in metabolic regulation, particularly in white adipose tissue (WAT). Its deletion increases mitochondrial biogenesis and *UCP-1* expression, reduces autophagy flux, and promotes WAT browning, indicating that E2F1 suppresses oxidative capacity by maintaining basal mitophagy.^134^ In addition, E2F1 cooperates with other transcriptional regulators such as NF-κB to fine-tune stress responses. For example, E2F1 and NF-κB coregulate *BNIP3*, a BH3-only protein that promotes autophagy and cell death. During hypoxia or RB1 loss, E2F1 induces *BNIP3* expression, whereas under basal conditions, NF-κB suppresses it to favor cell survival.^135^

In addition, E2F1 regulates mitochondrial dynamics by activating *MFN2*, a GTPase essential for mitochondrial fusion. E2F1 binds the *MFN2* promoter and acts cooperatively with Sp1, facilitating mitochondrial fusion while limiting mitophagy.^136^ In general, RB1 restrains E2F1 activity; however, RB1 loss leads to excessive E2F1 activation, resulting in uncontrolled *BNIP3* expression and autophagic cell death under hypoxic or nutrient-deprived conditions.^137^

In summary, E2F1 serves as a dual regulator of both autophagy and mitochondrial function. It coordinates the expression of genes involved in autophagosome formation (*LC3, ULK1*, and *DRAM1*), mitochondrial quality control (*BNIP3* and *MFN2*), and cell fate determination. By integrating cell cycle, metabolic, and stress signals, E2F1 positions itself at the intersection of autophagy regulation, mitochondrial dynamics, and context-dependent survival or death decisions.

### Stress-responsive transcription factors

Stress-responsive transcription factors, including p53, nuclear factor erythroid 2–related factor 2 (Nrf2), FOXOs, activating transcription factors **(**ATFs), and hypoxia-inducible factor-1α (HIF-1α), mediate cellular responses to diverse stressors, such as DNA damage, oxidative stress, hypoxia, ER stress, and metabolic imbalance. These factors regulate autophagy through transcription-dependent and transcription-independent mechanisms by modulating the expression of ATGs (such as *ULK1, BECN1, LC3*, and *ATG7*) and lysosomal components, as well as influencing upstream signaling pathways such as the mTOR, AMPK, and PI3K-AKT pathways. For example, nuclear p53 and FOXOs promote ATG expression, whereas cytoplasmic p53 and ATF5 act as autophagy repressors in certain contexts. Nrf2 regulates autophagic flux in coordination with redox homeostasis through the p62–Keap1–Nrf2 axis, whereas HIF-1α activates BNIP3–Beclin-1-dependent autophagy under hypoxic conditions (Fig. 7). Collectively, these transcription factors orchestrate flexible and context-specific autophagic responses that guide cellular adaptation, survival, or programmed death.

### p53

The tumor suppressor p53 (encoded by TP53) regulates a wide array of cellular processes, including cell cycle arrest, apoptosis, metabolism, and autophagy. The impact of p53 on autophagy is dual and compartment specific, functioning through both transcriptional and nontranscriptional mechanisms.

In the nucleus, p53 promotes autophagy by activating key ATGs such as *ULK1, ATG7, DRAM1*, and *ISG20L1*.^138^ Genome-wide binding studies have further identified *ULK2, ATG2B, ATG4, ATG10*, and *UVRAG* as direct transcriptional targets of p53.^8^ In addition, p53 represses mTOR signaling by upregulating the upstream inhibitors *AMPK, TSC2, PTEN*, and *SESN1/2*, thus increasing autophagic flux.^139^

Recent findings revealed that nuclear p53 also promotes autophagy through the FBXO22–TFEB axis. p53 induces the expression of FBXO22, which mediates the degradation of TFEB repressors (KDM4B, MYC, and NCOR1), thereby increasing TFEB activity and stimulating lysosomal and ATG expression.^140^ p53 also transcriptionally activates BH3-only proteins (*BAX, BAD, and BBC3/PUMA*), which disrupt BECN1–BCL-2/BCL-XL complexes, releasing Beclin 1 to promote autophagy.^141^ In addition, the p53-regulated factors *AEN* and *DAPK1* contribute to apoptosis and autophagy; DAPK1 enhances autophagy by phosphorylating BECN1.^142,143^

Conversely, cytoplasmic p53 acts as a negative regulator of autophagy through nontranscriptional mechanisms. Under normal conditions, it inhibits autophagy, an effect reversed by HDM2-mediated degradation of p53 during nutrient deprivation or stress.^139^ In support of this, p53-deficient cells display elevated basal autophagy through mTOR inhibition but exhibit defective autophagic flux under prolonged starvation, as evidenced by LC3 accumulation and impaired autophagosome clearance.^144,145^

p53 also influences mitophagy. In the cytoplasm, p53 interferes with Parkin translocation to depolarized mitochondria, thereby suppressing mitophagy and exacerbating oxidative stress.^146^ In the nucleus, it transcriptionally represses *PINK1*, a key mitophagy initiator, leading to reduced mitochondrial turnover.^147^

Certain p53 targets also act as autophagy suppressors. For example, *TIGAR* reduces ROS by redirecting glycolytic intermediates into the pentose phosphate pathway and enhancing NADPH production, thus blunting ROS-induced autophagy.^148^

In summary, p53 is a bifunctional regulator of autophagy, integrating cellular stress, metabolic status, and subcellular localization. Nuclear p53 induces autophagy through the transcriptional activation of ATGs, lysosomal genes, and upstream regulators such as TFEB, contributing to tumor suppression and stress adaptation. In contrast, cytoplasmic p53 inhibits general autophagy and mitophagy through nongenomic mechanisms. This duality highlights the complexity of the regulatory network of p53 at the crossroads of autophagy, metabolism, and cell fate determination.

#### Nrf2

Nrf2 is an important transcriptional regulator of oxidative stress responses that also interact tightly with autophagy pathways. Under homeostatic conditions, Nrf2 is retained in the cytoplasm through interaction with Keap1, which targets it for ubiquitin-mediated proteasomal degradation. Upon oxidative stress or impaired autophagic clearance, the autophagy adaptor p62 (SQSTM1) accumulates and binds to Keap1 through its KIR domain, displacing Nrf2 and facilitating its stabilization and nuclear translocation.^149,150^

Once in the nucleus, Nrf2 activates a suite of antioxidant response element-containing genes. *p62* itself is a transcriptional target of Nrf2, forming a positive feedback loop that enhances antioxidant defenses and autophagic capacity.^149,151^ This p62–Keap1–Nrf2 axis is increasingly recognized as a pivotal regulatory node coupling oxidative stress to autophagy. In autophagy-deficient cells lacking *Atg7* or *Atg5*, Nrf2 is constitutively activated as a result of p62 accumulation, leading to the induction of cytoprotective genes such as *NQO1, HO-1*, and enzymes involved in glutathione metabolism.^149,152^

Reciprocally, Nrf2 enhances autophagy by upregulating key genes, including *p62, ULK1, GABARAPL1*, and *NDP52*.^153,154^ This regulatory circuit supports cellular homeostasis in various disease models. In intervertebral disc degeneration, activation of Nrf2 through the p62–Keap1 loop enhances autophagy and reduces oxidative damage in nucleus pulposus cells.^153^ In ovarian granulosa cells, it protects against DEHP-induced autophagic stress and apoptosis.^154^ Nrf2 also enhances ATG expression to protect hepatocellular carcinoma cells from ferroptosis.^155^ Therefore, Nrf2 functions as a transcriptional effector and regulator of autophagy, with the p62–Keap1–Nrf2 axis serving as a critical redox‒autophagy interface in health and disease.

#### FOXOs

FOXO transcription factors (FOXO1, FOXO3, FOXO4, and FOXO6) are conserved regulators of stress resistance, metabolism, and lifespan and are currently recognized as key modulators of autophagy. They function through transcriptional activation of ATGs and cytoplasmic interactions with the autophagy machinery.

Upon nuclear translocation, FOXOs bind promoter regions of canonical ATGs, such as *LC3, ULK1, BECN1, and ATG12*, and lysosomal genes, including *GABARAPL1*, thereby increasing autophagosome formation and lysosomal degradation.^156–158^ FOXO3 was first identified as a direct activator of ATGs such as *ULK1* and *BNIP3* during skeletal muscle atrophy.^156^ In model organisms, orthologs such as dFOXO (*Drosophila*) and DAF-16 (*C. elegans*) similarly promote autophagy under starvation, linking FOXO function to metabolic adaptation and longevity.^97,159^

FOXO activity is tightly controlled by upstream nutrient and stress cues. Under nutrient sufficiency, FOXOs are phosphorylated by kinases such as AKT and sequestered in the cytoplasm, where they undergo degradation.^160^ In contrast, under nutrient deprivation, oxidative stress, or AMPK/SIRT1 activation, FOXOs are dephosphorylated and translocated into the nucleus to activate ATGs.^161^

FOXO1 exemplifies this dual mode of regulation. In the nucleus, it enhances the transcription of *BECN1, ATG12*, and *GABARAPL1*.^157^ In the cytoplasm, acetylated FOXO1 directly binds ATG7, stimulating autophagic flux independently of transcription.^162^ During ER stress, the UPR modulates FOXO1 through XBP1u: its loss stabilizes FOXO1 and promotes sustained autophagy.^163^ FOXO3 also contributes to the metabolic control of autophagy by upregulating glutamine synthetase, thereby increasing intracellular glutamine and disrupting mTORC1 lysosomal localization, which relieves the mTOR-mediated inhibition of autophagy.^164^

Despite their proautophagic roles, FOXOs may act as autophagy suppressors in specific contexts. In neurons, combined deletion of *FOXO1/3/4* unexpectedly increases autophagic flux, indicating a possible inhibitory role during development.^165^ In cancer, FOXOs exhibit dual behavior, either by suppressing tumor growth through autophagy activation or promoting survival under metabolic stress, depending on FOXO localization and the tumor context.

In conclusion, FOXO transcription factors function as versatile regulators of autophagy through the integration of nutrient, redox, and ER stress signals. They control autophagy at multiple levels, from gene transcription to direct modulation of autophagic proteins, enabling context-specific modulation of autophagy during development, metabolism, neurodegeneration, and cancer.

#### ATFs

ATFs are members of the bZIP transcription factor family that regulate stress responses, apoptosis, and autophagy. Among these proteins, ATF4, ATF5, and ATF6 play distinct roles in modulating autophagy, particularly under nutrient deprivation, hypoxia, oxidative stress, and ER stress.

ATF4 is a key mediator of the integrated stress response and promotes autophagy through direct transcriptional activation of ATGs. Under hypoxic or ER stress conditions, ATF4 upregulates BH3-only proteins such as *HRK, PUMA*, and *NOXA*, which influence mitochondrial integrity and autophagy.^166^ In addition, ATF4 binds to cAMP response elements (CREs) within the promoters of *LC3B* and *ULK1*, increasing autophagosome formation.^167,168^ This ULK1 induction is critical for initiating autophagy during ER stress and hypoxia, enabling tumor cell adaptation.^168^ Unlike PERK, which modulates autophagy at later stages, ATF4 acts early by activating ATG transcription, as shown in tunicamycin-stressed cells.^169^

Pharmacologically, ATF4 contributes to cytoprotective autophagy. In breast cancer cells, the proteasome inhibitor bortezomib induces ATF4, which increases *LC3B* expression and autophagic flux.^170^ Similarly, in lipopolysaccharide (LPS)-induced acute liver injury, IL-22 activates ATF4, which promotes ATG7 expression and LC3-II conversion, reducing inflammation through autophagy.^171^ In addition, ATF4 transcriptionally upregulates *ATG12* in response to ER stress, promoting autophagosome formation under proteotoxic conditions such as tunicamycin exposure.^169^ In hepatitis C virus infection, ATF4 plays a pivotal role in mediating autophagy activation by inducing *ATG12* and *MAP1LC3B* expression through the EIF2AK3- and ATF6-dependent unfolded protein response pathways, thereby promoting viral replication and cell survival.^172^

In contrast, ATF5 functions as a repressor of autophagy. In BCR-ABL-transformed cells, ATF5 is upregulated through the PI3K/AKT/FOXO4 axis and transcriptionally induces *mTOR*, a key inhibitor of autophagy, thus favoring leukemic cell survival.^173^ Therefore, ATF5 indirectly suppresses autophagy through mTOR activation, resulting in a pro-oncogenic role.

ATF6, which is activated during ER stress, contributes to autophagy in immune contexts. Under IFN-γ stimulation, ATF6 forms a complex with C/EBPβ to induce the expression of *DAPK1*, a kinase that promotes autophagy and mediates host defense against intracellular pathogens.^174^ ATF6 deficiency impairs this response, highlighting its role in immune-modulated autophagy.

Overall, ATFs regulate autophagy in a context-specific manner: ATF4 acts as a transcriptional activator of key ATGs during stress adaptation; ATF5 suppresses autophagy through mTOR signaling in oncogenic settings; and ATF6 links ER stress to immune-regulated autophagy. These divergent roles reveal the functional versatility of ATFs in maintaining cellular homeostasis, stress resilience, and immune defense.

#### HIF-1α

HIF-1α is a master regulator of cellular adaptation to hypoxia and promotes autophagy as a survival strategy under oxygen deprivation. Under normoxia, HIF-1α is degraded through proteasomes. Hypoxia stabilizes HIF-1α, enabling its nuclear translocation and activation of hypoxia-responsive genes, including those involved in autophagy.

One central pathway is the HIF-1α–BNIP3–Beclin-1 axis. HIF-1α upregulates *BNIP3*, which disrupts the BCL-2–Beclin-1 complex, releasing Beclin-1 to initiate autophagy.^175,176^ This mechanism supports protective autophagy in granulosa cells and cochlear marginal cells during hypoxic stress, which is consistent with the finding that the HIF-1α/Beclin1-mediated autophagy pathway confers neuroprotection under hypoxic preconditioning.^175,177,178^ In granulosa cells, HIF-1α-induced autophagy sustains glycolytic metabolism and mitochondrial clearance, thereby aligning energy production with survival needs.^179^ Lactate accumulation under hypoxia further amplifies this cascade, reinforcing metabolic adaptation.^175^

HIF-1α regulates autophagy by suppressing mTOR signaling through distinct hypoxia-responsive effectors. The HIF-1α target BNIP3 interacts with Rheb, a key activator of mTOR, thereby mediating hypoxia-induced inhibition of mTOR activity.^180^ In a parallel pathway, DDIT4 (REDD1) associates with 14-3-3 proteins and releases TSC2, which subsequently acts as a GTPase-activating protein to inhibit Rheb, resulting in mTORC1 inactivation and autophagy induction under hypoxic conditions.^181^

HIF-1α also cooperates with other transcription factors to regulate autophagy. In pancreatic β cells, the HIF-1α–FOXO1 axis promotes autophagy under hypoxic conditions, supporting insulin secretion and cell viability.^182^ Thus, HIF-1α regulates autophagy through multiple effectors, including BNIP3, Beclin-1, REDD1, and FOXO1, thereby linking oxygen sensing to autophagic homeostasis in diverse physiological and pathological contexts.

### Metabolic regulators

**Fig. 8:**
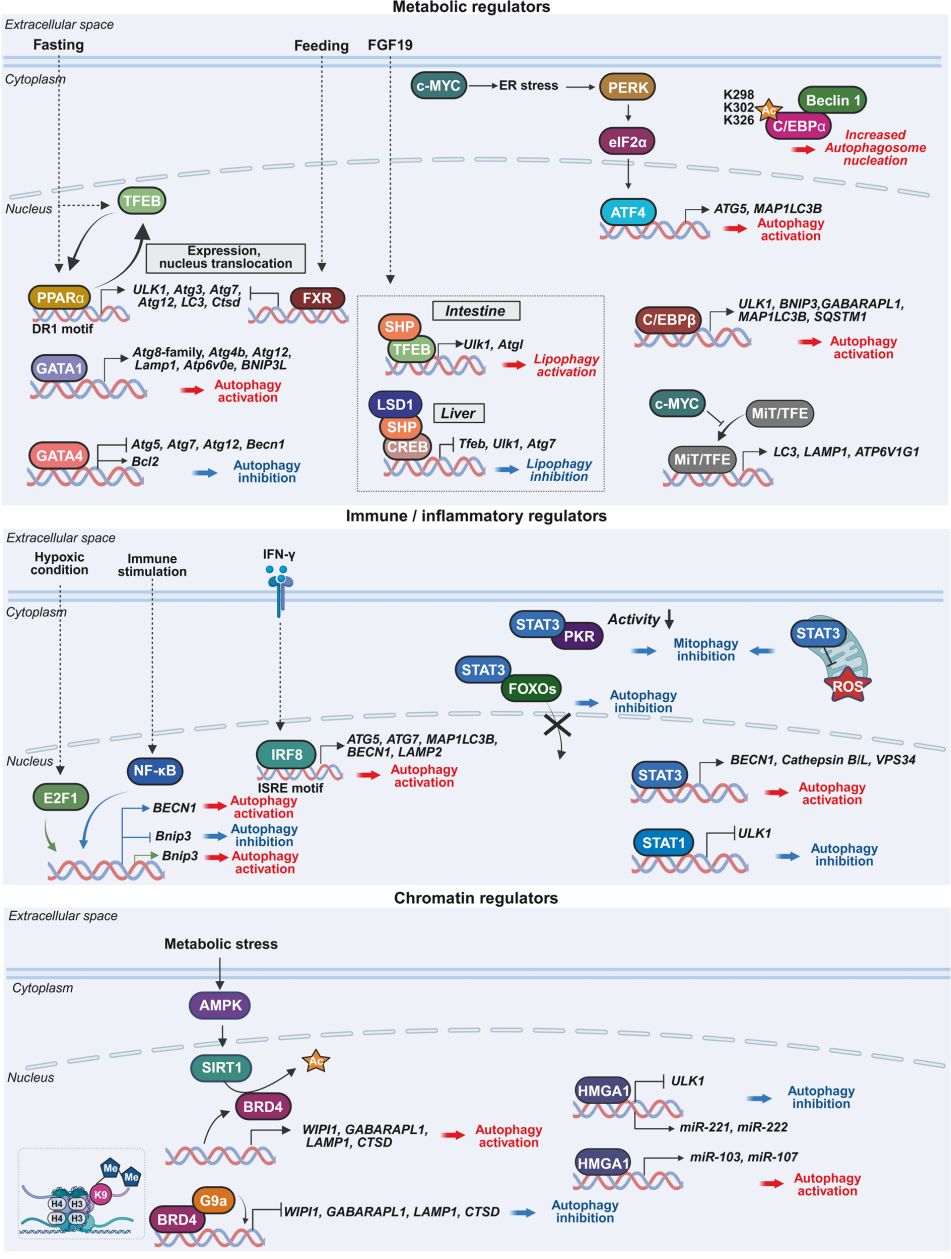
Transcriptional regulation of autophagy by metabolic, immune, and chromatin regulators. This figure summarizes how transcription factors involved in metabolic, immune, and chromatin regulation control autophagy in response to nutrient signals, inflammation, and epigenetic context. (Top) Metabolic regulators: PPARα activates DR1-containing ATG promoters (such as *ULK1, Atg3/7/12, LC3*, and *Ctsd*) during fasting and cooperates with TFEB to drive lipophagy; FXR counters fasting signals in the fed state by disrupting CREB/CRTC2 on ATG promoters and repressing TFEB, whereas PPARα–FXR competition temporally gates autophagy. SHP acts as a nutrient-sensitive switch: in the liver, a CREB–SHP–LSD1 complex demethylates H3K4me2 at *Tfeb, Ulk1*, and *Atg7* to repress autophagy, whereas in the intestine, SHP cooperates with TFEB to promote lipophagy (*Ulk1* and *Atgl*). GATA1 induces the expression of autophagy/mitophagy genes during erythroid maturation, whereas GATA4 suppresses autophagy in cardiomyocytes through BCL2–BECN1 inhibition and the downregulation of core ATGs. C/EBPβ activates *ULK1, BNIP3, GABARAPL1, MAP1LC3B*, and *SQSTM1*, and C/EBPα (K298/K302/K326) facilitates autophagosome nucleation through acetylation-dependent interactions with Beclin 1. MYC increases intracellular ER stress, which subsequently activates the PERK–eIF2α–ATF4 signaling pathway, leading to the upregulation of ATGs such as *ATG5* and *MAP1LC3B*. MYC interferes with the MiT/TFE transcription factors to repress autophagy. (Middle) Immune/inflammatory regulators: NF-κB represses *Bnip3* and mitophagy under normoxia by competing with E2F1 for binding to the *Bnip3* promoter, thereby preventing *Bnip3*-mediated mitochondrial clearance. However, during hypoxic stress or upon NF-κB inhibition, E2F1 access is restored, *Bnip3* expression is induced, and mitophagy is activated to promote mitochondrial quality control and cell death. Under immune activation, NF-κB promotes autophagy by transcriptionally inducing *BECN1*, which is reinforced by the IAP–NF-κB–BECN1 signaling axis. IRF8 enhances antimicrobial autophagy by upregulating core ATGs (*ATG5/7, MAP1LC3B*, and *BECN1*) and fusion genes (LAMP2), linking IFN-γ and TLR signaling to xenophagy. STATs regulate autophagy in a compartment-dependent manner: nuclear STAT3 activates autophagy and lysosomal genes, whereas cytoplasmic or mitochondrial STAT3 inhibits autophagy through the PKR–eIF2α and FOXO pathways; STAT1 suppresses *ULK1* transcription to limit autophagy initiation. (Bottom) Chromatin regulators: BRD4 recruits G9a to deposit H3K9me2 at ATG loci (such as *WIPI1, GABARAPL1, LAMP1*, and *CTSD*), repressing autophagy; metabolic stress or BET inhibition activates AMPK–SIRT1 to evict BRD4 and restore flux. HMGA1 either inhibits autophagy by repressing *ULK1* transcription or promotes it through miRNA-mediated regulatory pathways, depending on the cellular context. The figure was created with BioRender.com

Metabolic transcription factors, including peroxisome proliferator-activated receptor alpha (PPARα), farnesoid X receptor (FXR, NR1H4), small heterodimer partner (SHP, NR0B2), GATA-binding protein (GATA), CCAAT/enhancer-binding protein (C/EBP) family members, and the MYC proto-oncogene (MYC), integrate nutrient status with autophagic responses. These regulators modulate ATG expression during fasting, feeding, lipid accumulation, and stress. PPARα controls autophagy and lipophagy in a context-dependent manner. FXR and SHP function as nutrient-sensitive switches that activate or repress autophagy on the basis of the tissue environment and metabolic signals, particularly in the liver and intestine. The GATA and C/EBP families contribute to autophagy during differentiation and inflammation, influencing mitophagy and immune signaling. MYC, a central regulator of metabolism and proliferation, plays dual roles: it may repress autophagy through MiT/TFE antagonism or promote it to support tumor metabolism (Fig. 8). Collectively, these factors coordinate autophagic gene programs with metabolic demands, maintaining homeostasis and enabling stress adaptation.

#### PPARα

PPARα is a ligand-activated nuclear receptor predominantly expressed in the liver, heart, and skeletal muscle. Traditionally known for regulating fatty acid oxidation, PPARα also acts as a transcriptional activator of autophagy, especially during fasting.

Upon nutrient deprivation, PPARα binds to direct repeat-1 (DR1) elements within the promoters of ATGs such as *ULK1, Atg3, Atg7, Atg12, LC3*, and *Ctsd*, inducing their transcription.^183,184^ This regulation involves competition with FXR, which occupies DR1 sites and recruits corepressors under nutrient-rich conditions to suppress autophagy. During fasting, PPARα displaces FXR and recruits coactivators such as PGC1α, increasing ATG expression and autophagosome formation.^183^

PPARα also promotes lipophagy, the autophagic degradation of lipid droplets, through a positive feedback loop with TFEB, a master lysosomal regulator. TFEB enhances *PPARα* expression during nutrient stress, whereas PPARα activation (such as fenofibrate) induces TFEB expression and nuclear translocation, amplifying lysosomal gene expression and calcium-mediated clearance of hepatic lipid droplets.^185,186^ This PPARα–TFEB axis holds therapeutic promise for MAFLD and related disorders.^186^

In disease contexts, PPARα-mediated autophagy has protective effects. In acute liver failure models, PPARα activation increases ATGs (*LC3, BECN1*) and suppresses proinflammatory cytokines (such as TNF-α and IL-6), alleviating liver injury.^187^ In AD models, PPARα activation (such as through gemfibrozil) enhances autophagic flux, facilitates Aβ clearance, and improves cognitive function through AMPK-mTOR signaling and ATG induction.^188^

In summary, PPARα links fasting signals to autophagy by activating DR1-containing ATG promoters and antagonizing FXR repression. Its cooperation with TFEB enhances lipophagy and lysosomal biogenesis, thereby supporting cellular clearance and metabolic adaptation. These mechanisms highlight the dual role of PPARα as a transcriptional and metabolic regulator in health and disease.

#### NR1H4/FXR

FXR, encoded by NR1H4, is a bile acid–activated nuclear receptor highly expressed in the liver and intestine. Although it primarily regulates bile acid, lipid, and glucose metabolism, FXR also serves as a nutrient-sensitive transcriptional repressor of autophagy through direct and indirect modulation of ATG expression.

In the fed state, elevated bile acid levels activate FXR, which translocates to the nucleus and binds to the promoters of ATGs such as *ATG7, ULK1*, and *TFEB*. FXR represses these genes by disrupting the CREB/CRTC2 complex, a key driver of fasting-induced autophagy. Specifically, FXR interacts with CRTC2, preventing its association with CREB at ATG promoters, thereby inhibiting the transcription of genes required for autophagosome initiation and lysosomal biogenesis.^184^

During fasting, FXR is antagonized by PPARα, which competes for shared DNA response elements on ATG promoters. PPARα, which is activated under nutrient-deprived conditions, induces ATG expression and promotes autophagic flux.^183^ This FXR–PPARα antagonism provides a mechanistic basis for the temporal control of autophagy on the basis of nutrient status. In addition, FXR indirectly suppresses lysosomal biogenesis by repressing *TFEB* transcription. Reduced TFEB expression impairs its nuclear translocation and limits the activation of lysosomal gene programs, thereby further inhibiting its autophagic capacity.^184^

In summary, FXR integrates bile acid and nutritional signals to downregulate autophagy through the inhibition of CREB/CRTC2 signaling and the suppression of TFEB-mediated transcription. By acting as a transcriptional brake during feeding, FXR dynamically regulates autophagy and lysosomal function in coordination with energy status.

#### NR0B2/SHP

SHP, encoded by NR0B2, is an atypical orphan nuclear receptor that functions as a transcriptional corepressor through protein–protein interactions instead of direct DNA binding. SHP modulates autophagy in a highly context-dependent manner, exerting opposing effects on different tissues.

In the liver, feeding-induced FXR activation elevates SHP expression, which forms a complex with CREB to recruit lysine-specific demethylase 1 (LSD1) to the promoters of ATGs such as *Tfeb, Ulk1*, and *Atg7*. This CREB–SHP–LSD1 complex mediates epigenetic repression of autophagy by demethylating H3K4me2, which is a histone marker associated with transcriptional activation.^189^ This FGF19–SHP–LSD1 axis silences ATG expression, serving as a nutrient-responsive repressive circuit that prevents excessive autophagic activity under nutrient-rich conditions.

Conversely, in the intestine, SHP enhances autophagy—specifically lipophagy—after nutrient intake. In this context, SHP functions downstream of FGF15/19 signaling and cooperates with TFEB to upregulate *Ulk1* and *Atgl*, initiating lipid droplet degradation and lowering postprandial triglyceride and ApoB48 levels.^190^ This tissue-specific shift from transcriptional repression to coactivation reflects the flexible integration of local metabolic signals through SHP.

SHP also plays an antifibrotic role in hepatic stellate cells (HSCs). SHP overexpression downregulates ATGs, reduces autophagic flux, and inhibits the expression of fibrosis markers such as α-SMA, TIMP-1, and COL1A1. In contrast, SHP knockdown increases autophagy and promotes fibrogenesis, highlighting its role in limiting autophagy-driven HSC activation.^191^

Overall, SHP serves as a versatile transcriptional regulator of autophagy. It represses autophagy in the liver through LSD1 recruitment, promotes lipophagy in the intestine through TFEB interaction, and suppresses fibrogenic autophagy in HSCs. These diverse functions highlight the role of SHP in autophagy‒metabolism crosstalk and its therapeutic relevance in liver disease and metabolic regulation.

#### GATAs

GATA transcription factors, which are defined by conserved zinc finger DNA-binding domains, are key regulators of differentiation, proliferation, and metabolism. Recent studies have revealed that GATA family members modulate autophagy in a highly context-dependent and tissue-specific manner, functioning as either transcriptional activators or repressors.

GATA1 has been characterized as a transcriptional activator of autophagy during hematopoietic and erythroid maturation. It directly binds the promoters of multiple ATGs, including *Atg8* family members, *Atg4b, Atg12*, and lysosomal genes, such as *Lamp1* and *Atp6v0e*, thereby driving their expression and promoting the autophagy‒lysosome network.^192^ In addition, GATA1 induces *BNIP3L* (NIX), a mitophagy effector essential for mitochondrial clearance during late-stage erythropoiesis. This GATA1-driven transcriptional program ensures the generation of mature red blood cells devoid of mitochondria.^192^

In contrast, GATA4 acts predominantly as an autophagy suppressor in postmitotic tissues such as the heart. In cardiomyocytes, GATA4 represses autophagy by upregulating *Bcl2*, which binds and inhibits BECN1, thereby blocking autophagosome initiation.^193^ Furthermore, GATA4 downregulates essential ATGs, including *Atg5, Atg7, Atg12*, and *Becn1*, collectively diminishing autophagic flux.^193,194^ Doxorubicin-induced stress promotes proteasomal degradation of GATA4, thereby potentially increasing this repression to enable adaptive autophagy activation.

In addition to its role in cardiomyocytes, GATA4 plays a role in cellular senescence. In senescent cells, decreased expression of the autophagy receptor p62/SQSTM1 prevents GATA4 degradation, enabling its accumulation. Stabilized GATA4 activates the transcription of senescence-associated secretory phenotype (*SASP*) genes, including proinflammatory cytokines and chemokines that reinforce senescence and tissue remodeling.^195^

In summary, GATA1 promotes autophagy and mitophagy during erythroid differentiation, whereas GATA4 inhibits autophagy in cardiomyocytes and enhances inflammatory signaling during senescence. These dual roles highlight the nuanced, tissue-specific regulation of autophagy by GATA factors in development and stress adaptation.

#### C/EBP family (C/EBPβ and C/EBPα)

C/EBPs are basic leucine zipper transcription factors that regulate cell differentiation, metabolism, and inflammatory responses. Among these, C/EBPβ and C/EBPα have emerged as key regulators of autophagy, each of which mediates distinct mechanisms across different tissues and stress contexts.

C/EBPβ directly activates the transcription of ATGs such as *ULK1, BNIP3*, *GABARAPL1, MAP1LC3B*, and *SQSTM1*, supporting phagophore formation and substrate targeting.^196^ This regulation underlies circadian autophagy, as C/EBPβ loss disrupts time-dependent autophagic rhythms and impairs metabolic stability.^196^ During adipogenesis, C/EBPβ binds the *Atg4b* promoter, increasing *Atg4b* expression, enhancing LC3 processing, and facilitating the degradation of KLF2/3 through p62-mediated autophagy to promote adipocyte differentiation.^197^ Under immune activation, C/EBPβ cooperates with ATF6 to induce *DAPK1* expression, which enhances autophagy through ERK1/2 signaling.^174^ In *Toxoplasma gondii*-infected cells, C/EBPβ suppresses mTOR signaling, increases LC3-II formation, and enhances lysosomal acidification, contributing to pathogen clearance.^198^ Notably, the LIP isoform of C/EBPβ, which lacks a transactivation domain, promotes autophagic cell death in breast cancer by increasing autophagosome formation and LC3 lipidation.^199^

C/EBPα primarily modulates mitophagy in HSCs. It interacts with Beclin 1, a core PI3KC3 complex component, to facilitate autophagosome nucleation. This interaction is acetylation dependent and requires lysine residues K298, K302, and K326.^200^

Overall, C/EBPβ and C/EBPα regulate autophagy by activating core ATGs and interacting with essential autophagy machinery. They also integrate upstream signals such as mTOR, IFN-γ, and ERK1/2 to fine-tune autophagic responses to metabolic and infectious stress. Their complementary yet distinct actions highlight the functional versatility of C/EBPs in coordinating general and selective autophagy.

#### MYC

The MYC proto-oncogene, a bHLHZip transcription factor, is a central regulator of cellular proliferation and metabolism. Recent findings revealed that MYC also modulates autophagy in a highly context-dependent manner, functioning as either a suppressor or activator depending on oncogenic signaling, metabolic state, and tissue type.

In many cancers, MYC represses autophagy by interfering with MiT/TFE transcription factors (TFEB, TFE3, and FOXH1), which activate lysosomal and ATGs. MYC competes for promoter binding sites at loci such as *LC3, LAMP1*, and *ATP6V1G1*, thereby inhibiting their transcription.^201^ This repression is reversible; HDAC inhibition disrupts the chromatin occupancy of MYC, restoring MiT/TFE-mediated gene activation.^201^ In HPV-positive head and neck cancer, MYC stabilization impairs MiT/TFE activity and autophagic flux, contributing to lysosomal dysfunction and enhancing cisplatin sensitivity.^202^ In *Penaeus vannamei*, MYC orthologs downregulate *BECN1* and *LC3-II*, whereas MYC silencing promotes autophagy and cell survival under stress, revealing evolutionary conservation of its inhibitory role.^203^ In *C. elegans*, MYC acts downstream of the ABL–ERK pathway to suppress *WIPI1*, limiting autophagosome formation and intercellular autophagic transfer, with implications for lifespan regulation.^204^

Conversely, MYC elevates intracellular ER stress, which in turn activates the PERK–eIF2α–ATF4 signaling pathway, leading to the upregulation of ATGs such as *ATG5* and *MAP1LC3B*. This ER stress–mediated autophagy is essential for sustaining MYC-driven cellular transformation and tumor growth by alleviating proteotoxic stress and maintaining metabolic homeostasis.^205,206^

Overall, MYC regulates autophagy through multiple transcriptional and epigenetic mechanisms. It can suppress autophagy by antagonizing MiT/TFE transcription factors and repressing lysosomal and ATG gene expression but can enhance autophagy under ER stress by activating the PERK–eIF2α–ATF4 signaling pathway to promote autophagy gene induction. This dual functionality positions MYC as a central integrator of oncogenic, metabolic, and stress-adaptive pathways, with its impact on autophagy determined by the cellular context. Its context-dependent capacity to both inhibit and activate autophagy reveals the importance of MYC as a dynamic regulator and potential therapeutic target in cancer biology.

### Immune/inflammatory regulators

Immune-related transcription factors, including NF-κB, interferon regulatory factor 8 (IRF8), and signal transducer and activator of transcription (STATs), play pivotal roles in regulating autophagy during infection and inflammation. NF-κB represses mitophagy under normoxia by inhibiting BNIP3 but promotes autophagy during immune activation by inducing *BECN1* expression. IRF8 enhances pathogen clearance by upregulating ATGs and lysosomal components in response to IFN-γ. STATs exhibit compartment-specific effects: nuclear STAT3 promotes autophagy by activating autophagy and lysosomal genes, whereas cytoplasmic and mitochondrial STAT3 inhibit autophagy by repressing the PKR and FOXO pathways. In contrast, STAT1 suppresses autophagy through the transcriptional silencing of *ULK1* (Fig. 8). These transcription factors integrate inflammatory and metabolic cues with autophagic responses, thereby facilitating immune defense, pathogen clearance, and cellular adaptation to stress.

#### NF-κB

NF-κB is a key transcription factor that integrates immune, inflammatory, and stress signals to regulate diverse biological processes, including autophagy. NF-κB is activated through cytokines (such as TNF-α and IL-1β), oxidative stress, pathogen-associated molecular patterns (PAMPs), and DNA damage, which stimulate the IκB kinase (IKK) complex to phosphorylate IκB inhibitors. This leads to IκB degradation and the nuclear translocation of NF-κB subunits (such as p65/RelA and p50), enabling the transcription of target genes.

NF-κB modulates autophagy in a context-dependent manner. Under normoxic or homeostatic conditions, NF-κB inhibits mitophagy by repressing *Bnip3* transcription. Specifically, NF-κB competes with E2F1 for binding to the *Bnip3* promoter, thereby preventing Bnip3-mediated mitochondrial clearance. However, during hypoxic stress or upon NF-κB inhibition, E2F1 access is restored, Bnip3 expression is induced, and mitophagy is activated to promote cell death and mitochondrial quality control.^135^ In contrast, NF-κB promotes autophagy during immune stimulation. For example, T-cell receptor engagement induces NF-κB-mediated transcriptional upregulation of *BECN1* (Beclin 1), facilitating autophagy initiation.^207^ NF-κB-binding sites have been identified in the *Beclin 1* promoter, indicating direct transcriptional control by NF-κB in multiple cell types.^207^

NF-κB-driven autophagy is further enhanced through its interaction with inhibitor of apoptosis proteins (IAPs). XIAP and cIAP1 activate NF-κB signaling and increase *Beclin 1* levels, thereby promoting autophagosome formation. Inhibition of these IAPs suppresses NF-κB activity and impairs autophagic flux, thus highlighting the importance of the IAP–NF-κB–BECN1 axis in sustaining autophagy.^208^

Conversely, functional autophagy is also necessary for proper NF-κB signaling. In *Atg5*- or *Atg7*-deficient mouse embryonic fibroblasts, canonical NF-κB activation in response to nutrient deprivation, mTOR inhibition, or p53 loss is markedly reduced. These findings indicate that autophagy is required for optimal NF-κB nuclear translocation and transcriptional activity.^209^ Overall, NF-κB plays dual roles in autophagy regulation: it represses *Bnip3*-dependent mitophagy under normoxia but promotes autophagic activation under inflammatory or immune stimuli by inducing *BECN1*. In parallel, autophagy sustains NF-κB functionality, indicating a bidirectional regulatory loop that links cellular stress responses with immune and metabolic homeostasis.

#### IRF8

IRF8 is a hematopoietic lineage-restricted transcription factor crucial for myeloid differentiation and antimicrobial immunity. Recent studies have revealed that IRF8 directly governs ATG expression during infection, IFN-γ signaling, and metabolic stress.^210,211^

Mechanistically, IRF8 binds interferon-stimulated response elements in the promoters of key ATGs, including *ATG5, ATG7, MAP1LC3B*, and *BECN1*, thereby increasing autophagosome formation.^211^ It also upregulates genes critical for autophagosome–lysosome fusion, such as *VAMP8* and *LAMP2*, ensuring the completion of autophagic flux.^211^ In macrophages, IRF8 activation is potentiated by IFN-γ, which increases IRF8 nuclear localization and facilitates cooperation with PU.1 and STAT1 to promote the expression of autophagy and lysosomal genes. In parallel, microbial PAMPs and TLR signaling pathways activate IRF8 through MyD88 and TRIF adaptors, linking pathogen recognition to autophagic defense.

IRF8 also regulates the generation of mitochondrial ROS, which are critical signals for xenophagy. IRF8-deficient cells exhibit impaired LC3 lipidation, SQSTM1/p62 accumulation, and lysosomal dysfunction, resulting in defective clearance of intracellular pathogens such as *Listeria monocytogenes*.^211^ These defects impair phagosome maturation, reduce antimicrobial activity, and compromise myeloid cell development. In summary, IRF8 functions as a transcriptional activator of autophagy under immune and infectious stress. By coordinating the expression of core ATGs and lysosomal components, IRF8 promotes antimicrobial autophagy and links IFN-γ and TLR signaling with cellular clearance pathways and host defense.

#### STAT

STAT proteins are key mediators of cytokine and growth factor signaling and regulate immune responses, proliferation, apoptosis, and autophagy. Following cytokine stimulation, STATs are phosphorylated by Janus kinases (JAKs), dimerize, and translocate to the nucleus to activate gene expression programs.

Among the STAT family members, STAT3 plays dual roles in autophagy regulation depending on its subcellular localization. In the nucleus, STAT3 promotes autophagy by transcriptionally activating key autophagy and lysosomal genes, including *BECN1, cathepsin B/L*, and *VPS34*, thereby enhancing autophagosome formation and degradation capacity.^212^ This nuclear function supports autophagic flux in diverse physiological and pathological contexts such as cancer, ischemia–reperfusion injury, and stem cell homeostasis.^212,213^

Conversely, cytoplasmic and mitochondrial STAT3 inhibit autophagy. Cytoplasmic STAT3 suppresses autophagy by binding and inhibiting PKR kinase, which otherwise activates eIF2α phosphorylation and ATG expression under stress.^214^ STAT3 also sequesters FOXO transcription factors in the cytoplasm, preventing the nuclear transactivation of ATGs such as *LC3* and *ATG12*.^215^ In addition, mitochondrial STAT3 limits excessive ROS production, thereby curbing ROS-induced mitophagy.^212^ Pharmacological inhibition or siRNA-mediated knockdown of STAT3 enhances autophagic flux, whereas STAT3 overexpression reduces palmitate- or drug-induced autophagy, revealing its context- and dose-dependent suppressive effects.^216^

In contrast, STAT1 primarily acts as a transcriptional repressor of autophagy. STAT1 binds directly to the *ULK1* promoter and suppresses its expression, thereby limiting autophagy initiation. Chromatin immunoprecipitation assays confirmed this repressive interaction.^217^ As a result, STAT1-deficient cells presented increased ULK1 expression and elevated autophagy levels. In PD models, *Stat1* ablation alleviated α-synuclein fibril-induced suppression of microglial *ULK1* transcription and autophagy, which is consistent with the mechanism whereby α-synuclein disrupts microglial autophagy through STAT1-dependent repression of *ULK1* expression.^218^

In summary, STAT proteins govern autophagy in a compartment- and stimulus-specific manner. Nuclear STAT3 promotes autophagy through the transcriptional activation of autophagy-lysosomal genes, whereas cytoplasmic and mitochondrial STAT3 suppress autophagy by inhibiting the PKR and FOXO signaling pathways. STAT1 represses autophagy through direct silencing of *ULK1* transcription. These findings highlight the importance of STATs as versatile regulators of autophagy and potential therapeutic targets in cancer, inflammatory disease, and tissue injury.

### Chromatin regulators

Chromatin regulators such as BRD4 and HMGA1 serve as epigenetic modulators of autophagy by influencing transcriptional accessibility and chromatin architecture at ATG loci. BRD4 represses autophagy and lysosomal gene expression by recruiting G9a to deposit repressive H3K9me2 marks, a block that is lifted through AMPK–SIRT1 signaling under metabolic stress. Its inhibition induces autophagic flux in cancer, inflammation, and infection models but may also promote EMT, depending on the context. HMGA1 similarly modulates autophagy in a tissue-specific manner, suppressing *ULK1* transcription and activating mTOR in cancer and cardiomyopathy while promoting miRNA-mediated autophagy activation in neurodegenerative models (Fig. 8). Through direct gene repression and indirect regulation through microRNAs, BRD4 and HMGA1 influence how chromatin dynamics intricately govern autophagic homeostasis across disease contexts.

#### BRD4

BRD4, a member of the BET family, is known for its roles in transcriptional elongation, cell proliferation, and oncogenesis. Recently, BRD4 was identified as a transcriptional repressor of autophagy and lysosomal biogenesis, independent of TFEB/TFE3/MITF-mediated mechanisms. BRD4 recruits G9a (EHMT2) to ATG loci such as *WIPI1*, *GABARAPL1, LAMP1*, and *CTSD*, promoting H3K9 dimethylation and transcriptional repression.^219^ This suppression bypasses CLEAR motifs and MiT/TFE binding, instead imposing an epigenetic block on gene accessibility. Under metabolic stress or pharmacological inhibition, the AMPK–SIRT1 axis is activated; SIRT1 deacetylates and displaces BRD4 from chromatin, restoring ATG transcription and flux.^219^ In NUT midline carcinoma, aberrant BRD4 chromatin retention disrupts autophagy and lysosomal function, revealing therapeutic vulnerability.^219^

BRD4 inhibition induces autophagy-related cell death in multiple cancers. In breast cancer, it disrupts BRD4–AMPK interactions, releasing AMPK–mTOR–ULK1 signaling to promote autophagosome formation and cancer cell death.^220^ In CRC, combined BRD4 and HDAC inhibition further enhances autophagic cytotoxicity and suppresses IL-6–JAK–STAT3 signaling.^221^ However, in esophageal squamous cell carcinoma, BRD4 inhibition induces EMT and migration through autophagy activation, indicating potential adverse effects.^222^

In addition to its role in oncology, BRD4 regulates autophagy. In acute pancreatitis, BRD4 inhibition restores autophagosome–lysosome fusion through SIRT1, reducing inflammation and tissue injury.^223^ During *Mycobacterium tuberculosis* infection, BRD4 acts downstream of EGFR to repress macrophage lipophagy. Its inhibition reduces lipid accumulation and bacterial burden, improving host defense.^224^ Collectively, BRD4 functions as a critical epigenetic suppressor of autophagy, with diverse and often opposing effects across cancer, infectious, and inflammatory diseases. The ability of BRD4 to either limit protective autophagy or sustain oncogenic survival renders BRD4 a complex yet promising therapeutic target.

#### HMGA1

HMGA1 is a nonhistone chromatin architectural protein that influences gene expression by altering DNA conformation and facilitating transcriptional complex assembly. It regulates autophagy by modulating ATG expression, intersecting with ULK1–mTOR signaling, and engaging miRNA networks in a context-dependent fashion.

In cancer, HMGA1 functions primarily as an autophagy suppressor. It represses *ULK1* transcription and activates mTOR, inhibiting autophagosome formation. HMGA1 knockdown restores *ULK1* expression and enhances LC3B lipidation, although autophagosome maturation may remain impaired.^225^ In bladder cancer, HMGA1 promotes the expression of *miR-221*, which targets TP53INP1, a p53-regulated autophagy enhancer. TP53INP1 suppression activates ERK signaling, thereby promoting tumor migration and growth.^226^ In contrast, HMGA1 promotes autophagy in neurodegenerative contexts. In PD models, HMGA1 elevates the levels of *miR-103* and *miR-107*, which activate the CDK5R1/CDK5 pathway, thereby increasing lysosomal function and aggregate clearance. This creates a feedback loop that preserves neuronal viability in MPTP-induced models.^227^ In contrast, HMGA1 contributes to cardiac dysfunction by suppressing autophagy in diabetic cardiomyopathy. It induces *miR-222*, which targets p27Kip1, resulting in CDK2/mTOR activation and the repression of ATGs. HMGA1 silencing restores autophagy and improves cardiac function.^228^

Overall, HMGA1 orchestrates autophagy through transcriptional repression of *ULK1*, mTOR pathway activation, and miRNA-mediated regulation of TP53INP1 and p27. Its opposing roles, i.e., its ability to suppress autophagy in cancer and cardiac disease while enhancing it in neurodegeneration, reveal its complex, tissue-specific regulatory capacity. These properties highlight HMGA1 as a potential therapeutic target in autophagy-related pathologies.

## Autophagy regulation by epigenetic regulators

### Regulation of autophagy by histone acetyltransferases

**Fig. 9:**
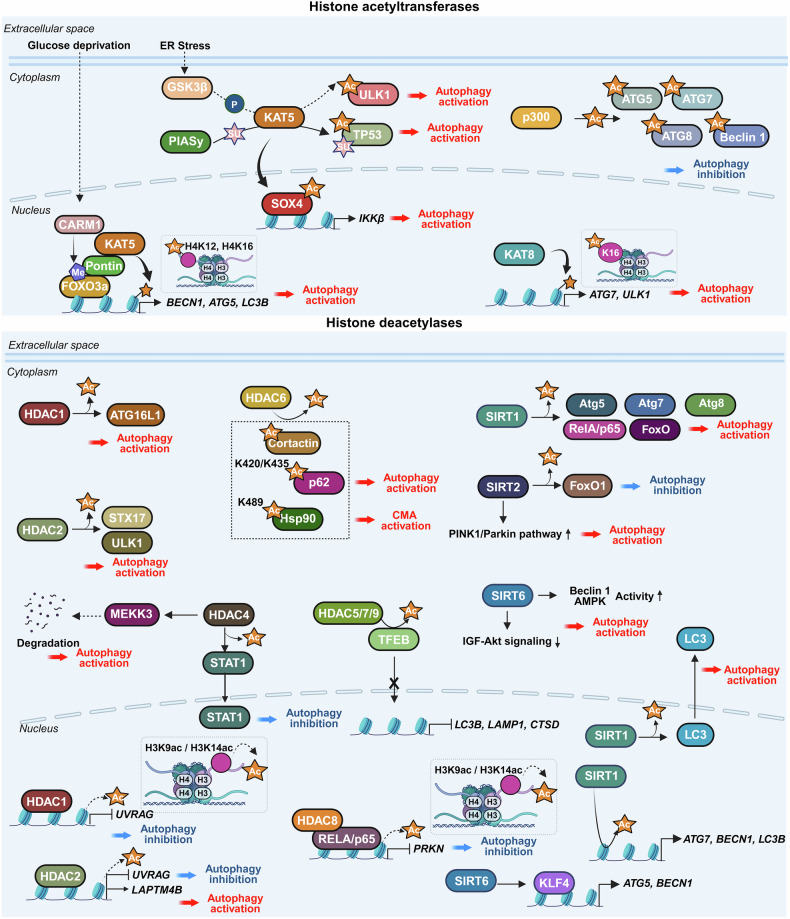
Epigenetic regulation of autophagy by histone acetyltransferases (HATs) and histone deacetylases (HDACs). This figure illustrates how epigenetic modifiers, including histone acetyltransferases and deacetylases, regulate autophagy through chromatin remodeling and posttranslational modifications of autophagy-related proteins. (Top) HATs: Histone acetyltransferases (KATs) relax chromatin at ATG promoters to support transcriptional activation. KAT5/Tip60 is recruited to ATG loci by CARM1-methylated Pontin and cooperates with FOXO3a during glucose starvation to acetylate H4 and activate *BECN1, ATG5*, and *LC3B*; KAT5 also acetylates ULK1 upon ER stress/growth-factor withdrawal (downstream of GSK3β) and acetylates p53 to promote stress-induced autophagy. Furthermore, KAT5 acetylates SOX4 and activates *IKKβ* transcription, thereby promoting autophagy‒lysosome fusion and increasing neuronal survival. KAT8 regulates H4K16ac at ATG promoters (such as *ATG7* and *ULK1*), thereby promoting ATG expression and autophagosome formation. p300 acetylates nonhistone autophagy proteins (ATG5, ATG7, ATG8, and Beclin 1) and generally represses autophagic flux under nutrient-rich conditions. (Bottom) Histone deacetylases (HDACs): Class I/II/IV zinc-dependent HDACs and Class III NAD⁺-dependent sirtuins regulate autophagy through histone deacetylation and direct modification of the autophagy machinery. Class I: HDAC1/HDAC2 compacts chromatin at ATG promoters to repress transcription and deacetylates ATG16L1, STX17, and ULK1, facilitating autophagosome elongation and lysosomal fusion. HDAC2 also activates *LAPTM4B* transcription, thereby promoting hepatocellular carcinoma progression and linking autophagy regulation with oncogenic signaling. HDAC8 forms a complex with RELA/p65 to silence *PRKN*, suppressing mitophagy under metabolic stress. Class II: HDAC4 plays dual, context-dependent roles in autophagy. In cancer, HDAC4 promotes tumor growth by enhancing the autophagic degradation of MEKK3, thereby reducing its stability and favoring cell survival. In contrast, under diabetic or hyperglycemic conditions, HDAC4 represses autophagy in podocytes by deacetylating STAT1 and promoting chromatin compaction at autophagy loci, which impairs autophagic clearance and contributes to renal dysfunction. HDAC5, HDAC7, and HDAC9 regulate TFEB acetylation and thereby control its nuclear translocation, ultimately modulating lysosomal gene expression and autophagic capacity. HDAC6 promotes autophagy through cytoplasmic substrate deacetylation—cortactin (which enhances actin polymerization), p62 (K420/K435) (which improves ubiquitinated cargo recognition), and Hsp90 (K489) (which facilitates CMA via the LAMP-2A interaction). Sirtuins: SIRT1 deacetylates H4K16ac, Atg5, Atg7, LC3, and FoxO, promoting autophagy under nutrient stress. SIRT2 exerts bidirectional control: FOXO1 deacetylation inhibits autophagy through the FOXO1–ATG7 interaction, whereas under stress, SIRT2 activates mitophagy through the PINK1/Parkin pathway. SIRT6 enhances autophagy by upregulating Beclin 1 and AMPK, suppressing IGF–Akt signaling, and increasing KLF4-mediated transcription of *ATG5* and *BECN1*. The figure was created with BioRender.com

Histone acetylation at lysine residues of histones H3 and H4, particularly H3K9, H3K14, and H4K16, marks transcriptionally active chromatin. This modification is catalyzed by histone acetyltransferases (HATs) (also known as lysine acetyltransferases (KATs)) and reversed by histone deacetylases (HDACs). At ATG promoters, histone acetylation relaxes chromatin structure, facilitates transcription factor and RNA polymerase II recruitment, and promotes ATG expression and autophagy induction.^229,230^ In addition to histones, HATs acetylate autophagy-related proteins, modulating their stability, localization, and interactions, thereby coordinating the transcriptional and posttranslational control of autophagy (Fig. 9).

#### KAT5/Tip60

KAT5, also known as Tip60, regulates autophagy through histone and nonhistone acetylation. During glucose starvation, Tip60 is recruited to ATG promoters by CARM1-methylated Pontin, where it interacts with FOXO3a to acetylate H4 and activate *BECN1, ATG5*, and *LC3B* transcription.^229^ This is associated with elevated H4K12 and H4K16 acetylation, which enhances promoter accessibility. Tip60 also acetylates ULK1 in response to ER stress and growth factor withdrawal following activation by GSK3β.^231,232^ In yeast, the Tip60 homolog Esa1 acetylates Atg3 at K19/K48, facilitating its interaction with Atg8 and promoting autophagosome elongation.^233^ Reduced Tip60 activity leads to hypoacetylation of ATG loci and impaired transcription. Furthermore, Tip60 acetylates p53 at K120, whereas PIASy sumoylates it at K386, producing a “binary death signal” that induces autophagy.^234^ In AD models, Tip60 acetylates SOX4 and activates *IKKβ* transcription, enhancing autophagy‒lysosome fusion and neuronal survival.^235^

#### hMOF/KAT8

hMOF, also known as KAT8, is an MYST family member that specifically acetylates H4K16, a marker of transcriptionally active chromatin. During autophagy induction, global and promoter-specific H4K16ac levels, including those at *ATG7* and *ULK1*, decrease due to hMOF downregulation, indicating that reduced hMOF activity limits excessive autophagy and facilitates proper autophagic flux.^230^ Maintaining high H4K16ac levels disrupts autophagy and increases apoptosis, highlighting the need for dynamic acetylation control. An siRNA screen identified KAT8 as a positive autophagy regulator; its knockdown reduces autophagosome formation and ATG expression, confirming its role in sustaining chromatin accessibility for autophagy-related transcription.^236^

#### p300

Posttranslationally, p300 acetylates ATG5, ATG7, ATG8, and Beclin 1, generally inhibiting their activity and autophagic flux.^237,238^ Specifically, Beclin 1 acetylation at K430/K437, following CK1-mediated phosphorylation at S409, promotes Rubicon binding and blocks autophagosome maturation, whereas deacetylation restores autophagic progression and tumor-suppressive function.^238^ p300 is activated by mTORC1-dependent phosphorylation, thereby linking nutrient sensing to autophagy inhibition.^239^ BAT3 counteracts p300 by promoting p53 acetylation, while preventing ATG7 acetylation, which in turn favors autophagy.^240^ Pharmacological inhibition of p300 enhances autophagy; for example, 5-fluorouracil suppresses p300/CBP, decreases histone acetylation, and induces autophagy in colorectal cancer.^241^ Similarly, natural compounds such as spermidine and resveratrol inhibit p300 activity, leading to global hypoacetylation and activation of autophagy.^118,242^

### Regulation of autophagy by HDACs

HDACs remove acetyl groups from lysines on histone tails, leading to chromatin condensation and transcriptional repression. At ATG loci, this erases activating marks such as H3K9ac, H3K14ac, and H4K16ac, thereby limiting ATG expression and autophagy initiation. HDACs also deacetylate nonhistone targets, including autophagy core proteins, transcription factors, and scaffolding proteins, regulating autophagic flux, vesicle maturation, and cargo clearance. The mammalian genome encodes 18 HDACs, which are grouped into four classes: Class I HDACs (HDAC1, 2, 3, and 8) are primarily nuclear; Class II HDACs (HDAC4, 5, 6, 7, 9, and 10) shuttle between the nucleus and cytoplasm; Class III HDACs (SIRT1–7) are NAD⁺-dependent sirtuins; and Class IV HDACs (HDAC11) share features of Classes I and II HDACs. Although Classes I, II, and IV require zinc for catalytic activity, sirtuins uniquely depend on NAD⁺. HDACs influence autophagy epigenetically by shaping chromatin accessibility at *ATG* loci and posttranslationally through the deacetylation of key autophagy regulators (Fig. 9).

#### HDAC1 and HDAC2

HDAC1 and HDAC2 are key epigenetic regulators of autophagy that function through transcriptional repression and posttranslational modification. At the chromatin level, they remove active histone marks (H3K9ac and H3K14ac) at promoters of ATGs such as *UVRAG*, resulting in transcriptional silencing. Under nutrient-rich conditions, FOXK1 and FOXK2 recruit the Sin3A–HDAC1/2 complex to autophagy gene promoters, inducing H4 deacetylation and repressing the initiation of starvation-induced atrophy and autophagy programs, thereby maintaining metabolic quiescence.^243–245^ In CRC, HDAC1 inhibition induces *UVRAG* and promotes autophagy during 5-fluorouracil treatment.^243^ In skeletal muscle, HDAC1/2 maintain autophagic flux under stress conditions.^244^ In addition, HDAC1 enhances basal autophagy by deacetylating ATG16L1, aiding in autophagosome elongation and supporting nutrient-deprived cell proliferation.^246^ Notably, HDAC inhibitors (HDACis) exert context-dependent effects and can enhance protective autophagy in tumors while suppressing maladaptive autophagy in cardiac hypertrophy.^247,248^

Similarly, HDAC2 regulates autophagy at the chromatin and protein levels. It interacts with the MYC–MiT/TFE axis to influence autophagy and lysosomal gene expression.^201^ HDAC2 also activates *LAPTM4B* transcription, promoting hepatocellular carcinoma progression.^249^ In chronic kidney disease, HDAC2 enhances autophagic flux and reduces vascular calcification.^250^

Posttranslationally, HDAC2 deacetylates STX17, facilitating autophagosome–lysosome fusion.^251^ It also deacetylates TPD52 isoform 1, regulating CMA in prostate cancer,^252^ and modulates ULK1 activity at K68 to coordinate pyroptosis and autophagy in acute liver failure.^253^ Collectively, HDAC1 and HDAC2 shape autophagy through chromatin remodeling and the deacetylation of autophagy proteins, affecting tumor progression, vascular health, skeletal muscle adaptation, and inflammatory disease.

#### HDAC4

HDAC4 plays dual roles in autophagy, modulating chromatin states and protein acetylation in a context-dependent manner. In gastric cancer, HDAC4 promotes tumor growth by enhancing the autophagic degradation of MEKK3, thereby reducing its stability and favoring cell survival.^254^ In vascular inflammation, HDAC4 activates autophagy through NF-κB-mediated chromatin remodeling and ATG transcription.^255^ In contrast, in diabetic nephropathy, HDAC4 represses autophagy in podocytes by deacetylating STAT1 and promoting chromatin compaction at autophagy loci under hyperglycemic conditions, leading to impaired autophagic clearance.^256^ These findings indicate that HDAC4 can either promote or inhibit autophagy depending on the cellular context, metabolic status, and disease setting.

#### HDAC5

HDAC5 regulates autophagy through chromatin maintenance and transcription factor modulation. It reinforces heterochromatin structure by promoting histone deacetylation at pericentromeric regions, limiting ATG transcription. HDAC5 depletion leads to heterochromatin disorganization, genomic instability, and DNA damage responses that trigger autophagy and apoptosis.^257^ In addition, HDAC5 controls autophagy posttranslationally by deacetylating TFEB, a key transcription factor for lysosomes and ATGs. Inhibition of HDAC5 (such as through trichostatin A) increases TFEB acetylation, increasing its nuclear translocation and increasing the expression of target genes such as *LC3B, LAMP1*, and *CTSD*.^258^ This dual mechanism, which combines chromatin repression and TFEB modulation, positions HDAC5 as a crucial node for integrating nuclear structure and autophagy signaling.

#### HDAC6

HDAC6 regulates autophagy primarily through cytoplasmic deacetylation of nonhistone proteins instead of through chromatin remodeling. It modulates autophagosome trafficking and fusion by targeting key substrates involved in cytoskeletal dynamics.

Functionally, HDAC6 promotes autophagosome maturation by deacetylating cortactin, increasing actin polymerization and microtubule-mediated transport.^259^ In PD, HDAC6 supports mitophagy by facilitating mitochondrial clearance, which is impaired in parkin-deficient models.^260^ In liver cancer, HDAC6 induces Beclin1-dependent autophagic cell death, supporting tumor suppression.^261^ However, its effects are context specific: in metastatic prostate cancer, elevated HDAC6 expression impairs autophagy and promotes EMT and tumor aggressiveness.^262^ In glioblastoma, HDAC6 sustains autophagy and cancer stemness, contributing to radioresistance.^263^

HDAC6 also links autophagy with the ubiquitin‒proteasome system by deacetylating protein aggregates and facilitating their autophagic degradation.^264^ It also regulates p62/SQSTM1 acetylation (K420/K435), which is critical for recognizing ubiquitinated protein aggregates,^265^ and Hsp90 acetylation (K489), which enhances its interaction with LAMP-2A to promote CMA and reduce α-synuclein aggregation in PD.^266^ Collectively, HDAC6 coordinates macroautophagy, mitophagy, and CMA through nonhistone targets, with key roles in cancer, neurodegeneration, and metabolic disease.

#### HDAC7

HDAC7 represses autophagy through transcriptional and posttranslational mechanisms in a context-dependent manner. Although its effects on histone acetylation at ATG promoters are not well characterized, HDAC7 is known to silence gene expression through histone deacetylation. In salivary mucoepidermoid carcinoma, HDAC7 silencing triggers apoptosis and autophagy, indicating that its inhibition activates a stress-induced autophagic program.^267^ Mechanistically, HDAC7 regulates autophagy through the lysine crotonylation–dependent 14-3-3ε–PPM1B pathway, which responds to leucine deprivation and links nutrient signaling to autophagic control.^268^ In AD-like tauopathies, HDAC7 suppresses lysosomal biogenesis by deacetylating TFEB, thereby inhibiting its nuclear translocation and reducing the expression of genes such as *LAMP2* and *CTSD*.^269^ This impairs lysosomal function and exacerbates tau accumulation. Therefore, HDAC7 acts as a suppressor of autophagy through the modulation of transcription factors and nutrient-responsive signaling, which is relevant in neurodegenerative and oncogenic conditions.

#### HDAC8

HDAC8 negatively regulates autophagy primarily through transcriptional repression and chromatin compaction. Although its specific histone targets at ATG loci remain unclear, HDAC8 is implicated in maintaining a repressive chromatin state. In high-glucose–exposed neurons, HDAC8 forms a complex with RELA to repress *PRKN* (Parkin) transcription, suppressing mitophagy. This is likely mediated by HDAC8-catalyzed removal of active histone acetylation marks, such as H3K9ac and H3K14ac. Sodium butyrate treatment disrupts this complex, restoring *PRKN* expression and mitophagy.^270^ Overall, HDAC8 enforces the transcriptional repression of ATGs, especially under metabolic stress, thereby positioning it as a potential therapeutic target. In support of this, pharmacological inhibition of HDAC8 in breast cancer cells triggers both apoptosis and autophagy, indicating a switch from pro-survival to pro-death signaling upon HDAC8 blockade.^271^ A novel HDAC8 inhibitor was shown to promote autophagic vacuole formation and LC3-II accumulation, highlighting the therapeutic potential of targeting HDAC8 to induce autophagic cell death in cancer.

#### HDAC9

HDAC9 is a context-dependent modulator of autophagy that functions primarily through chromatin-based gene silencing. It represses autophagy by deacetylating histones near ATG promoters, thereby inhibiting the transcriptional activation of autophagy programs. In hypoxic myoblasts, HDAC9 upregulation impairs differentiation by suppressing autophagy, likely through H3K9ac or H4K16ac deacetylation, although the precise sites involved remain undefined.^272^ Similarly, in mesenchymal stem cells, HDAC9-mediated autophagy suppression contributes to osteoporosis-like phenotypes, indicating chromatin tightening at ATG loci.^273^

In addition to histones, HDAC9 modulates autophagy through nonhistone substrates. It regulates TFEB acetylation, a key event in lysosomal biogenesis. HDAC9 inhibition enhances TFEB acetylation and nuclear localization, increasing the transcription of lysosomal genes such as *LAMP1* and *CTSD* and improving AD-related phenotypes in vivo.^258^ Thus, HDAC9 controls autophagy at multiple levels through histone deacetylation and TFEB modulation, thereby linking chromatin dynamics to disease and development.

#### HDAC10

HDAC10 promotes autophagy-dependent cell survival, acting primarily through nonhistone protein deacetylation. Although its direct histone targets remain unclear, HDAC10 may influence chromatin and ATG expression under nutrient stress. In cancer and stress models, HDAC10 facilitates the clearance of toxic protein aggregates, thereby preserving proteostasis.^274^ In the liver, HDAC10 activation is correlated with autophagy induction and growth suppression during nutrient restriction, suggesting roles in chromatin remodeling and growth checkpoint regulation.^275^ Although its epigenetic substrates are still under investigation, the cytoplasmic functions of HDAC10, particularly in autophagosome–lysosome fusion, highlight its role as a noncanonical autophagy regulator integrating nuclear and cytosolic responses.

#### Sirtuins

Sirtuins (SIRT1–SIRT7) are NAD⁺-dependent class III HDACs that regulate autophagy through histone deacetylation and nonhistone protein modification.

SIRT1 is the most studied sirtuin in autophagy. Epigenetically, it deacetylates H4K16ac, counteracting hMOF activity to promote chromatin condensation and ATG activation (such as *ATG7, BECN1*, and *LC3B*).^230,276^ It also antagonizes BRD4 at lysosomal and ATG promoters, maintaining a hypoacetylated state favorable for transcription.^219^ At the posttranslational level, SIRT1 deacetylates Atg5, Atg7, Atg8, NF-κB, and FoxO, enhancing autophagy initiation and gene expression.^161,277,278^ SIRT1 also promotes nuclear LC3 deacetylation and cytoplasmic translocation during starvation.^279^ SIRT1 deficiency impairs autophagy induction under nutrient-deprived conditions, leading to metabolic imbalance and cellular stress.

SIRT2 exerts context-specific control over autophagy. Under stress, SIRT2 dissociation permits FOXO1 acetylation, enabling FOXO1–ATG7 interaction and autophagy; conversely, SIRT2 deacetylates FOXO1 to restrain this pathway. Cytosolic FOXO1 has been shown to be essential for autophagy induction by directly binding ATG7, independent of its transcriptional function, thus supporting the above mechanism. This finding highlights that SIRT2-mediated deacetylation of FOXO1 serves as a critical switch controlling the balance between autophagy activation and suppression under stress conditions.^162^ It enhances mitophagy by deacetylating mitochondrial proteins and activating the PINK1/Parkin pathway, thereby promoting mitochondrial quality control and supporting autophagy during glucocorticoid-induced muscle atrophy.^280,281^ However, SIRT2 has dual functions: its knockdown can increase basal autophagy while preventing apoptosis during mitotic arrest,^282^ whereas in renal podocytes, it suppresses autophagy and impairs proliferation.^283^

SIRT6 regulates autophagy through transcriptional and signaling mechanisms. It enhances KLF4 expression, which in turn promotes the transcription of ATGs such as *ATG5* and *BECN1*.^284^ In esophageal cancer, SIRT6 activates Beclin 1 and AMPK, suppressing mTOR and increasing autophagic flux.^285^ In addition, it inhibits IGF-Akt signaling and activates ULK1 in bronchial epithelial cells, promoting autophagy and delaying senescence.^286^

In summary, SIRT1, SIRT2, and SIRT6 integrate metabolic and epigenetic signals to regulate autophagy. They modulate chromatin through histone deacetylation (such as H4K16ac through SIRT1), activate stress-responsive transcription factors, influence AMPK/mTOR signaling, and support autophagy under nutrient-deprived and pathological conditions.

### Regulation of autophagy by acyl-CoA synthetase short-chain family member 2

Acyl-CoA synthetase short-chain family member 2 (ACSS2) functions as a metabolic‒epigenetic integrator, converting acetate into acetyl-CoA, a key donor for histone acetylation. Under glucose deprivation, AMPK phosphorylates ACSS2 at S659, inducing its nuclear translocation. In the nucleus, ACSS2 interacts with TFEB and facilitates site-specific H3K9 acetylation at the promoters of autophagy and lysosomal genes, including *LAMP1, ATG5*, and *GABARAPL1*, thus increasing transcription and promoting cell survival under stress.^287^

**Fig. 10:**
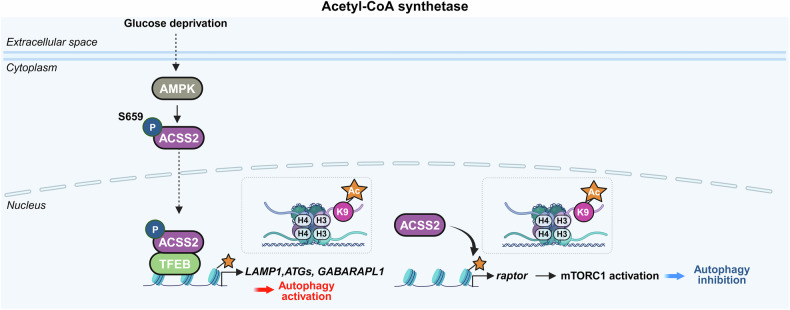
Regulation of autophagy by ACSS2 under nutrient stress conditions. This figure illustrates the dual regulatory role of acyl-CoA synthetase short-chain family member 2 (ACSS2) in autophagy through metabolic‒epigenetic coupling. Under glucose deprivation, AMPK phosphorylates ACSS2 at serine 659, triggering its translocation from the cytosol into the nucleus. Once inside the nucleus, phosphorylated ACSS2 interacts with TFEB and promotes the transcription of ATGs by catalyzing localized acetyl-CoA production and enhancing histone H3K9 acetylation at their promoter regions. This epigenetic modification upregulates genes such as *LAMP1, ATGs*, and *GABARAPL1*, thereby supporting autophagy induction and stress adaptation. In addition, ACSS2 contributes to the transcriptional activation of autophagy through raptor-dependent chromatin remodeling. However, in specific pathological contexts, such as diabetic nephropathy, nuclear ACSS2 enhances H3K9 acetylation at the raptor promoter, activating mTORC1 signaling and repressing autophagy, ultimately promoting podocyte injury. Thus, ACSS2 serves as a context-dependent regulator of autophagy, functioning either as a promoter of autophagy under energy stress by facilitating histone acetylation of ATG loci or as a repressor through mTORC1 activation in pathological states. The figure was created with BioRender.com

ACSS2 plays divergent roles across disease contexts. In breast cancer, cadmium exposure inhibits ACSS2, suppressing ATG5-mediated autophagy and promoting tumorigenesis.^288^ In contrast, in subarachnoid hemorrhage models, ACSS2 activation upregulates *ATG5* expression and enhances autophagy, reducing neuronal apoptosis and brain edema.^289^ In ovarian cancer, ACSS2 inhibition suppresses glycolysis and induces its nuclear translocation, activating a SIRT1/ATG5/ATG2B axis that promotes autophagy and reduces malignancy.^290^ However, in diabetic nephropathy, ACSS2 enhances H3K9 acetylation at the *raptor* promoter, activating mTORC1 and repressing autophagy, leading to podocyte injury. ACSS2 inhibition reverses this effect, restoring autophagic flux and improving kidney function (Fig. 10).^291^ In summary, ACSS2 modulates autophagy bidirectionally, thereby promoting autophagy by increasing histone acetylation in ATGs during energy stress and repressing it under other conditions by activating mTORC1 through chromatin remodeling.

### Autophagy regulation by histone, DNA, and RNA methyltransferases

**Fig. 11:**
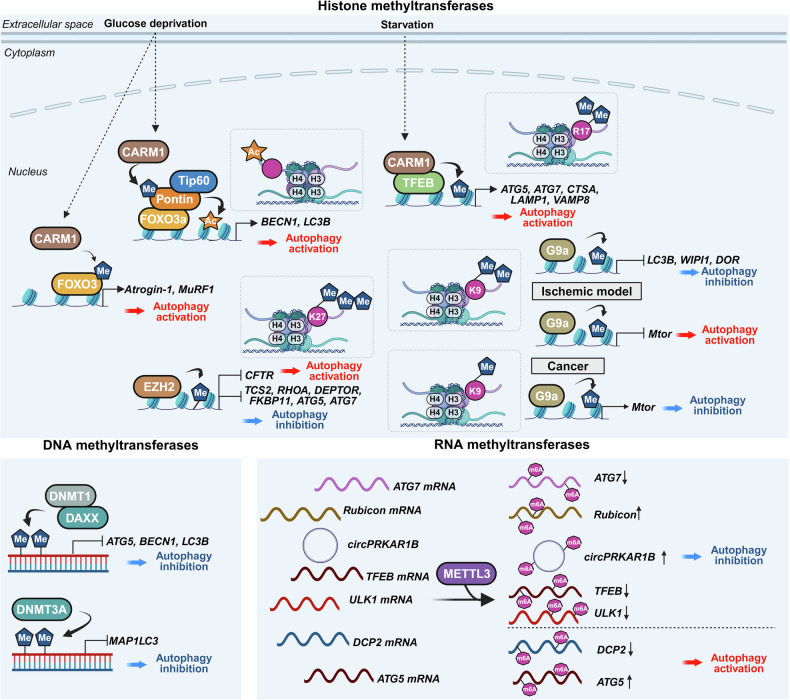
Epigenetic and posttranscriptional regulation of autophagy by histone, DNA, and RNA methyltransferases. This figure depicts how various classes of methyltransferases regulate autophagy through histone modification, DNA methylation, and RNA methylation mechanisms. (Top) Histone methyltransferases: Histone methyltransferases such as CARM1, G9a, and EZH2 modulate chromatin accessibility at autophagy gene loci to control transcriptional activity. CARM1 catalyzes the dimethylation of histone H3 at arginine 17 (H3R17me2), enhancing TFEB recruitment and the expression of ATGs under starvation conditions. CARM1 also interacts with Pontin and FOXO3 to form a complex with Tip60, promoting H4 acetylation and the transcriptional activation of genes such as *BECN1* and *LC3B*. It further methylates FOXO3 to increase the expression of atrophy-associated genes. G9a serves as a context-dependent modulator of autophagy. Under basal or lipotoxic conditions, it suppresses autophagy by depositing H3K9me2 at the promoters of *LC3B, WIPI1*, and *DOR*, repressing their transcription. In cancer, G9a enhances H3K9me1 at the *mTOR* promoter, activating mTOR signaling and inhibiting autophagy to promote tumor growth. In contrast, during ischemic preconditioning, G9a-mediated H3K9me2 deposition at the Mtor promoter downregulates mTOR and induces protective autophagy. EZH2, the catalytic subunit of the PRC2 complex, adds the repressive mark H3K27me3 at promoters of autophagy-related and mTOR-regulatory genes such as *TSC2, RHOA*, and *ATG5*, thereby suppressing autophagy. In hepatic tissue, EZH2 suppresses *CFTR* transcription, leading to autophagy activation through an epigenetic mechanism involving both DNA methylation and EZH2-dependent H3K27me3. (Bottom left) DNA methyltransferases: DNMT1 and DNMT3A silence ATGs by catalyzing DNA methylation at CpG islands within ATG promoters. DNMT1 functions with the corepressor DAXX to suppress genes such as *ATG5* and *LC3B*, whereas DNMT3A specifically represses *MAP1LC3* under chronic stress. (Bottom right) RNA methyltransferases: The RNA methyltransferase METTL3 modifies the mRNAs of ATGs through N6-methyladenosine (m6A) marks, influencing their stability and translational efficiency. METTL3 can repress autophagy by methylating transcripts such as *ATG7, Rubicon, circPRKAR1B, TFEB*, or *ULK1*, thereby impairing autophagic flux and lysosomal biogenesis in diseases such as osteoarthritis, Crohn’s disease, and ischemic heart injury. Conversely, METTL3 promotes autophagy in cancer by methylating mRNAs such as *DCP2* and *ATG5*, enhancing mitophagy and contributing to chemoresistance. These diverse outcomes highlight the transcript-specific and context-dependent roles of METTL3 in controlling autophagy. The figure was created with BioRender.com

Histone, DNA, and RNA methyltransferases regulate autophagy through chromatin and RNA modifications. HMTs such as CARM1, G9a, and EZH2 control ATG transcription through H3R17, H3K9, or H3K27 methylation, linking chromatin to nutrient and stress cues. DNMT1/3 A repress ATGs through CpG methylation, impacting immunity, development, and aging. METTL3 modifies autophagy via m^6^A methylation of ATG mRNAs in a context-dependent manner. Collectively, these enzymes coordinate autophagy across physiological and disease states (Fig. 11).

#### CARM1

CARM1, also known as PRMT4, regulates autophagy through histone and nonhistone methylation. It dimethylates histone H3 at arginine 17 (H3R17me2), an activating mark that opens chromatin and enables TFEB recruitment to autophagy and lysosomal gene promoters under starvation.^292^ This action enhances transcription and autophagic flux in nutrient-deprived cells. CARM1 stability is maintained through the AMPK–SKP2–CARM1 axis, making it a nutrient-sensitive epigenetic modulator.^292^ In skeletal muscle, CARM1 sustains autophagy and mitophagy during fasting through AMPK–ULK1 and FOXO3 signaling, thereby preserving muscle integrity.^293^

CARM1 also indirectly regulates chromatin through nonhistone protein methylation. Under glucose deprivation, it methylates Pontin, promoting the formation of a Pontin–FOXO3a–Tip60 complex. This complex recruits Tip60 to ATG promoters, inducing H4 acetylation and the upregulation of genes such as *BECN1* and *LC3B*.^229^ In addition, CARM1 methylates FOXO3, increasing its transcriptional activity and increasing the expression of atrophy-related genes such as *Atrogin-1* and *MuRF1*, thus connecting autophagy regulation to muscle degradation.^294^ Overall, CARM1 acts as a central integrator of autophagy regulation through H3R17me2-mediated transcription, the recruitment of chromatin-modifying complexes, and nonhistone methylation, operating across diverse physiological and pathological contexts.

#### G9a

G9a, also known as EHMT2, is a lysine methyltransferase that catalyzes mono- and dimethylation of H3K9 (H3K9me1/2), which is typically associated with transcriptional repression. It acts as a dual-function epigenetic regulator of autophagy, with effects varying depending on the cellular context. Under basal or lipotoxic conditions, G9a represses autophagy by enriching H3K9me2 at the promoters of *LC3B, WIPI1*, and *DOR*, thereby silencing their transcription and reducing their autophagic activity.^295^ In hepatocytes exposed to fatty acids, the mTORC1–G9a–H3K9me2 axis suppresses *BECN1* and *ATG7*, reinforcing autophagy inhibition. G9a inhibition reverses these effects and restores ATG expression and autophagic flux.^296^ In cancer, G9a enhances H3K9me1 at the *mTOR* promoter in gastric cancer cells, activating *mTOR* transcription and suppressing autophagy, thereby accelerating tumor growth.^297^ In ischemic preconditioning models, G9a deposits H3K9me2 at the *Mtor* promoter, reducing its expression and enhancing autophagy, which contributes to cardioprotection.^298^

Overall, G9a has context-dependent effects: it suppresses autophagy through H3K9me1/2-mediated gene silencing in metabolic and tumor stress settings, whereas under nutrient deprivation or ischemia, it supports autophagy by repressing mTOR. This duality reveals its dynamic role in chromatin remodeling and its therapeutic relevance in cancer and stress resistance.

#### EZH2

EZH2, the catalytic subunit of PRC2, mediates the trimethylation of H3K27 (H3K27me3), a repressive histone mark that silences gene expression. As a negative regulator of autophagy, EZH2 represses key autophagy-related and mTOR-regulatory genes in oncogenic and vascular contexts.

In CRC, EZH2 suppresses the upstream mTOR inhibitors *TSC2, RHOA, DEPTOR*, and *FKBP11* by recruiting MTA2 and increasing H3K27me3 at their promoters, thereby activating mTOR signaling and inhibiting autophagy.^299^ In addition, EZH2 represses *AMBRA1* and *LC3B*, further reducing autophagic flux. The knockdown or pharmacologic inhibition of EZH2 derepresses these genes, leading to autophagy induction, G1 arrest, and apoptosis in CRC cells.^300^

In VSMCs, EZH2 restrains excessive autophagic death by suppressing *ATG5* and *ATG7* expression. Its inhibition results in autophagic vacuole accumulation and VSMC loss through MEK–ERK1/2 activation, a mechanism implicated in aortic dissection.^301^ In hepatic tissue, homocysteine has been shown to activate autophagy by downregulating cystic fibrosis transmembrane conductance regulator (CFTR) expression through the coordinated action of DNA methylation and EZH2-mediated H3K27me3, revealing epigenetic crosstalk between methylation pathways in autophagy regulation.^302^ These findings highlight the ability of EZH2 to modulate autophagy through chromatin repression as well as by linking autophagy to cellular stress responses.

In summary, EZH2 primarily suppresses autophagy through H3K27me3-mediated silencing of ATG and mTOR pathway regulators. Its inhibition reverses transcriptional repression, reactivating autophagy and providing therapeutic potential in autophagy-deficient tumors and vascular disorders.

#### DNMT1 and DNMT3A

The DNA methyltransferases DNMT1 and DNMT3A mediate methylation at CpG sites, typically leading to stable transcriptional silencing. These enzymes epigenetically regulate autophagy by modulating chromatin accessibility at ATG loci.

DNMT1 represses autophagy through interaction with the transcriptional corepressor DAXX. This complex is recruited to the promoter regions of *ATG5, BECN1*, and *LC3B*, where DNMT1 facilitates CpG methylation and silencing.^303^ Disruption of the DNMT1–DAXX interaction derepresses these ATGs and reactivates autophagic flux. In cytokine-induced memory-like NK cells, DNMT1 modulates autophagy through the AMPK/mTOR axis; its knockdown enhances autophagy and improves NK cell function and immunological memory.^304^

DNMT3A contributes to long-term repression of ATGs through de novo methylation, particularly under chronic stress and aging. It specifically targets *MAP1LC3* isoforms, leading to transcriptional silencing and impaired autophagy.^305^ During embryonic development, DNMT1 and DNMT3A are essential for maintaining appropriate autophagy levels. Their knockdown in mouse zygotes results in the accumulation of *p62/SQSTM1*, indicating defective autophagic degradation and disrupted cellular stress responses.^306^

In summary, DNMT1 and DNMT3A repress autophagy by methylating CpG islands near the transcription start sites of key ATGs. These mechanisms are implicated in immune regulation, development, and age-related pathologies, thus highlighting the broad physiological importance of DNA methylation in the control of autophagy.

#### METTL3

METTL3, the catalytic core of the m^6^A RNA methyltransferase complex, posttranscriptionally regulates autophagy by modifying the mRNAs of ATGs, thereby affecting their stability, translation, or degradation in a context-dependent manner.

In degenerative and inflammatory diseases, METTL3 primarily suppresses autophagy. In osteoarthritis, METTL3 methylates *ATG7* mRNA, reducing its stability and expression, which promotes fibroblast-like synoviocyte senescence and cartilage breakdown through the autophagy–GATA4 pathway.^307^ In MAFLD, METTL3 enhances m^6^A modification of *Rubicon* mRNA, stabilizing this autophagy inhibitor and impairing autophagosome–lysosome fusion, leading to lipid accumulation and hepatic steatosis.^308^ In Crohn’s disease, METTL3 increases m^6^A methylation on *circPRKAR1B*, a circular RNA that inhibits autophagy and activates the NLRP3 inflammasome, promoting intestinal inflammation.^309^ METTL3 also represses autophagy in ischemic heart disease by methylating *TFEB* mRNA, decreasing its stability and nuclear translocation, thereby impairing lysosomal biogenesis and exacerbating myocardial injury during hypoxia–reoxygenation.^310^ In the ovary, METTL3-mediated methylation of *ULK1* mRNA in granulosa cells reduces ULK1 expression and autophagy, contributing to follicular atresia and reproductive aging.^311^

Conversely, METTL3 can enhance autophagy in cancer. In small cell lung cancer, METTL3 promotes chemoresistance through activation of mitophagy. It enhances m^6^A methylation of *DCP2* mRNA, which stabilizes mitochondrial mRNA decay processes and activates the PINK1–Parkin mitophagy pathway under chemotherapeutic stress.^312^ In VSMCs, METTL3 suppresses mTOR signaling by reducing phosphorylated mTOR, thereby inducing autophagosome formation and inhibiting pathological proliferation.^313^ Similarly, in cisplatin-resistant seminomas, METTL3 stabilizes *ATG5* mRNA through m^6^A modification, increasing autophagic flux and reducing drug sensitivity.^314^

In summary, METTL3 exerts dual, transcript-specific effects on autophagy. It represses autophagy in degenerative and metabolic disorders by destabilizing or activating inhibitory transcripts while promoting autophagy in cancers by stabilizing key ATG mRNAs or enhancing mitophagy pathways. These diverse roles position METTL3 as a context-sensitive regulator of autophagy and a potential therapeutic target across multiple disease states.

### Autophagy regulation by histone demethylases

**Fig. 12:**
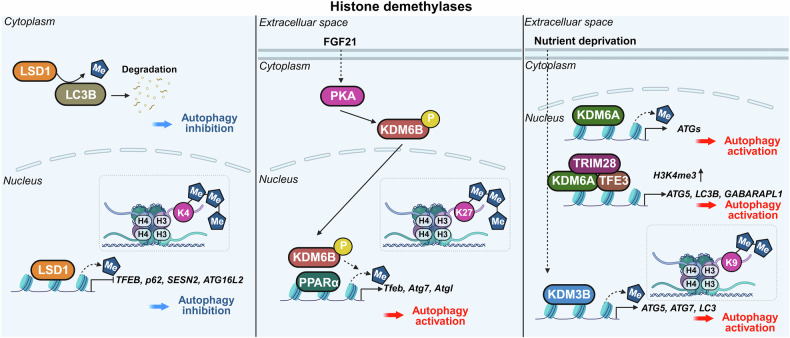
Epigenetic regulation of autophagy by histone demethylases. This figure illustrates the role of histone demethylases in modulating autophagy through the removal of methyl marks on specific lysine residues of histones, thereby altering the chromatin accessibility and transcription of ATGs. (Left) Lysine-specific demethylase 1 (LSD1): LSD1 regulates autophagy through chromatin remodeling and posttranslational modification of histone and nonhistone proteins. In the nucleus, LSD1 demethylates activating H3K4me3 marks at the promoters of ATGs, such as *TFEB, p62, SESN2*, and *ATG16L2*, suppressing their expression and inhibiting autophagic flux. At the protein level, LSD1 directly demethylates LC3B in ovarian cancer cells, promoting its proteasomal degradation and suppressing autophagy. (Middle) KDM6B: KDM6B (also known as JMJD3) is a Jumonji domain-containing histone demethylase that targets the repressive H3K27me3 mark. KDM6B facilitates the transcriptional activation of ATGs, including *Tfeb, Atg7*, and *Atgl*, by removing H3K27me3, especially under conditions such as fasting, inflammation, or cancer. (Right) KDM6A and KDM3B: KDM6A similarly removes H3K27me3 to promote autophagy gene transcription and can also function through nonenzymatic mechanisms by forming complexes (such as with TFE3 and TRIM28) to increase H3K4me3 deposition at autophagy loci. KDM3B regulates autophagy by demethylating H3K9me2, another repressive histone modification. It is recruited to the promoters of autophagy genes, such as *ATG5, ATG7, and LC3*, under nutrient-deprived conditions, where it enhances transcription and promotes autophagosome formation. The figure was created with BioRender.com

Histone demethylases regulate autophagy by removing methyl groups from lysine residues on histones, thereby modifying chromatin structure and transcriptional accessibility at ATG loci. On the basis of the histone mark removed and the biological context, these enzymes function as either activators or repressors of autophagy. Dysregulation of histone demethylases alters the transcription of ATGs, contributing to pathological conditions such as cancer, metabolic disorders, and inflammatory diseases (Fig. 12).

#### LSD1

LSD1 is a flavin-dependent enzyme that demethylates H3K4me3, a histone marker typically associated with transcriptional activation. In hepatocytes, LSD1 is recruited by SHP as part of the FGF19–SHP–LSD1 axis to the *Tfeb* promoter. There, it demethylates H3K4me2/3, creating a repressive chromatin environment and downregulating lysosomal and ATG expression.^189^ In reproductive tissues, LSD1 represses autophagy in oocytes by downregulating *SQSTM1/p62*, preventing premature follicular loss.^315^ In granulosa cells, LSD1 demethylates the *ATG16L2* promoter, suppressing its expression during follicular maturation.^316^ In neuroblastoma, LSD1 inhibits *SESN2*, an mTORC1 inhibitor. Its blockade restores *SESN2*, suppresses mTORC1, and activates autophagy.^317^ LSD1 inhibitors such as 2-PCPA and GSK-LSD1 induce autophagy in tumor cells by increasing LC3-II levels and degrading p62, independent of *BECN1* or *ULK1* signaling.^318^ At the protein level, LSD1 demethylates LC3B in ovarian cancer, promoting its degradation and suppressing autophagy—a process linked to poor prognosis.^319^ Thus, LSD1 suppresses autophagy through H3K4 demethylation and LC3B destabilization, and its inhibition has therapeutic potential in oncology and reproductive medicine.

#### KDM6B (JMJD3)

KDM6B, also referred to as JMJD3, is a JmjC domain-containing demethylase that removes the repressive H3K27me3 mark, facilitating the transcriptional activation of ATGs. In liver cells, KDM6B participates in fasting-induced autophagy through FGF21 signaling. Phosphorylation by PKA promotes complex formation with PPARα, enabling H3K27me3 demethylation at the *Tfeb, Atg7*, and *Atgl* promoters, thereby supporting lipid catabolism through autophagy.^320^ In thyroid carcinoma, KDM6B demethylates the *TFEB* promoter, activating the E2F1–KDM6B–TFEB axis, which upregulates *Beclin-1* and *p62* to sustain lysosomal biogenesis and autophagic flux, supporting tumor progression.^321^ KDM6B thus serves as a potent activator of autophagy across metabolic and cancer contexts.

#### KDM6A/UTX

KDM6A (UTX) also demethylates H3K27me3, promoting open chromatin and gene activation. In *Drosophila*, UTX participates in salivary gland programmed cell death by associating with the ecdysone receptor/ultraspiracle complex to demethylate proapoptotic and ATG promoters.^322^ In human renal cell carcinoma, UTX regulates autophagy through nonenzymatic mechanisms. It forms a complex with TFE3 and TRIM28 that enhances H3K4me3, an activating mark, at the promoters of *ATG5, LC3B*, and *GABARAPL1*, thus leading to their transcriptional upregulation. This TRIM28–TFE3–KDM6A pathway facilitates autophagy-dependent tumor cell proliferation.^323^ UTX thus promotes autophagy through enzymatic demethylation and coactivator-mediated gene activation.

#### KDM3B

KDM3B is a histone demethylase that removes H3K9me2, a mark associated with transcriptional repression. In acute myeloid leukemia (AML), KDM3B activates *GABARAPL1*, a gene essential for autophagosome formation. KDM3B loss reduces *GABARAPL1* expression, impairs autophagy, and increases apoptosis sensitivity.^324^ Collectively, these findings reveal that H3K9me2 demethylation by KDM3B is a conserved mechanism that drives the transcriptional activation of ATGs across malignancies.

### Autophagy regulation by ubiquitination of histone and nonhistone substrates

#### Epigenetic and nonhistone ubiquitin regulators of autophagy: Roles of USP44, RNF20, USP13, USP19, and USP33

Histone H2B monoubiquitination (H2Bub1) functions as a repressive chromatin mark at ATG loci. The E3 ubiquitin ligase RNF20 catalyzes H2Bub1, suppressing the transcription of ATG and thereby inhibiting autophagy. In contrast, the deubiquitinating enzyme USP44 removes H2Bub1, thereby promoting chromatin relaxation and transcriptional activation of ATGs such as *LC3* and *Beclin-1*, increasing autophagosome formation and autophagic flux.^325^ This antagonistic relationship between RNF20 and USP44 represents a dynamic epigenetic switch that modulates autophagy under stress or metabolic cues.

In addition to histones, deubiquitinating enzymes regulate autophagy by stabilizing nonhistone components of the autophagy machinery. USP13 and USP19 enhance autophagy initiation by removing K11- and K63-linked ubiquitin chains from Beclin1, thereby stabilizing it.^326^ USP33 promotes autophagy by deubiquitinating RALB, which strengthens its association with Beclin1 and facilitates the assembly of the Beclin1–RALB–EXO84 complex, a key driver of autophagosome nucleation.^327^ These findings highlight how ubiquitin signaling coordinates chromatin-level and protein-level control of autophagy.

### Autophagy regulation by chromatin remodeling complexes

#### SWI/SNF and INO80

Chromatin remodeling complexes such as SWI/SNF and INO80 actively regulate autophagy by altering nucleosome positioning at ATG promoters. The SWI/SNF complex, containing the ATPase subunits BRG1 (SMARCA4) and BRM (SMARCA2), supports the transcriptional accessibility of genes involved in mitochondrial dynamics and autophagy. In cardiomyocytes, BRG1 and BRM maintain mitophagy and mitochondrial function by regulating genes such as *MFN1, DNM1L*, and *PINK1*, thereby preserving cell viability under metabolic stress.^328^ In *Drosophila*, the SWI/SNF homolog Domino interacts genetically with *Atg1* and *Atg8*, influencing growth and stress-induced autophagy responses.^329^ In addition, SWI/SNF complexes are proposed to coregulate lysosomal and ATG transcription through interactions with transcription factors such as TFEB, although further mechanistic insights are needed.^330^

Similarly, the INO80 complex contributes to autophagy regulation through the control of histone variants. Under nutrient-rich conditions, TORC1 signaling activates the Rpd3L HDAC complex, which deacetylates INO80 and H2A.Z, resulting in reduced chromatin accessibility at ATG promoters and the suppression of their expression.^331^ This finding illustrates how nutrient signals can modulate chromatin architecture to repress autophagy. Collectively, SWI/SNF and INO80 exemplify how chromatin remodeling complexes translate metabolic inputs into the transcriptional control of autophagy, directly linking nuclear organization to cellular stress adaptation.

## Noncoding RNA-mediated regulation of autophagy

In addition to transcriptional and chromatin-level mechanisms, noncoding RNAs, particularly miRNAs and long noncoding RNAs (lncRNAs), represent a critical posttranscriptional layer of autophagy regulation. miRNAs modulate autophagy by directly targeting the mRNAs of ATGs or by regulating the transcription factors essential for controlling autophagy. For example, miR-30a inhibits autophagy by targeting *BECN1*, thereby repressing autophagosome formation in cancer cells,^332^ whereas miR-376b suppresses starvation- and rapamycin-induced autophagy through coordinated repression of *ATG4C* and *BECN1*.^333^ In renal cell carcinoma, the lncRNA SNHG1 promotes *ATG7* expression and enhances autophagy, thereby driving tumor progression and conferring resistance to the tyrosine kinase inhibitor sunitinib.^334^ Moreover, miR-144-3p reduces autophagy during *M. tuberculosis* infection by targeting PPARα and ABCA1.^335^

In addition, lncRNAs influence autophagy through multiple mechanisms. High upregulation in liver cancer enhances autophagy by stabilizing SIRT1, which deacetylates and activates FOXO1, thereby promoting the transcription of ATGs.^336^ Moreover, lncRNAs often act as competing endogenous RNAs, sequestering autophagy-suppressive miRNAs to preserve the expression of autophagy-promoting targets.^337^ A growing body of evidence also highlights the broader epigenetic roles of lncRNAs in remodeling chromatin states or forming scaffolds with transcriptional regulators at ATG loci, thereby influencing autophagy under pathological conditions such as gastrointestinal and hepatic cancers.^337,338^ Although not the central focus of this review, acknowledging the contribution of noncoding RNAs provides a more comprehensive perspective on the multilayered regulatory architecture of autophagy.

## Integrative perspective on autophagy regulation

Although the transcriptional and epigenetic regulation of autophagy involves a diverse array of factors, ranging from nutrient-sensing kinases and stress-responsive transcription factors to chromatin remodelers and metabolic regulators, these seemingly disparate elements converge upon several core signaling hubs and transcriptional circuits. Notably, pathways such as the mTOR–AMPK, TFEB–CLEAR, and p62–Keap1–Nrf2 axes act as central nodes that integrate upstream environmental and intracellular cues into coherent autophagic responses. Transcription factors, including TFEB, FOXOs, E2F1, and p53, frequently coregulate overlapping sets of autophagy and lysosomal genes, indicating a shared transcriptional code that governs the degradative capacity of cell. Epigenetic modifiers further fine-tune this landscape by altering chromatin accessibility at ATG loci in response to stress, energy status, or oncogenic signaling.

A unifying theme that emerges from these observations is that autophagy regulation operates through modular yet interconnected programs, with each module responsive to specific physiological states, nutrient deprivation, oxidative stress, hypoxia, inflammation, or metabolic overload. This modularity permits context-dependent tuning, enabling autophagy to function as a dynamic rheostat instead of a binary switch. For example, under fasting conditions, AMPK and PPARα synergistically activate autophagy through transcriptional and epigenetic reprogramming, whereas in inflammatory states, NF-κB and IRF8 may suppress or redirect autophagic flux toward immune-specific outcomes.

This conceptual model reveals that autophagy is not simply a downstream response but a central homeostatic hub that is actively shaped by multiple layers of transcriptional and epigenetic regulation. This framework highlights the importance of transcription factor crosstalk, chromatin state dynamics, and signaling feedback loops in dictating whether autophagy promotes survival, death, differentiation, or immune responses. By dissecting how distinct inputs converge on common regulatory architectures, we gain insights into the strategic use of autophagy by cells as a versatile adaptation system tailored to diverse disease and stress contexts.

## Bridging the transcriptional and epigenetic regulation of autophagy

Transcription factors involved in autophagy regulation often function in addition to gene activation or repression, acting as targets and recruiters of epigenetic modifiers within tightly integrated feedback loops. For example, FOXO transcription factors, which activate ATGs such as *BECN1, ULK1*, and *ATG12*, are precisely regulated through posttranslational modifications. Acetylation by CBP/p300 decreases the DNA-binding ability and nuclear localization of FOXO1 and FOXO3, thereby reducing their transcriptional activity and ability to induce autophagy.^162,339^ In addition, the nuclear export of acetylated FOXOs further inhibits autophagy, highlighting that transcription factor function is profoundly influenced by posttranslational modification mechanisms mediated by epigenetic regulators.^162^

Notably, p53 exemplifies this dual regulation. Its transcriptional activation of ATGs is increased by p300-mediated acetylation, whereas deacetylation by SIRT1 or HDACs suppresses autophagy and redirects p53 signaling toward apoptosis or survival, depending on context.^240,340,341^ TFEB, a master transcriptional regulator of lysosomal biogenesis and autophagy, is similarly regulated. Acetylation at specific lysines increases TFEB activity,^258^ and HDACis such as trichostatin A (TSA) or suberoylanilide hydroxamic acid (SAHA) increase nuclear translocation, increasing ATG expression.^342^

In many cases, transcription factors guide epigenetic enzymes to ATG loci. FOXO3 directly binds the *BECN1* promoter under catabolic conditions such as fasting or muscle atrophy, promoting autophagosome formation.^157,158^ NF-κB (RelA/p65) also binds to *BECN1* regulatory regions during stress or inflammation, enhancing transcription.^207^ These factors act with chromatin modifiers to regulate gene accessibility. For example, KAT5 (Tip60), a histone acetyltransferase, is recruited to the *BECN1* promoter through a FOXO3a–Pontin complex, promoting H4 acetylation and transcriptional activation.^229^ Conversely, SIRT1 facilitates transcriptional activation of the *BECN1* gene by deacetylating histone H4 at lysine 16, thereby establishing a chromatin configuration permissive for autophagy induction under nutrient-deprived conditions.^230^

**Fig. 13:**
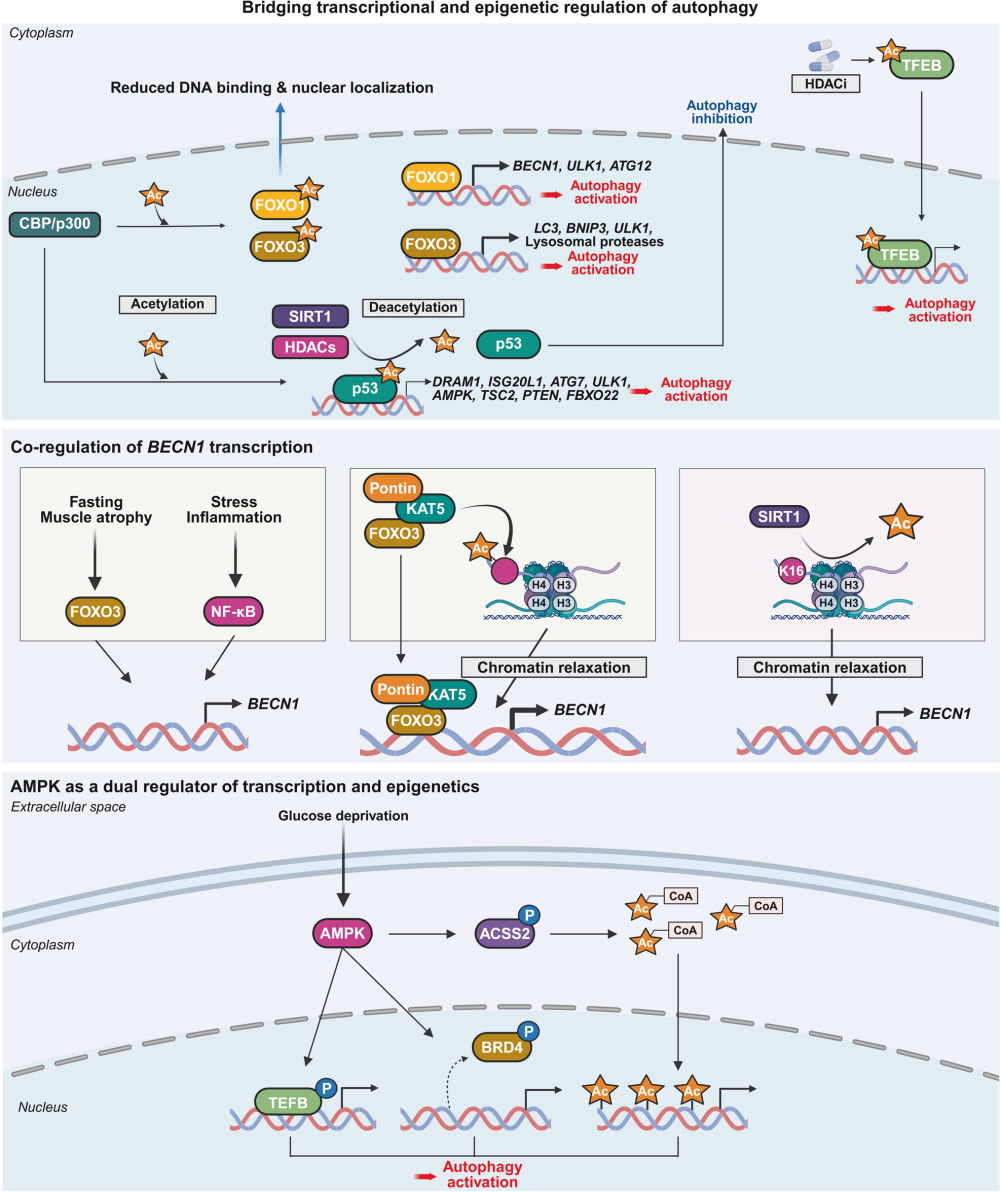
Integrated transcriptional and epigenetic regulation of autophagy. This figure illustrates the interdependent roles of transcription factors and epigenetic modifiers in regulating autophagy, highlighting mechanisms of cooperation, feedback, and nutrient-responsive modulation. (Top) Bridging transcription and epigenetics: FOXO transcription factors (FOXO1/FOXO3) induce the expression of ATGs such as *BECN1, ULK1, ATG12, LC3*, and *BNIP3*. Their transcriptional activity is regulated by acetylation and deacetylation. Acetylation by CBP/p300 reduces nuclear localization and DNA-binding ability, thereby repressing FOXO-driven transcription. In parallel, p53 promotes the transcription of autophagy genes, including *ATG7, DRAM1*, and *ULK1*, upon acetylation, whereas deacetylation suppresses this effect. Histone deacetylase inhibitors (HDACis), such as trichostatin A (TSA) and suberoylanilide hydroxamic acid (SAHA), maintain the acetylation status of specific lysine residues on TFEB, thereby increasing its nuclear translocation and promoting the expression of ATGs. These interactions reveal how posttranslational modifications modulate the transcriptional regulation of autophagy. (Middle) Coregulation of *BECN1* transcription: Transcription of the core autophagy gene *BECN1* is coregulated by FOXO3 and NF-κB under stress, fasting, and inflammatory conditions. FOXO3 directly binds to the *BECN1* promoter and, in cooperation with the histone acetyltransferase KAT5, promotes histone H4 acetylation and chromatin relaxation, facilitating transcription. In parallel, SIRT1 deacetylates histone H4 at lysine 16, establishing a chromatin environment permissive for autophagy induction under nutrient-deprived conditions. The balance between these activating complexes determines BECN1 transcriptional output and autophagic flux. (Bottom) AMPK is a dual regulator of transcription and epigenetics. Under glucose deprivation, AMPK is activated and performs dual regulatory functions. It phosphorylates and activates TFEB, enhancing the expression of autophagy and lysosomal genes. Concurrently, AMPK modulates epigenetic regulators by phosphorylating and inhibiting BRD4, a chromatin reader that represses lysosomal gene transcription, and by activating ACSS2, which increases nuclear acetyl-CoA availability to facilitate histone acetylation. Through these actions, AMPK links the cellular energy status to chromatin dynamics and autophagy-related transcription. The figure was created with BioRender.com

Nutrient-sensing kinases such as AMPK serve as upstream regulators of transcriptional and epigenetic autophagy control. AMPK activates TFEB through phosphorylation, thereby promoting ATG gene expression^343^ while concurrently inhibiting BRD4, an epigenetic repressor of lysosomal genes, to increase transcriptional activity.^219^ In addition, AMPK influences histone acetylation by regulating acetyl-CoA metabolism through enzymes such as ACSS2, linking energy status to chromatin remodeling (Fig. 13).^287,344^

Collectively, these interactions illustrate that the transcriptional and epigenetic regulators of autophagy operate through interdependent mechanisms. This coordinated network enables dynamic, context-sensitive control of autophagy under conditions such as nutrient deprivation, cellular stress, or disease, emphasizing the need to view transcriptional and epigenetic regulation as an integrated system rather than as separate layers.

## Contextual regulation of autophagy in disease

This section presents an integrated overview of how transcriptional and epigenetic regulators govern autophagy across major disease contexts, including cancer, neurodegenerative disorders, metabolic syndromes, and infections. Through this comparative lens, we highlight the context-dependent roles of autophagy and their implications for therapeutic strategies.

In cancer, autophagy plays dual and stage-specific roles. During early tumorigenesis, autophagy preserves genomic integrity and homeostasis by removing damaged organelles and limiting oxidative stress.^14,15^ Transcription factors such as FOXO3 and p53 stimulate autophagy in this context, acting as tumor suppressors.^138,156,160^ However, these pathways are often downregulated or reprogrammed in advanced cancers.^139,144,145^ Epigenetically, EZH2 suppresses ATGs through H3K27me3 deposition, thereby supporting tumor growth and therapy resistance.^299,300^ Consequently, the inhibition of autophagy has emerged as a potential therapeutic strategy in tumors that rely on autophagy for survival and chemoresistance. In such autophagy-dependent cancers, agents such as hydroxychloroquine can sensitize tumor cells to chemotherapy and suppress cancer stemness by disrupting autophagic flux.^9,24^

The context dependency of autophagy modulation is critical. *BECN1* loss enhances tumorigenesis, confirming its protective role in the early stages.^14^ In addition, Atg7 is essential for hematopoietic stem cell maintenance.^345^ In contrast, in advanced tumors, autophagy supports metabolic adaptation under hypoxia or nutrient stress. In BRAF^V600E^-driven lung cancer, autophagy promotes glutamine metabolism and tumor progression.^346^ Similarly, in glioblastoma, hypoxia-induced autophagy mediates resistance to antiangiogenic therapy.^347^ Clinically, high LC3B puncta correlate with increased proliferation, metastasis, and poor prognosis.^348^ These findings highlight the importance of stratifying patients on the basis of disease stage and subtype when considering autophagy-targeted interventions.

In neurodegenerative disorders such as AD, PD, and HD, impaired autophagy leads to the accumulation of toxic protein aggregates.^33,48,53^ Enhancing autophagy in these settings is generally protective. Transcription factors such as TFEB and Nrf2, which promote lysosomal biogenesis and antioxidant responses, are underactive in diseased neurons.^120,127,149^ Epigenetic silencing of ATGs (such as *BECN1* and *LC3*) is frequently observed^33,38^ but can be reversed by HDACis, thereby restoring proteostasis.^243,349^ TFEB activators and SIRT1 agonists show promise in preclinical models.^127,277^

Autophagy also plays tissue-specific roles in metabolic disorders such as T2DM and MAFLD. In hepatocytes, autophagy promotes lipid clearance (lipophagy) and reduces steatosis.^76,185^ In pancreatic β-cells, it mitigates ER stress and promotes insulin secretion.^74^ Transcription factors such as PPARα and FOXO1 upregulate autophagy during fasting^162,186^ but are suppressed postprandially. Epigenetic repression, including DNMT3A-mediated methylation and reduced H4K16ac, downregulates autophagy.^230,305^ Thus, targeted activation of autophagy may improve insulin resistance and hepatic lipid accumulation.^72,75^

In infections, autophagy supports pathogen clearance (xenophagy) and regulates inflammation. Transcription factors such as IRF8 and NF-κB promote autophagy upon microbial challenge.^207,211^ However, pathogens often subvert these processes to evade immunity.^224^ Epigenetically, enzymes such as SETDB1 and HDACs repress ATG expression in infected cells.^8,244^ Therapeutic HDAC inhibitors or inducers such as rapamycin can restore immune autophagy and enhance clearance.^209,350^ Notably, although EZH2 represses autophagy to promote cancer cell survival, HDAC-mediated repression under infection conditions may hinder immune defense, revealing the context-dependent consequences of similar epigenetic mechanisms.

Collectively, these findings emphasize that the outcome of autophagy modulation is dictated by the interplay of disease context, the cellular environment, transcriptional regulators, and epigenetic states. Transcription factors such as PPARγ and C/EBPβ can support autophagy in metabolic and immune contexts but may promote cancer progression or therapy resistance.^351,352^ Similarly, EZH2 suppresses autophagy to drive tumor growth, whereas HDAC inhibition in neurons can reverse ATG silencing to restore proteostasis.^300,353^ In cancer, autophagy suppresses tumor initiation but promotes late-stage progression.^354^ In contrast, familial neurodegenerative disorders, such as inherited PD, often benefit from sustained autophagy activation as a result of persistent proteotoxic stress, whereas sporadic cases may show variable responses depending on the metabolic state and disease progression.^355,356^ These complex, sometimes opposing outcomes necessitate a disease- and stage-specific approach to autophagy modulation.

Recent translational studies support this precise framework. In pancreatic ductal adenocarcinoma, autophagy promotes immune evasion through MHC-I degradation, whereas its inhibition restores antigen presentation and antitumor immunity.^357^ In PD models, TFEB activation enhances autophagic flux and protects neurons from oxidative injury.^358^ Similarly, PPARα activation induces autophagy and improves cognitive outcomes in Alzheimer-like mouse models.^188^ These studies illustrate how the transcriptional control of autophagy can be leveraged therapeutically, provided that interventions are informed by a disease-specific molecular context.

In summary, the transcriptional and epigenetic regulation of autophagy is highly context sensitive. Effective therapies must promote autophagy in degenerative and metabolic diseases while carefully suppressing it in advanced cancers. Precision strategies, which are based on transcriptional profiles, epigenetic marks, and disease staging, are critical for maximizing the therapeutic potential of autophagy modulation.

## Application of transcription factors and epigenetic therapies in autophagy-related diseases

Chemical compounds targeting transcription factors or epigenetic regulators have been explored as potential treatments for autophagy-associated diseases, including various cancers, CVDs, neurodegenerative disorders, and metabolic conditions. Several drugs modulate autophagy and alleviate disease symptoms, as summarized in Tables 3 and 4.

**Table 3:** Drugs targeting autophagy-related transcription factors in diseases

Target	Drugs	Diseases	Autophagy regulation	Mechanism	Drug development status	Clinical trial	FDA approval
TFEB	Eltrombopag	Glioblastoma	Inhibition	Inhibits TFEB- DNA interaction^359^	FDA- approved (repurposed)	-	Approved for thrombocytopenia in chronic immune thrombocytopenic purpura
	Trehalose	Motoneuron degeneration	Activation	Induces TFEB nuclear translocation^360^	Tool compound	NCT05597436, NCT05136885	-
	Desloratadine	Hepatic steatosis	Activation	Induces TFEB nuclear translocation^361^	FDA- approved (repurposed)	-	Approved for allergic rhinitis
BRD4	JQ1	Spinal cord injury (SCI)	Activation	Induces ATG expression^362^	Tool compound	-	-
	JQ1	Diabetic cardiomyopathy	Activation	Promotes PINK1/Parkin-mediated mitophagy^363^	Tool compound	-	-
	JQ1	Cancer	Activation	Induces ferritinophagy-dependent ferroptosis^364^	Tool compound	-	-
FXR	Chenodeoxycholic acid (CDCA), Obeticholic acid (OCA)	Cholestasis	Inhibition	Upregulates Rubicon expression^365^	FDA- approved (repurposed)	NCT02308111	CDCA: approved for cerebrotendinous xanthomatosis; OCA: initially approved for primary biliary cholangitis (accelerated), but full approval was denied
PPARα	Wy-14643	Acute liver failure (ALF)	Activation	Induces autophagy to suppress inflammation^187^	Tool compound	-	-
	Gemfibrozil, Wy-14643	Alzheimer's disease (AD)	Activation	Reduced Aβ accumulation^188^	FDA- approved (repurposed)	NCT02045056	Gemfibrozil: approved for hypercholesterolemia
NF-kB	Curcumin	NSCLC	Activation	Induces ferroptosis through autophagy activation^366^	Tool compound	NCT02321293	-
	Curcumin	Hepatic fibrosis	Activation	Activates PPARα and regulates AMPK and PI3K/AKT/mTOR pathways^367^	Tool compound	NCT03864783	-
	Curcumin	Atherosclerosis	Activation	Induces TFEB nuclear translocation to enhance autophagic flux^368^	Tool compound	-	-
	Curcumin	Neurodegenerative disease	Activation	Inhibits mTOR/p70S6K signaling^369^	Tool compound	NCT01001637, NCT00099710, NCT00164749	-
STAT	AG490	Ovarian cancer	Activation	Increases LC3 and Beclin-1 expression^370^	Tool compound	-	-

**Table 4:** Drugs targeting autophagy-related epigenetic regulators in diseases

Target	Drugs	Diseases	Autophagy regulation	Mechanism	Drug development status	Clinical trial	FDA approval
KAT8	Compound19/34	Non-small cell lung cancer (NSCLC), Acute myeloid leukemia (AML)	Activation	Increased LC3-II levels and enhances autophagic flux^373^	Tool compound	-	-
p300	A-485	NSCLC	Activation	Increased ROS accumulation and DNA damage^374^	Tool compound	-	-
	Nordihydroguaiaretic acid (NDGA)	Aging	Activation	Upregulates *NBR1, p62*, and LC3-II^375^	FDA- approved (repurposed)	-	Approved for actinic keratosis
HDACs	Trichostatin A (TSA)	Cardiac hypertrophy	Inhibition	-	Tool compound	-	-
	Trichostatin A (TSA)	Colorectal cancer (CRC), Hepatocellular carcinoma (HCC), Neuroblastoma	Activation	Induces ATG expression^376,377^	Tool compound	-	-
	Suberoylanilide hydroxamic acid (SAHA,Vorinostat)	Hepatocellular carcinoma (HCC), Cervical cancer	Activation	Inhibits Akt/mTOR signaling and promotes Parkin acetylation-mediated mitophagy^378,379^	FDA- approved (repurposed)	NCT00127127	Approved for cutaneous T-cell lymphoma (CTCL)
	Suberoylanilide hydroxamic acid (SAHA,Vorinostat)	Cardiac ischemia‒reperfusion injury (IRI)	Activation	Induces autophagy and PGC-1α-mediated mitochondrial biogenesis^380^	FDA- approved (repurposed)	-	Approved for cutaneous T-cell lymphoma (CTCL)
	Valproic acid (VPA)	Myeloid leukemia	Inhibition	Activates mTOR signaling^349^	FDA- approved (repurposed)	NCT00079378	Approved for seizure
	Apicidin	Oral squamous carcinoma	Activation	Increases LC3-II and ATG5 expression^382^	Tool compound	-	-
	Belinostat, Givinostat, Vorinostat, Panobinostat, Romidepsin	Human immunodeficiency virus (HIV) infection	Activation	Activates ULK1 and inhibits mTOR signaling^350^	FDA- approved (repurposed)	NCT01319383, NCT01680094, NCT03041012	Belinostat: relapsed/refractory PTCL; Givinostat: DMD; Vorinostat: CTCL; Panobinostat: multiple myeloma (withdrawn); Romidepsin: CTCL
HDAC6	C1A	Neuroblastoma	Inhibition	Increases LC3-II and disrupts autophagic flux^386^	Tool compound	-	-
	J22352	Glioblastoma	Inhibition	Induces p62 accumulation and disrupts autophagic flux^387^	Tool compound	-	-
	ACY-1215 (Ricolinostat)	Multiple myeloma	Inhibition	Suppresses autophagy by blocking aggresome formation^388^	Tool compound	NCT01583283	-
SIRT	Sirtinol	Breast cancer	Activation	Increased LC3-II and Beclin-1expression^390^	Tool compound	-	-
SIRT1	MHY2245	Ovarian cancer	Activation	Induces autophagy by blocking the PKM2/mTOR pathway^391^	Tool compound	-	-
G9a	BIX-01294	Breast cancer, Head and neck squamous cell carcinoma, Multiple myeloma	Activation	Increases LC3-II and ATG5 levels and inhibits EHMT2 activity^392–394^	Tool compound	-	-
EZH2	UNC1999	Aortic dissection	Activation	Increases the expression of ATG5 and ATG7^301^	Tool compound	-	-
	DZNep	Colorectal cancer (CRC)	Activation	Increased LC3 and Ambra1expression^300^	Tool compound	-	-
	GSK343	Osteosarcoma	Activation	Suppresses expression of EZH2, c-Myc, and H3K27me3^395^	Tool compound	-	-
DNMT1	Azacitidine, Zebularine	Metabolic associated fatty liver disease (MAFLD)	Activation	Induces expression of ATGs^396^	FDA- approved (repurposed)	-	Azacitidine: approved for myelodysplastic syndromes (MDS), juvenile myelomonocytic leukemia (JMML), acute myeloid leukemia (AML)
LSD1	ZY0511	Diffuse large B-cell lymphoma (DLBCL)	Activation	Induces expression of ATGs^397^	Tool compound	-	-

### Targeting transcription factors in autophagy-associated diseases

Transcription factors are pivotal regulators of autophagy, and their pharmacological targeting shows therapeutic potential across a spectrum of diseases. TFEB, a master regulator of autophagy and lysosomal biogenesis, can be modulated pharmacologically. Eltrombopag is an FDA-approved drug that was the first identified small-molecule TFEB inhibitor; it disrupts TFEB–DNA binding and suppresses transcriptional activity, thereby blocking autophagy and sensitizing glioblastoma to temozolomide.^359^ In contrast, trehalose activates TFEB indirectly in neurons by inducing lysosomal membrane permeabilization and stimulating the calcium-dependent phosphatase PPP3/calcineurin, which dephosphorylates TFEB and promotes its nuclear translocation, increasing ATG expression and protecting against protein aggregation in neurodegeneration.^360^ Desloratadine, an FDA-approved antihistamine, has emerged as a novel TFEB agonist that promotes TFEB nuclear translocation and target gene activation. In a hepatic steatosis mouse model, it improved lysosomal function, reduced liver lipid accumulation, and enhanced metabolic outcomes.^361^ BRD4 is another transcriptional target of interest. The inhibitor JQ1 promotes autophagy to support neuronal repair after spinal cord injury^362^, induces mitophagy to prevent diabetic cardiomyopathy^363^ and triggers ferritinophagy-dependent ferroptosis in cancer cells.^364^

FXR agonists such as chenodeoxycholic acid and obeticholic acid suppress hepatic autophagy by preventing autophagosome–lysosome fusion. This occurs through FXR-induced Rubicon, a negative regulator of autophagy. Genetic suppression of Rubicon restores autophagic flux and may ameliorate cholestatic liver disease.^365^

PPARα agonists also modulate autophagy. Wy-14643 activates PPARα and suppresses inflammatory signaling (NF-κBp65, JNK, and ERK), protecting against acute liver failure.^187^ Wy-14643 and gemfibrozil enhance autophagy in microglia and glioma, facilitating Aβ clearance and cognitive improvement in AD models.^188^

Curcumin, a natural NF-κB inhibitor, modulates autophagy in multiple disease contexts. In NSCLC, it promotes ferroptosis through autophagy;^366^ in hepatic fibrosis, it attenuates EMT by regulating oxidative stress.^367^ In addition, it enhances autophagy through the TFEB–P300–BRD4 axis in atherosclerosis, improves vascular health^368^ and preserves autophagic homeostasis in neurodegenerative models.^369^

The STAT inhibitor AG490 influences autophagy in cancer. AG490 induces LC3-II expression and autophagosome formation in ovarian cancer cells and, in combination with resveratrol, further enhances autophagy and apoptosis.^370^

PI3K inhibition by LY294002 reduces AKT activity and FOXO1 phosphorylation, preventing cytoplasmic sequestration and diminishing autophagy.^371^ Notably, cytosolic FOXO1 directly interacts with ATG7 to promote autophagosome formation.^162^ Although LY294002 remains untested in clinical trials, it synergizes with PARP inhibitors in BRCA-deficient TNBC and enhances cisplatin efficacy in pancreatic cancer.^372^

Collectively, these findings reveal that targeting transcription factors such as TFEB, BRD4, FOXO, STATs, NF-κB, and p53 enables precise autophagy modulation. This strategy holds significant therapeutic potential across cancers and neurodegenerative, metabolic, and inflammatory diseases.

### Targeting epigenetic regulators in autophagy-associated diseases

Epigenetic enzymes play key roles in regulating autophagy, and their pharmacological targeting holds therapeutic potential across various diseases. KAT8 inhibitors, including compounds 19 and 34, selectively suppress the proliferation of NSCLC and AML cells without affecting normal cells. These agents increase LC3-II and reduce p62, enhancing autophagic flux and supporting anticancer activity.^373^ A-485, a selective p300/CBP HAT inhibitor, induces ROS accumulation and DNA damage, triggering autophagy-mediated senescence in NSCLC. Blocking autophagy shifts the outcome to apoptosis, highlighting its cytoprotective role.^374^ Similarly, nordihydroguaiaretic acid inhibits p300, reduces H3K27 acetylation, and relieves autophagy repression, indicating its longevity potential.^375^

HDACis are potent modulators of autophagy. TSA inhibits HDAC1/2, suppressing cardiac hypertrophy through autophagy repression,^248^ while also inducing FOXO1-dependent ATG expression.^376^ In neuroblastoma, TSA promotes apoptosis and autophagy, with the latter acting as a survival response.^377^ Vorinostat (SAHA), an FDA-approved CTCL, induces autophagic cell death and reverses drug resistance in hepatocellular carcinoma (HCC),^378^ facilitates Parkin acetylation-dependent mitophagy in cervical cancer,^379^ and protects against ischemia/reperfusion injury by preserving mitochondrial homeostasis in clinical studies.^380^

Vorinostat also reduces human immunodeficiency virus (HIV) replication through autophagy activation in infected macrophages without cytotoxicity,^350^ supporting the findings of clinical trials for HIV latency reversal (such as NCT01365065, NCT01319383, NCT01933594, and NCT01680094).^350^ Although HDACis have neuroprotective effects in preclinical models, clinical trials of autophagy-focused therapies for the treatment of neurodegeneration are lacking.^381^

Valproic acid (VPA) suppresses autophagy, while promoting apoptosis in myeloid leukemia,^349^ whereas apicidin induces both autophagy and apoptosis in OSCC by upregulating LC3-II and ATG5.^382^

Combining HDAC and autophagy inhibitors enhances anticancer efficacy. Panobinostat or vorinostat combined with chloroquine augments apoptosis and promotes ubiquitinated protein accumulation in triple-negative breast cancer.^383^ Compared with regorafenib, clinical trials combining vorinostat and hydroxychloroquine in metastatic CRC (NCT02316340) have shown immune enhancement but reduced progression-free survival,^384^ whereas a separate phase I trial (NCT01023737) confirmed safety in solid tumors.^385^

HDAC6-selective inhibitors modulate autophagy by regulating protein trafficking. C1A disrupts autophagic flux and induces cell death through substrate accumulation,^386^ whereas other HDAC6 inhibitors increase the degradation of protein aggregates and increase immunity in glioblastoma.^387^ ACY-1215 (ricolinostat) impairs aggresome formation and synergizes with proteasome inhibitors in multiple myeloma.^388^ HDAC6 inhibition also promotes α-synuclein clearance and dopaminergic neuron protection, supporting its therapeutic application in neurodegeneration.^389^

In addition, class III HDACs (sirtuins) regulate autophagy. Sirtinol induces autophagic and apoptotic death in breast cancer by upregulating LC3-II and Beclin 1.^390^ MHY2245 activates autophagy in ovarian cancer through PKM2/mTOR inhibition, disrupting energy metabolism.^391^

HMTs such as G9a act as autophagy suppressors. BIX-01294 induces ROS-mediated autophagy-associated death in cancer cells.^392^ In head and neck carcinoma, G9a inhibition upregulates DUSP4, triggering autophagic cell death.^393^ In multiple myeloma, G9a/GLP inhibition enhances autophagy-mediated apoptosis and sensitizes cells to proteasome blockade.^394^

EZH2 inhibitors restore autophagy by derepressing ATGs. UNC1999 promotes autophagy-associated death in VSMCs, potentially influencing aortic dissection.^301^ DZNep induces autophagy and apoptosis in CRC through EZH2 downregulation and LC3/Ambra1 upregulation.^300^ GSK343 suppresses EZH2, c-Myc, and H3K27me3, promoting autophagy and apoptosis in osteosarcoma, while also targeting the FBP1–c-Myc axis.^395^

DNMT1 inhibitors such as azacitidine and zebularine restore autophagy in Kupffer cells by reversing *LC3B, ATG5*, and *ATG7* hypermethylation. This reprograms macrophage polarization and halts MAFLD progression.^396^ LSD1 inhibition by ZY0511 increases H3K4 and H3K9 methylation, activating ATGs and inducing autophagy and apoptosis in diffuse large B-cell lymphoma in vitro and in vivo.^397^

In conclusion, targeting epigenetic regulators such as KAT8, p300/CBP, HDACs, sirtuins, G9a, EZH2, DNMT1, and LSD1 enables disease-specific modulation of autophagy. These agents exhibit translational potential across cancers, metabolic and infectious diseases, cardiovascular injury, and neurodegeneration.

### Advancing autophagy-targeted therapeutics: precision delivery, biomarkers, and combinatorial strategies

Although transcriptional and epigenetic modulators are powerful tools for controlling autophagy, their broad activity profiles raise concerns about off-target effects, particularly in complex diseases such as cancer and neurodegeneration. A key challenge is achieving disease- or context-specific autophagy modulation while minimizing the disruption of unrelated pathways.

Recent innovations in targeted delivery and stress-responsive pharmacology have provided solutions. Brain-penetrant autophagy modulators are being developed for neurodegenerative disorders, where systemic delivery often yields inadequate CNS exposure or toxicity. For example, a brain-permeable TNF-α inhibitor significantly altered hippocampal protein expression in a 3xTg AD model, revealing region-specific feasibility.^398^ Disruption of the RARα-corepressor complex selectively activated CMA in retinal degeneration, conferring neuroprotection without broadly triggering macroautophagy.^399^ In addition, CMA modulation stabilizes neuronal proteostasis and reduces proteotoxic stress, emphasizing the value of pathway-specific autophagy control.^400^

Conditional activation of autophagy under stress-responsive conditions is another emerging approach. Chronic psychological stress dynamically regulates neuronal autophagy and influences depression risk.^401^ In vivo inducible gene systems have also been developed to achieve precise spatial and temporal control of autophagic flux in animal models.^402^

To optimize therapeutic outcomes, future approaches must incorporate biomarker-guided treatment and rational drug combinations. Stratifying patients on the basis of their degree of autophagy dependence is crucial for clinical translation. Tumors with activating mutations in *RAS, BRAF*, or *EGFR* or elevated STAT3 activity are more dependent on autophagy for metabolic maintenance and survival, increasing their sensitivity to autophagy inhibition.^403–408^ These include *KRAS*-mutant pancreatic cancers, BRAF^V600E^-driven brain tumors, and STAT3-high breast cancers.^404–407^ In ovarian cancer, transcriptomic profiling revealed that ATG signatures are predictive of poor prognosis and superior immunotherapy response, thus supporting their use as predictive biomarkers.^409^

In addition to genomic markers, functional indicators of autophagy flux, such as LC3 puncta, p62/SQSTM1 accumulation, and ATG expression, are under investigation as clinical biomarkers.^403,410^ However, additional validation and standardization are needed for routine clinical implementation. In AD, altered serum and CSF levels of autophagy-related proteins, including PINK1, BNIP3L, and TFEB, correlate with disease severity, revealing fluid biomarker potential for patient stratification.^411^ However, no autophagy biomarker has yet achieved clinical validation, indicating a critical translational gap.^410^

Mechanism-based combination strategies may enhance the efficacy of autophagy-targeted therapies. Although direct evidence for autophagy modulation through BRD4 and mTORC1/2 coinhibition is limited, this strategy has shown synergistic tumor cell death in rhabdomyosarcoma by disrupting transcriptional and cytoplasmic signaling simultaneously,^412^ indicating future relevance for autophagy therapies. Similarly, HDACis sensitize tumors to autophagy blockade by altering gene expression and survival pathways, especially in aggressive cancers.^378,379^

Collectively, these advances signify a shift toward precision autophagy modulation, integrating targeted delivery, context-specific activation, biomarker-informed selection, and rational combination strategies. As the multifaceted roles of autophagy are further elucidated, therapies must align with disease-specific molecular profiles and tissue environments to maximize efficacy while minimizing toxicity. Continued progress in diagnostics, pharmacology, and systems biology will be essential to fully elucidate the therapeutic potential of autophagy across diverse diseases.

## Clinical trials of transcription factors and epigenetic therapies for treating autophagy-related diseases

Autophagy is a vital process for maintaining homeostasis and plays major roles in the pathogenesis of many diseases, including cancer, neurodegenerative disorders, and metabolic syndromes. The targeting of transcription factors and epigenetic regulators to modulate autophagy has gained attention as a therapeutic strategy. Several clinical trials have tested drugs that target these pathways. This section reviews trials involving the transcription factors TFEB, PPARα, and NF-κB and epigenetic therapies, particularly HDACis, with a focus on their effects on the modulation of autophagy (Tables 5 and 6). Although many agents have been shown to modulate autophagy in preclinical models, direct assessment in human clinical samples has rarely been performed. In most cases, evidence of autophagy modulation is inferred from preclinical data instead of being validated directly in clinical settings.

**Table 5:** Clinical trials of drugs targeting transcription factors involved in autophagy

Target	Drugs	Clinical trial	Diseases	Official title	Current status	Reference
TFEB	Trehalose	NCT05597436	Amyotrophic lateral sclerosis (ALS)	An Expanded Access Protocol of Intravenous Trehalose Injection 90.5 mg/mL Treatment of Patients with Amyotrophic Lateral Sclerosis	No longer available	Unposted
	Trehalose	NCT05136885 (Phase Ⅱ, Ⅲ)	ALS	HEALEY ALS Platform Trial - Regimen E SLS-005 – Trehalose	Completed	Posted (No publication)
PPARα	Gemfibrozil	NCT02045056 (Early Phase Ⅰ)	Alzheimer's disease (AD)	Modulation of Micro-RNA Pathways by Gemfibrozil in Predementia Alzheimer Disease	Completed	Unposted
NF-κB	Curcumin	NCT01001637	AD	Phase II Study of Curcumin Formulation (Longvida) or Placebo on Plasma Biomarkers and Mental State in Moderate to Severe Alzheimer's Disease or Normal Cognition	Terminated	Unposted
	Curcumin	NCT00099710 (Phase Ⅱ)	AD	A Phase II, Double-Blind, Placebo-Controlled Study of the Safety and Tolerability of Two Doses of Curcumin C3 Complex Versus Placebo in Patients with Mild to Moderate Alzheimer's Disease^415^	Completed	Unposted
	Curcumin	NCT00164749 (Phase Ⅰ, Ⅱ)	AD	A Pilot Study of Curcumin and Ginkgo for Treating Alzheimer's Disease^413^	Completed	Posted
	Curcumin	NCT04654689 (Phase Ⅱ)	ALS	Impact of the Combined Treatment of Curcumin and Resveratrol Liposomed Polyphenols with G04CB02 on the Clinical Improvement of ALS Patients	Completed	Unposted
	Curcumin	NCT01514370 (Phase Ⅱ)	Multiple sclerosis	ProspeCtive Study to Evaluate Efficacy, Safety and tOlerability of dietary supplemeNT of Curcumin (BCM95) in Subjects with Active Relapsing MultIple Sclerosis Treated with Subcutaneous Interferon Beta 1a 44 mcg Three Times a Week (TIW) ^414^	Completed	Posted
	Curcumin	NCT02321293 (Phase Ⅰ)	Non-small cell lung cancer (NSCLC)	A Phase 1 Open-label Prospective Cohort Trial of Curcumin Plus Tyrosine Kinase Inhibitors for Epidermal Growth Factor Receptor (EGFR)-Mutant Advanced Non-small Cell Lung Cancer	Unknown	Unposted
	Curcumin	NCT01968564	Vascular aging	Clinical Translation of Curcumin Therapy to Treat Arterial Aging	Unknown	Unposted
	Curcumin	NCT03534024	Metabolic syndrome	The Effects of Nanomicelles Curcumin on Glycemic Control, Serum Lipid Profile, Blood Pressure and Anthropometric Measurements in Patients with Metabolic Syndrome	Unknown	Unposted
	Curcumin	NCT03514667	Metabolic syndrome	The Effects of Nanomicielle Curcumin on Oxidative Stress, Systemic Inflammation, Adiponectin in Serum and NF-kB in Blood Mononuclear Cells, in Patients with Metabolic Syndrome	Unknown	Unposted
	Curcumin	NCT03864783	Metabolic dysfunction-associated fatty liver disease (MAFLD)	The Effect of Curcumin on Liver Fat Content in Obese Subjects	Completed	Unposted

**Table 6:** Clinical trials of drugs targeting epigenetic regulators involved in autophagy

Target	Drugs	Clinical trial ID	Diseases	Official title	Current status	Clinical results
HDACs	Vorinostat	NCT00127127 (Phase Ⅰ)	Solid tumors	A Study of Vorinostat in Patients With Solid Tumors (MK-0683-029)^460^	Completed	Posted
	Vorinostat	NCT01365065 (Phase Ⅱ)	Human immunodeficiency virus (HIV) infection	A Pilot Study to Assess the Safety and Effect on HIV Transcription of Vorinostat in Patients Receiving Suppressive Combination Anti-retroviral Therapy^416^	Unknown	Posted
	Vorinostat	NCT01319383 (Phase Ⅰ, Ⅱ)	HIV infection	A Phase I/II Investigation of the Effect of Vorinostat (VOR) on HIV RNA Expression in the Resting CD4+ T Cells of HIV-Infected Patients Receiving Stable Antiretroviral Therapy^417^	Completed	Posted
	Vorinostat	NCT02707900(Phase Ⅰ)	HIV infection	A Pilot Trial of the Effect of Vorinostat and AGS-004 on Persistent HIV-1 Infection (The VOR VAX Study)	Terminated	Unposted
	Vorinostat	NCT03212989 (Phase Ⅰ)	HIV infection	A Phase I Study to Evaluate the Effects of Vorinostat and HIV-1 Antigen Expanded Specific T Cell Therapy (HXTC) on Persistent HIV-1 Infection in HIV-Infected Individuals Started on Antiretroviral Therapy (The XTRA Study)	Completed	Posted (No publication)
	Vorinostat	NCT03803605 (Phase Ⅰ)	HIV infection	Combination Therapy with the Novel Clearance Modality (VRC07-523LS) and the Latency Reversal Agent (Vorinostat) to Reduce the Frequency of Latent, Resting CD4+ T Cell Infection (The VOR-07 Study)^418^	Completed	Posted
	Vorinostat	NCT03382834 (Phase Ⅱ)	HIV infection	Selective Estrogen Receptor Modulators to Enhance the Efficacy of Viral Reactivation with Histone Deacetylase Inhibitors^419^	Completed	Posted
	Vorinostat	NCT03198559 (Phase Ⅰ, Ⅱ)	HIV infection	Combination Latency Reversal with High Dose Disulfiram Plus Vorinostat in HIV-infected Individuals on ART (DIVA): A Single Arm Clinical Trial^420^	Terminated	Posted
	Vorinostat	NCT02475915 (Phase Ⅰ, Ⅱ)	HIV infection	A Randomized Study to Compare the Efficacy of Vorinostat/Hydroxychloroquine/Maraviroc (VHM) in Controlling HIV After Treatment Interruption in Subjects Who Initiated ART During Acute HIV Infection	Completed	Unposted
	Vorinostat	NCT02336074 (Phase Ⅱ)	HIV infection	Research In Viral Eradication of HIV Reservoirs^422^	Completed	Posted
HDACs	Panobinostat	NCT01680094 (Phase Ⅰ, Ⅱ)	HIV infection	The Safety and Efficacy of The Histone Deacetylase Inhibitor Panobinostat for Purging HIV-1 From The Latent Reservoir (CLEAR) Study^423^	Completed	Posted
	Panobinostat	NCT02471430 (Phase Ⅰ, Ⅱ)	HIV infection	A Phase I-II Pilot Study to Assess the Safety and Efficacy of Combined Administration with Pegylated Interferon-alpha2a and the Histone Deacetylase Inhibitor (HDACi) Panobinostat for Reducing the Residual Reservoir of HIV-1 Infected Cells in cART-Treated HIV-1 Positive Individuals	Completed	Posted (No publication)
	Panobinostat	NCT06240520 (Phase Ⅰ, Ⅱ)	HIV infection	Optimizing Reversal of HIV Latency with Combination Therapy (Pyrimethamine, Lenalidomide, Panobinostat)	Not_Yet_Recruiting	Unposted
HDACs	Romidepsin	NCT01933594 (Phase Ⅰ, Ⅱ)	HIV infection	A Phase I/II Study of Romidepsin in HIV-Infected Adults with Suppressed Viremia on Antiretroviral Therapy to Assess Safety, Tolerability, and Activation of HIV-1 Expression^424^	Completed	Posted
	Romidepsin	NCT02850016 (Phase Ⅱ)	HIV infection	A Phase 2a, Randomized Study of Romidepsin with or Without 3BNC117 to Evaluate the Effects on the HIV-1 Reservoir (ROADMAP)^425^	Completed	Posted
	Romidepsin	NCT02616874 (Phase Ⅰ)	HIV infection	An Open Label Phase I Trial to Evaluate the Safety and Effect of HIVconsv Vaccines in Combination with Histone Deacetylase Inhibitor Romidepsin on the Viral Rebound Kinetic After Treatment Interruption in Early Treated HIV-1 Infected Individuals (BCN02-Romi)^426^	Completed	Posted
HDACs	VPA	NCT00326170 (Phase Ⅱ)	Acute myelogenous leukemia	Phase II Study of the Combination of 5-azacytidine With Valproic Acid and All-trans Retinoic Acid in Patients with High Risk Myelodysplastic Syndrome and Acute Myelogenous Leukemia^430^	Completed	Posted
	VPA	NCT00079378 (Phase Ⅰ)	Acute myeloid leukemia (AML)	A Phase I Study of Decitabine in Combination with Valproic Acid in Patients with Selected Hematologic Malignancies^431^	Completed	Posted
	VPA	NCT06199557 (Phase Ⅰ, Ⅱ)	AML	A Phase 1/2 Multicenter Open-label Study to Investigate Treatment of Hydroxyurea in Combination with Valproic Acid (VPA), or 6- Mercaptopurine in Combination with VPA in Patients with AML or HR-MDS Unfit for Standard Therapy	Not_Yet_Recruiting	Unposted
	VPA	NCT01369368 (Phase Ⅰ, Ⅱ)	AML	Treatment of Relapsed Acute Leukemia After Allogeneic Stem Cell Transplantation: Disease Stabilization Through Chemotherapy, Immunomodulatory Treatment and Immunotherapy	Unknown	Unposted
	VPA	NCT00075010 (Phase Ⅰ, Ⅱ)	Leukemia	Phase I/II Study of 5-aza-2'-Deoxycytidine and Valproic Acid in Patients with Relapsed/Refractory Leukemia or Myelodysplastic Syndromes^432^	Completed	
	VPA	NCT01016990 (Phase Ⅱ)	Leukemia	Epigenetic Therapy with Valproic Acid, an HDAC Inhibitor, in Refractory/Relapsed Non-Hodgkin Lymphoma, Hodgkin's Disease and CLL	Unknown	Unposted
	VPA	NCT03885947 (Phase Ⅰ)	Acute leukemia	Phase I Study of Valproic Acid Expanded Cord Blood Stem Cells as an Allogeneic Donor Source for Adults with Hematological Malignancies	Completed	Unposted
HDAC6	ACY-1215 (Ricolinostat)	NCT01323751 (Phase Ⅰ, Ⅱ)	Multiple myeloma	A Phase 1/2, Open-Label, Multicenter Study of ACY-1215 Administered Orally as Monotherapy and in Combination with Bortezomib and Dexamethasone for the Treatment of Relapsed or Relapsed/Refractory Multiple Myeloma	Completed	Unposted
	ACY-1215 (Ricolinostat)	NCT01997840 (Phase Ⅰ, Ⅱ)	Multiple myeloma	A Phase 1B/2 Multi-Center, Open Label, Dose-Escalation Study to Determine the Maximum Tolerated Dose, Safety, and Efficacy of ACY-1215 (RICOLINOSTAT) in Combination with Pomalidomide and Low-Dose Dexamethasone in Patients with Relapsed and Refractory Multiple Myeloma	Terminated	Unposted
	ACY-1215 (Ricolinostat)	NCT02189343 (Phase Ⅰ)	Multiple myeloma	A Phase 1b Multi-Center, Open Label, Dose-Escalation Study to Determine the Maximum Tolerated Dose, Safety, and Anti-Tumor Activity of an Alternative Liquid Formulation of ACY-1215 (Ricolinostat) In Combination with Pomalidomide and Low-Dose Dexamethasone In Patients with Relapsed and Refractory Multiple Myeloma	Completed	Unposted
	ACY-1215 (Ricolinostat)	NCT01583283 (Phase Ⅰ)	Multiple myeloma	A Phase 1/2, Open-Label, Multicenter Study of ACY-1215 (Ricolinostat) in Combination with Lenalidomide and Dexamethasone for the Treatment of Relapsed or Relapsed/Refractory Multiple Myeloma^433^	Completed	Posted
HDAC6	ACY-241 (Citarinostat)	NCT02551185 (Phase Ⅰ)	Advanced solid tumors	A Phase 1b Study of the Safety, Pharmacokinetics, and Preliminary Antitumor Activity of ACY 241 in Combination with Paclitaxel in Patients with Advanced Solid Tumors^434^	Completed	Posted

### Transcription factors: TFEB, PPARα, and NF-κB

TFEB, a master regulator of autophagy and lysosomal biogenesis, has emerged as a promising therapeutic target. Several compounds have activated TFEB in preclinical models; however, no TFEB-specific agonists have yet reached clinical trials. Trehalose, an indirect TFEB activator, has been evaluated in amyotrophic lateral sclerosis (NCT05136885 and NCT05597436); however, the results remain pending. Although these studies reveal the therapeutic potential of TFEB, clinical validation is incomplete because of limited pharmacokinetic data and uncertain long-term effects.

PPARα, another transcription factor involved in autophagy and lipid metabolism, has also entered clinical evaluation. The PPARα agonist gemfibrozil was assessed in AD (NCT02045056), but the results remain unreported. This reflects a broader challenge: although mechanistic studies in preclinical models are encouraging, translating them into clear clinical benefits remains difficult.

NF-κB, which suppresses autophagy under inflammatory conditions, has been extensively investigated. Curcumin, a natural NF-κB inhibitor, has undergone multiple clinical trials for neuroinflammatory diseases.^413,414^ However, in a placebo-controlled AD trial, Curcumin C3 Complex® failed to improve clinical or biochemical outcomes, likely because of poor bioavailability and rapid metabolism.^415^ To overcome this, improved formulations such as liposomal and nanomicellar curcumin have been tested in early trials (NCT01514370 and NCT03534024),^414^ although findings are pending. In a trial for relapsing multiple sclerosis, liposomal curcumin coadministered with IFN β-1a modestly reduced radiological inflammation,^414^ thereby revealing potential. However, variable outcomes and formulation issues continue to limit translation and require further study.

Collectively, these findings highlight the complexity of translating transcription factor–targeted autophagy modulation into clinical benefit. Although TFEB and PPARα remain mechanistically attractive, the absence of potent clinical-grade modulators and limited trial data restrict their utility. Despite extensive studies with curcumin, NF-κB inhibition highlights challenges in terms of delivery and pharmacokinetics. In the future, integrating pharmacodynamic assessments, optimized delivery systems, and biomarker-guided trial designs will be essential to enhance efficacy.

### Epigenetic therapies targeting HDACs

HDACis represent some of the most extensively studied epigenetic modulators of autophagy, as they promote chromatin relaxation and modify nonhistone proteins involved in autophagy regulation. Their clinical applications span infectious diseases and cancer, although challenges in context-specific efficacy persist.

In HIV therapy, HDACis have been investigated as latency-reversing agents (LRAs) in the “kick and kill” strategy. Vorinostat consistently reactivated latent HIV transcription in ART-suppressed individuals across several trials (NCT01365065, NCT01319383, NCT03803605, and NCT03382834) but failed to meaningfully reduce the viral reservoir.^416–422^ Panobinostat showed similar reactivation with transient viremia (NCT01680094),^423^ whereas romidepsin also elevated HIV-1 RNA expression and immune activation (NCT01933594 and NCT02850016).^424–428^ Despite these effects, long-term viral control was not achieved, reinforcing the need for combination strategies involving bNAbs or vaccines.^418,425^ Moreover, recent studies indicate that the timing of HDACi administration relative to ART initiation could influence reservoir depletion.^425^

In oncology, HDACis have produced mixed outcomes. VPA, a pan-HDACi, has been tested alone or with agents such as ATRA and 5-azacitidine in AML (NCT00995332 and NCT00079378). These trials revealed the modulation of leukemic gene expression and disease stabilization in some patients,^429–432^ although neurotoxicity and limited monotherapy efficacy emphasized the need for rational combination therapy and stratification. To reduce toxicity and improve specificity, newer HDACis have focused on isoform selectivity. HDAC6 inhibitors such as ricolinostat (ACY-1215) and citarinostat (ACY-241) have favorable safety profiles and preliminary efficacy. In a phase 1b trial (NCT01583283), ricolinostat with lenalidomide and dexamethasone yielded a 55% overall response rate in patients with relapsed/refractory multiple myeloma.^433^ Similarly, citarinostat with paclitaxel has shown acceptable safety and initial antitumor activity in advanced solid tumors (NCT02551185).^434^

Despite their ability to induce autophagy, HDACis have not achieved substantial clinical benefit as standalone therapies. In cancer, HDACi-induced autophagy may paradoxically support resistance, necessitating combination with late-stage autophagy inhibitors such as chloroquine. The dual roles of autophagy, which promotes survival or cell death, require precise modulation that broad-spectrum HDACis may not provide. To improve outcomes, combining HDACis with immunotherapies or autophagy inhibitors is a promising direction. Isoform-selective inhibitors may reduce off-target toxicity and enhance tolerability. Patient stratification using predictive biomarkers will be crucial for identifying those most likely to benefit from HDAC-based autophagy modulation.

Clinical trials targeting autophagy through transcription factors and epigenetic regulators have shown potential but also revealed major translational barriers. TFEB, PPARα, and NF-κB remain mechanistically promising but face challenges in terms of clinical efficacy and drug development. HDACis show greater pharmacodynamic activity but have achieved limited success as monotherapies, partly because of the context-dependent roles of autophagy. Future progress will depend on optimized drug design, strategic combinations, and biomarker-guided trial frameworks to fully realize the potential of autophagy-modulating therapies across disease landscapes.

## Pharmacological autophagy activators: mTOR inhibitors and AMPK/SIRT1 activators

Autophagy activators that target nutrient-sensing pathways, such as the mTOR and AMPK/SIRT1 pathways, have attracted interest because of their mechanistic links to aging, metabolism, and cellular homeostasis. However, clinical efficacy varies on the basis of disease context and adverse effect profiles.

mTOR inhibitors, including rapamycin and its analogs (rapalogs), act as indirect autophagy inducers by inhibiting mTORC1 and releasing ULK1. Everolimus is approved for advanced renal cell carcinoma on the basis of improved progression-free survival in a phase III trial (NCT00410124).^435^ Sirolimus has also shown efficacy in rare diseases such as lymphangioleiomyomatosis (NCT00414648) and renal angiomyolipoma in tuberous sclerosis complex (TSC) (NCT00457808),^436,437^ where mTOR-driven cell proliferation and dysregulated autophagy are key features.

In neurodegenerative disease, the ERAP phase IIa trial (NCT06022068) is evaluating rapamycin for AD.^438^ Despite their promise, mTOR inhibitors have shown limited progress in the treatment of metabolic diseases owing to side effects such as hyperlipidemia, diabetes, and impaired wound healing,^439^ limiting broader application in patients with metabolic comorbidities.

In contrast, AMPK and SIRT1 activators have more favorable effects on metabolic and neurodegenerative conditions because they promote autophagy and improve mitochondrial function and insulin sensitivity. Metformin, a widely used AMPK activator, improved memory and executive function in a pilot crossover trial in patients with mild cognitive impairment or early AD (NCT01965756).^440^ Although the sample size was small, the results support the neuroprotective potential of AMPK-mediated autophagy activation. SIRT1, an NAD⁺-dependent deacetylase and autophagy regulator, is another promising target. Resveratrol, a natural SIRT1 activator, has been tested in multiple clinical trials. In a phase I study involving healthy adults (NCT01640197), resveratrol improved cognitive function, cerebral perfusion, mood, and sleep.^441^ Another trial (NCT02114892) revealed that high-dose resveratrol (1,500 mg/day) improved the expression of markers of metabolic syndrome, including those related to glycemic control and lipid profiles. A combined formulation study with resveratrol and hesperetin (NCT02095873) revealed glyoxalase 1 upregulation and improved vascular and metabolic health in obese participants.^442^ However, the rapid metabolism of resveratrol limits bioavailability, prompting the development of sustained-release formulations and combination strategies to increase systemic exposure.

In summary, mTOR inhibitors have revealed disease-modifying potential in cancer and genetic disorders such as TSC, but their use is limited by metabolic toxicity. In contrast, AMPK/SIRT1 activators, especially metformin and resveratrol, show promise in the treatment of cognitive and metabolic disorders with improved safety. These findings highlight the need for context-specific autophagy modulation and indicate that therapeutic success depends on pharmacologic refinement, advanced delivery, and patient stratification.

## Emerging delivery platforms for precision autophagy modulation

Although pharmacological agents targeting autophagy hold substantial therapeutic potential, especially in diseases such as cancer and neurodegeneration, their clinical success has been limited by pharmacokinetic and pharmacodynamic barriers. These include poor aqueous solubility, low systemic bioavailability, a short half-life, and significant off-target toxicity. Recent advances in nanomedicine, biomaterials, and smart drug delivery technologies have provided strategies to overcome these obstacles, enabling disease specific, controlled, and sustained autophagy modulation with improved safety and efficacy profiles.

Nanoparticle-based delivery systems have emerged as powerful tools for targeting autophagy pathways with high spatial and cellular precision. Although FDA-approved for autophagy inhibition, traditional agents such as hydroxychloroquine exhibit diminished lysosomal accumulation and reduced activity in acidic microenvironments, such as those that occur in solid tumors, thereby limiting their in vivo effectiveness.^443^ To address these limitations, nanocarrier formulations have been developed to encapsulate hydroxychloroquine or other autophagy modulators, increasing their solubility, protecting against premature degradation, and improving their biodistribution. For example, codelivery of cisplatin and chloroquine through hybrid dendritic‒linear‒dendritic block copolymer micelles significantly increased cytotoxicity in tumor cells but had minimal effects on normal, nontransformed cells.^444^

Albumin-based nanoparticles have gained traction owing to their biocompatibility, long circulation time, and intrinsic tumor-targeting ability through receptor-mediated uptake mechanisms such as gp60 and SPARC binding. These particles facilitate the efficient delivery of hydrophobic drugs such as rapamycin and AZD8055, leading to robust autophagy activation or inhibition in target tissues. For example, AZD8055 conjugated to albumin-stabilized gold nanoclusters (ABN-AZD) achieved tumor-selective drug release through glutathione-responsive linkers,^445^ whereas albumin-bound rapamycin nanoparticles (nab-rapamycin, ABI-009) enhanced tumor accumulation and reduced systemic toxicity.^446–449^

Lysosome-targeting nanomedicines represent another frontier in precision autophagy modulation. One example is the use of self-assembling lysosomotropic nanoparticles formed from the combination of the potent autophagy inhibitor Lys-05 and the detergent MSDH, resulting in improved pharmacokinetics and superior antitumor efficacy in preclinical models.^450^ This strategy enables direct manipulation of the lysosomal compartment, the core machinery of the autophagic pathway, providing mechanistic specificity and therapeutic potency.

Metal-based nanocarriers further expand the toolkit by enabling external activation methods such as magnetic hyperthermia. Metallic nanoparticles can be guided and locally heated via magnetic fields or lasers, selectively triggering autophagy or immunogenic cell death at tumor sites without affecting surrounding healthy tissues.^451,452^ This spatiotemporal precision is particularly valuable in solid tumors where regional therapy is needed.

Liposomal and polymeric nanoparticles have also been applied to increase the delivery of autophagy regulators to the brain. Biomimetic nanoparticles encapsulating rapamycin have been shown to efficiently penetrate the blood‒brain barrier, induce protective autophagy and reduce oxidative stress in autism and glioblastoma models.^453,454^ These systems ensure that therapeutic concentrations of autophagy modulators reach affected brain regions while minimizing peripheral toxicity.

Finally, biomaterial-based platforms, such as injectable hydrogels and implantable depots, provide innovative solutions for chronic and site-specific autophagy modulation. These systems can be engineered for sustained or stimuli-responsive drug release, reducing dosing frequency and systemic exposure. For example, rapamycin-loaded hydrogels have shown long-term antifibrotic and antiangiogenic effects in models of ocular injury, vascular remodeling, and age-related macular degeneration.^455–457^

Collectively, these cutting-edge delivery strategies dramatically improve the pharmacological performance and disease-targeting capabilities of autophagy modulators. By integrating nanotechnology and biomaterials with rational drug design, researchers are better equipped to harness the therapeutic potential of autophagy modulation in pathological contexts ranging from malignancies to neurodegeneration and chronic inflammatory diseases.

## Conclusions and perspectives

Although prior reviews have laid important groundwork in understanding the transcriptional and epigenetic control of autophagy, most notably the foundational contributions by Füllgrabe et al.^8^ and Feng et al.^458^ and more disease-oriented perspectives by Lei et al.^7^ and Metur et al.^459^, this review advances the field by providing an updated and mechanistically enriched synthesis. It provides a curated and functionally grouped catalog of over 20 transcription factors and chromatin regulators, including emerging players such as ZKSCAN3, ATF5, and members of the GATA and C/EBP families, which were previously underrepresented.^7,8,458,459^ These factors are classified into regulatory modules—metabolic, immune/inflammatory, stress-responsive, and oncogenic—to emphasize how they converge on autophagy-related signaling axes such as the mTOR–AMPK, TFEB–CLEAR, and p62–Keap1–Nrf2 loops. Unlike prior reviews that focus on single disease contexts or limited pathways,^7,459^ this work integrates autophagy regulation across diverse physiological and pathological conditions, including cancer, neurodegenerative diseases, cardiovascular and metabolic syndromes, muscular atrophy, and aging-related degeneration. In doing so, we highlight the context-dependent plasticity of autophagy and how its regulatory architecture adapts dynamically to the metabolic, inflammatory, and redox environments of the cell.

A central feature of autophagy regulation is its dependence on transcription factors that activate or repress ATG expression in response to diverse intracellular and environmental stimuli. TFEB, for example, orchestrates a broad transcriptional program involved in lysosomal biogenesis and autophagy, integrating nutrient-sensing inputs through phosphorylation-dependent localization. PPARα activates ATGs related to lipid metabolism and stress adaptation, whereas BRD4 and FXR act as repressors under basal or nutrient-rich conditions. ZKSCAN3, C/EBPβ, and SHP play dual regulatory roles that are context specific, reinforcing autophagy as a finely tuned and reversible process.

Notably, the influence of transcription factors extends further than that of the nucleus. Proteins such as FOXO1, STAT3, and p53 also exert autophagy-modulatory effects in the cytoplasm, such as through interactions with initiators, regulating mitochondrial function, or sequestering kinases and adaptors involved in autophagosome formation. This dual nuclear–cytosolic functionality expands the regulatory landscape, indicating that the transcriptional regulation of autophagy must be understood as a spatially dynamic, compartmentalized process.

Epigenetic regulators further contribute to this multilayered control through histone acetylation, methylation, and chromatin remodeling. Acetyltransferases such as KAT5 and p300 increase ATG transcription by opening chromatin, whereas HDAC1/2 and SIRTs suppress autophagy in basal states. HDAC6 and HDAC10 also support autophagy–lysosome fusion, mitophagy, and proteostasis. These enzymes further modulate autophagy by acetylating nonhistone proteins, including ULK1, ATG5, and TFEB.

Methylation marks offer another regulatory axis. EZH2-mediated H3K27me3 silences ATGs in cancers, whereas LSD1, JMJD3, and G9a fine-tune ATG expression in response to stress or inflammation. For example, LSD1 and CARM1 modulate both histone and nonhistone proteins, indicating that epigenetic control operates at multiple regulatory levels. Collectively, these findings support a model in which transcriptional and epigenetic programs form an interconnected, flexible system that dynamically orchestrates autophagy in health and disease.

Translational advances have shown that targeting transcription factors or epigenetic modifiers can modulate autophagy for therapeutic benefit. Eltrombopag, a TFEB inhibitor, reduces autophagy in glioblastoma cells, whereas trehalose, a TFEB activator, enhances lysosomal function and mitigates neurodegeneration. BRD4 inhibitors such as JQ1 promote autophagy-mediated neuronal repair and induce ferroptosis in cancer. FXR agonists, including chenodeoxycholic acid and obeticholic acid, inhibit hepatic autophagy through Rubicon induction, which is relevant for cholestatic liver disease. PPAR agonists (Wy-14643, pioglitazone) increase autophagy and reduce inflammation, steatosis, and the Aβ burden in AD models. Among epigenetic therapies, HDACis such as SAHA and TSA induce autophagy-dependent apoptosis in cancer and exhibit cardioprotective effects by suppressing hypertrophy. G9a, DNMT1, and EZH2 inhibitors reactivate silenced autophagy pathways and sensitize tumors to chemotherapy. These compounds remodel chromatin and restore ATG accessibility, confirming the feasibility of targeting the epigenetic machinery in autophagy-related diseases.

However, clinical translation faces obstacles. Trials of HDACis such as vorinostat and panobinostat in HIV latency reversal have shown strong activation of viral transcription but minimal latent reservoir reduction, highlighting the need for combination strategies. Curcumin, a natural NF-κB inhibitor, has been studied in the context of neurodegenerative and metabolic diseases, but its therapeutic value remains inconsistent owing to its poor bioavailability and rapid metabolism. Nanoparticle and liposomal formulations are being explored to improve systemic delivery and efficacy.

Clinical trials of TFEB activators such as trehalose (NCT05136885 and NCT05597436) and PPARα agonists such as gemfibrozil (NCT02045056) further illustrate the challenges of translating preclinical findings into clinical benefits. A major issue is the absence of direct autophagy readouts at trial endpoints. Without validated biomarkers, such as the LC3-II/I ratio, p62 level, or lysosomal hydrolase activity, the therapeutic effects on autophagy remain unclear. There is an urgent need to develop and standardize such biomarkers in clinical protocols.

Another challenge is the dual nature of autophagy. Although protective against early tumor suppression and neurodegeneration, it can also support tumor survival, immune evasion, and resistance in advanced cancers. It may even exacerbate tissue damage in certain inflammatory settings. Thus, the success of autophagy-targeted therapies hinges on achieving temporal and contextual specificity, activating autophagy where it is protective and inhibiting it where it is harmful.

To overcome these challenges, future research must prioritize biomarker-guided stratification, rational drug combinations, and targeted delivery. For example, autophagy inhibitors may be paired with chemotherapies or checkpoint inhibitors in cancers with elevated autophagy, whereas activators may be combined with metabolic regulators in neurodegenerative disorders. Isoform-selective HDACi and BET domain modulators also hold promise for enhancing therapeutic specificity while minimizing off-target effects.

In conclusion, the transcriptional and epigenetic regulation of autophagy is a rapidly advancing field with notable therapeutic implications. By integrating mechanistic insight with translational progress, it is feasible to envision a new generation of autophagy-targeted therapies tailored to specific disease contexts. This review highlights both the promise and complexity of such strategies, calling for interdisciplinary efforts in basic, translational, and clinical science to fully realize the therapeutic potential of autophagy modulation in human disease.
